# Binding Affinity and Mechanism of Six PFAS with Human Serum Albumin: Insights from Multi-Spectroscopy, DFT and Molecular Dynamics Approaches

**DOI:** 10.3390/toxics12010043

**Published:** 2024-01-05

**Authors:** Mingguo Peng, Yang Xu, Yao Wu, Xuewen Cai, Weihua Zhang, Lu Zheng, Erdeng Du, Jiajun Fu

**Affiliations:** 1 School of Chemistry and Chemical Engineering, Nanjing University of Science and Technology, Nanjing 210094, China; pmg@cczu.edu.cn; 2 School of Environmental Science and Engineering, Changzhou University, Changzhou 213164, China; xuyangcz1998@hotmail.com (Y.X.); wuyao2203@foxmail.com (Y.W.); whitecai05@foxmail.com (X.C.); zwh-cczu1991@hotmail.com (W.Z.); zhenglu@cczu.edu.cn (L.Z.)

**Keywords:** human serum albumin (HSA), per- and polyfluoroalkyl substances (PFAS), multi-spectroscopy, DFT calculations, molecular docking, molecular dynamics simulation

## Abstract

Per- and Polyfluoroalkyl Substances (PFAS) bioaccumulate in the human body, presenting potential health risks and cellular toxicity. Their transport mechanisms and interactions with tissues and the circulatory system require further investigation. This study investigates the interaction mechanisms of six PFAS with Human Serum Albumin (HSA) using multi-spectroscopy, DFT and a molecular dynamics approach. Multi-spectral analysis shows that perfluorononanoic acid (PFNA) has the best binding capabilities with HSA. The order of binding constants (298 K) is as follows: “Perfluorononanoic Acid (PFNA, 7.81 × 10^6^ L·mol^−1^) > Perfluoro-2,5-dimethyl-3,6-dioxanonanoic Acid (HFPO-TA, 3.70 × 10^6^ L·mol^−1^) > Perfluorooctanoic Acid (PFOA, 2.27 × 10^5^ L·mol^−1^) > Perfluoro-3,6,9-trioxadecanoic Acid (PFO3DA, 1.59 × 10^5^ L·mol^−1^) > Perfluoroheptanoic Acid (PFHpA, 4.53 × 10^3^ L·mol^−1^) > Dodecafluorosuberic Acid (DFSA, 1.52 × 10^3^ L·mol^−1^)”. Thermodynamic analysis suggests that PFNA and PFO3DA’s interactions with HSA are exothermic, driven primarily by hydrogen bonds or van der Waals interactions. PFHpA, DFSA, PFOA, and HFPO-TA’s interactions with HSA, on the other hand, are endothermic processes primarily driven by hydrophobic interactions. Competitive probe results show that the main HSA–PFAS binding site is in the HSA structure’s subdomain IIA. These findings are also consistent with the findings of molecular docking. Molecular dynamics simulation (MD) analysis further shows that the lowest binding energy (−38.83 kcal/mol) is fund in the HSA–PFNA complex, indicating that PFNA binds more readily with HSA. Energy decomposition analysis also indicates that van der Waals and electrostatic interactions are the main forces for the HSA–PFAS complexes. Correlation analysis reveals that DFT quantum chemical descriptors related to electrostatic distribution and characteristics like ESP and ALIE are more representative in characterizing HSA–PFAS binding. This study sheds light on the interactions between HSA and PFAS. It guides health risk assessments and control strategies against PFAS, serving as a critical starting point for further public health research.

## 1. Introduction

Per- and Polyfluoroalkyl Substances (PFAS) are a class of compounds composed of fluorinated carbon chains with one or more functional groups [1,2]. These compounds have strong carbon–fluorine bonds, which provide high chemical stability and bio-accumulation potential, as well as an ultra-low surface energy [3,4]. Therefore, PFAS are widely used in industrial and commercial applications, including the production of firefighting foam, non-stick, and stain-resistant materials [5,6]. PFAS have a high hydrophobicity and acidity, allowing for them to persist in the environment for a long time while resisting biodegradation, photolysis and hydrolysis. This property of PFAS increases the possibility of bioaccumulation in the food chain and facilitates long-distance transport via air or water [5]. Currently, PFAS concentrations, ranging from 1 ppt to 1000 ppt, have been detected in a variety of environmental samples in water around the world [7]. According to a global study, the concentration of PFAS in China ranges from 20 to 300 ppt, while concentrations in the United States, the United Kingdom, and Germany range between 16 and 75 ppt in wastewater, surface water, groundwater, and drinking water [8,9]_._

Perfluorooctanoic Acid (PFOA) and Perfluorooctanesulfonic acid (PFOS) were the most extensively used PFAS and are now restricted. These two compounds have attracted widespread attention due to their frequent detection in environmental samples and the human body. Numerous scientific studies have revealed the phenomenon of PFAS bioaccumulating in humans [5]. PFAS have relatively long half-lives in the human body. According to a study on 19 PFAS, the average half-life of PFOA and PFOS was approximately 2.47 and 4.52 years, respectively [10]. Another research also reported that the average half-lives of PFOA and PFOS were 3.8 and 5.4 years, respectively [5]. PFAS are primarily known to accumulate in human blood, liver and kidney, indicating their high affinity for proteins. The prolonged presence of PFAS in the human body can lead to potential cytotoxicity and health risks. PFOS and PFOA have been linked to an increase in total serum cholesterol levels, lowered immunity, and the development of chronic diseases such as Chronic Kidney Disease (CKD), asthma, and Attention Deficit/Hyperactivity Disorder (ADHD) in children. The mechanisms of PFAS transport within the human body, as well as their interactions with human tissues and the blood system, remain subjects for further exploration [11].

Proteins serve as the fundamental building blocks for all forms of life in organisms [12]. As a ligand-binding protein, human serum albumin (HSA) is widely present throughout the blood system, accounting for 60% of protein content [13]. One of its primary functions is to transport endogenous and exogenous ligand compounds between tissues and organs [1]. According to research, HSA is the primary entity that binds to a variety of small molecule compounds, including PFAS [7], picloram [14], and noscapine [15].

PFAS primarily enter the human body through ingestion and inhalation and accumulate as enterohepatic circulation metabolites. This leads to a high concentration of PFAS in the blood, which may induce protein abnormalities, thereby causing physiological dysfunction [1]. As a result, it is critical to thoroughly investigate the interaction of PFAS and HSA in order to comprehend their distribution, metabolism, and toxicity mechanisms in human body [4].

Several PFAS, such as PFOA [7], PFOS [2], PFBS [16] and PFHxS [17], have been chosen as the focus for explorations of HSA–PFAS binding. PFAS with different carbon chain lengths or functional groups have varying binding behaviors with HSA [2]. Most of these studies, however, have primarily focused on traditional PFAS and frequently only involve the interaction of a single PFAS compound with HSA. Furthermore, current research usually employs techniques such as fluorescence spectroscopy and molecular docking [7], resulting in a lack of a comprehensive approach to investigate the binding characteristics of a variety of PFAS with HSA. This research gap has resulted in uncertainty about the key structural features that influence the binding affinity of PFAS under similar binding conditions. Therefore, more in-depth studies are urgently needed, not only to broaden the range of PFAS being studied, but also to employ a variety of analytical techniques to understand the complex interactions between these PFAS and HSA.

This study focuses on the binding interactions between HSA and six common PFAS compounds, as listed in Table 1. All six are perfluorocarboxylic acids, each with one or two carboxyl groups. Among them, two PFAS feature oxygen atoms as ether linkages (-O-) within the carbon chain, representing novel PFAS selected for their distinct structures. The binding characteristics, structural changes, and thermodynamic properties of these HSA–PFAS complexes will be thoroughly investigated using multispectral techniques. These techniques, such as fluorescence quenching, 3D-EEM and UV-visible spectroscopy, are used to not only quantify binding constants and sites, but also to reveal conformational changes in HSA. Furthermore, the electronic structures will be computed using Density Functional Theory (DFT), and molecular docking and kinetic simulations will be used to gain a better understanding of the nature of HSA–PFAS binding. These findings will provide critical scientific evidence for assessing the biological and environmental effects of PFAS.

## 2. Chemical and Process

### 2.1. Chemicals

HSA (≥96%) and six PFAS were all purchased from Aladdin Chemicals (Shanghai, China), including Perfluorooctanoic Acid (PFOA, CAS:335-67-1), Perfluorononanoic Acid (PFNA, CAS:375-95-1), Perfluoro-2,5-dimethyl-3,6-dioxanonanoic Acid (HFPO-TA, CAS:13252-14-7), Perfluoro-3,6,9-trioxadecanoic Acid (PFO3DA, CAS:151772-59-7), Perfluoroheptanoic Acid (PFHpA, CAS:375-85-9) and Dodecafluorosuberic Acid (DFSA, CAS:678-45-5). Three probe substances, including warfarin (≥98%), ibuprofen (≥98%), and lidocaine (≥99%), were also obtained from the same company. PBS buffer (Sigma-Aldrich, St. Louis, MO, USA) was used to prepare HSA stock solution (1 × 10^−6^ mol·L^−1^) to ensure the maintenance of appropriate ionic strength (pH = 7.4) and biocompatibility. Six PFAS aqueous stock solutions were also prepared at a concentration of 1 × 10^−6^ mol·L^−1^ for subsequent binding experiments.

### 2.2. Fluorescence Quenching Experiments

Fluorescence quenching experiments greatly benefit studies on the interactions between ligands and proteins. Initially, 3 mL of HSA stock solution was put into a 10 mm square quartz cuvette. Following that, PFAS stock solution was gradually added to achieve various final concentrations, namely 0, 3, 6, 9, 12, 15, 18 × 10^−6^ mol·L^−1^. By incrementally increasing the molar ratio of PFAS vs. HSA up to 18, their binding characteristics can be better investigated.

A thermostat (TR-01A, Bishui Corp, Beijing, China) was used to precisely control the solution temperature, which was set at 298, 304, and 310 K. This device includes a temperature controller and a metal heating cuvette module in conjunction with a fluorometer. This thermostat has a temperature range of 20–60 °C (293–333 K) and an accuracy of 0.1 °C Celsius. This step is critical for keeping the experimental conditions stable. Following that, the samples were fluorescence-scanned with a fluorometer (Cary Eclipse, Agilent, CA, USA). The excitation wavelength was set at 275 nm to efficiently stimulate tryptophan (Trp) and tyrosine (Tyr) residues [18]. Additional test parameters include an emission of 275–500 nm, scanning rate of 1200 nm/min and PTV voltage of 700 v. Fluorescence quenching experiments were repeated three times, and the average values were taken for further calculation. It is worth noting that none of the six PFAS tested in this study exhibited significant fluorescence signals, indicating that the intrinsic fluorescence properties of PFAS do not interfere with the study of their binding to HSA.

Fluorescence internal filtration (IFE) refers to the phenomenon where the fluorescence intensity decreases during fluorescence measurement due to the absorption of excitation or emission light by sample components (small molecules or proteins) in solution [19,20]. This phenomenon is more obvious in the samples with a high concentration of adsorbent. A fluorescence correction formula was used in this study to correct the IFE effect on the data, as follows:

$$F_{\mathrm{corr}}=F_{\mathrm{obs}}\times 10^{\left(A_{\mathrm{ex}}+A_{\mathrm{em}}\right)/2}$$

where *F*_corr_ and *F*_obs_ are corrected and observed fluorescence emission intensities, respectively, *A*_ex_ and *A*_em_ are UV-vis absorbances at the excitation and emission wavelengths [18].

### 2.3. Spectroscopic Scanning

A UV-vis spectrophotometer (Specord 50, Analytik Jena, Germany) was used with a wavelength of 190–600 nm and 1 nm increments. The UV-vis spectra of single PFAS were subtracted from the UV-vis data to remove the influence of the absorption peaks inherent in PFAS, allowing for a more distinct differentiation of HSA absorption features.

Synchronous fluorescence scanning was set with two wavelength differences of 15 nm and 60 nm at 298 K. Other scanning parameters included an excitation wavelength of 200–400 nm.

The 3D-EEM spectra were recorded using specific scanning parameters at the scanning rate of 2400 nm·min^−1^, an emission of 220–400 nm with 5 nm increments, and an emission of 280–550 nm with 2 nm increments. The concentrations of PFAS and HSA were set at 18 × 10^−6^ mol·L^−1^ and 1 × 10^−6^ mol·L^−1^, respectively.

### 2.4. Circular Dichroism (CD) Spectrum

CD measurements (190–260 nm) were taken before and after the addition of PFAS to HSA using a J-815 CD spectrometer equipped with a PMT detector (JASCO, Tokyo, Japan). The protein concentration was set at 1 × 10^−6^ mol/L, with a fixed HSA-to-PFAS concentration ratio of 1:18. The scanning rate was set to 100 nm/min with 0.5 nm increments, and the photometric mode of HT. Each sample was scanned three times. The blank buffer control was automatically subtracted during the scanning process. All tests were carried out at 298 K. The CONTIN analysis method from the DichroWeb [21] was employed to determine the contents of the protein’s secondary structure.

### 2.5. Competitive Probe Experiment

Competitive probe experiments are commonly used to identify specific binding sites in the structure of proteins. Three probe molecules known to bind to distinct binding sites on HSA were selected: warfarin (subdomain IIA), ibuprofen (subdomain IIIA), and lidocaine (subdomain IIB) [22,23]. These probe molecules would compete with PFAS for the same protein sites when binding with HSA. The potential binding sites can be inferred by monitoring and comparing changes in fluorescence intensity when probe molecules are present. The probe molecules were concentrated at 1 × 10^−6^ mol·L^−1^, with PFAS adding up to 18 × 10^−6^ mol·L^−1^_._

### 2.6. Quantum Chemical Computation

Quantum chemical computations serve as a scientific tool, allowing for a thorough analysis of molecular structure and properties at the microscopic level. The molecular structures of six PFAS were acquired via ChemSpider. The Gaussian 16 [24] software suite was used to perform molecular structure optimization based on the m062x density functional at the 6–31+g(d,p) level, with water as the solvent, in a PCM model [25,26,27]. All optimized structures were further post-processed with MultiWFN 3.7 [28], and several quantum chemistry descriptors were also visualized with VMD 1.9.3 [29] software.

### 2.7. Molecular Docking Studies

Molecular docking of the HSA–PFAS complex was carried out to explore HSA–PFAS binding at the active site [30]. The 3D structure of HSA was acquired via the RSCB database (ID 7JWN). Autodock Vina 1.1.2 [31,32] was used to process HSA and PFAS, which involved removing water molecules, co-crystal ligands and adding polar hydrogens. The molecules were placed in a cubic grid space for molecular docking with a side length of 22.5 Å and set exhaustiveness of 32 for global search. The optimal conformations were analyzed and visualized using PyMol 2.5 [33]. The obtained docking conformations were utilized for subsequent molecular dynamics simulations.

### 2.8. Molecular Dynamics Simulation (MD)

AMBER 18.0 was employed to run a full-atom MD simulation based on the initial structures of HSA–PFAS complexes from the molecular docking presented above [34]. The force fields of GAFF2 and ff14SB were used in the pre-simulation processing to characterize PFAS and HSA, respectively [35,36]. The LEaP module is critical for supplementing the system with missing hydrogen atoms. A TIP3P solvent box was introduced to provide an appropriate solvation environment [37]. Furthermore, a proper amount of Na^+^/Cl^−^ ions was also incorporated into the simulation framework to mimic the electrolytic environment and maintain electro-neutrality in the system.

MD simulations were performed in several steps, including energy minimization, heating, equilibration, production run and analysis. The process began with system energy optimization to achieve the system’s minimum energy state. NVT phylogenetic simulation of 500 ps at 298 K was performed to ensure a uniform distribution of solvent molecules within the solvent box. Under periodic boundary conditions, a 100 ns NPT simulation was conducted to understand the behavioral dynamics of the HSA–PFAS complexes under simulated biological conditions. Other process conditions were set as follows: a non-bond cutoff distance of 10 Å, PME method for long-range electrostatic interaction calculation [38], SHAKE method for hydrogen bond length constraints [39], and Langevin algorithm for temperature control [40]. During MD simulation, key indicators like root mean square deviation (RMSD) were monitored to track structural changes in HSA–PFAS complexes over time and determine whether the system had reached thermodynamic equilibrium.

The MM/GBSA method combines molecular mechanics energy components (MM) with the implicit solvent model (GBSA) to determine the binding free energy of HSA–PFAS binding [41,42,43], as shown in Equation (2):

$$\Delta G_{\mathrm{bind}}=\Delta G_{\mathrm{complex}}-(\Delta G_{\mathrm{HSA}}+\Delta G_{\mathrm{PFAS}})=\Delta E_{\mathrm{VDW}}+\Delta E_{\mathrm{ELE}}+\Delta G_{\mathrm{GB}}+\Delta G_{\mathrm{SA}}$$

ΔG_complex_, ΔG_HSA_, and ΔG_PFAS_ indicate the free energy of complex, HSA, and PFAS, respectively. ΔE_VDW_, ΔE_ELE_, ΔG_GB_, and ΔG_SA_ refer to van der Waals, electrostatic, polar solvation and non-polar solvation-free energy [44]. ΔG_GB_ was calculated using the GB model [45]. ΔG_SA_ was also determined to reflect the interaction of the molecular surface’s non-polar portions with the solvent [46].

## 3. Results and Discussion

### 3.1. Fluorescence Quenching Mechanism

**Figure 1:**
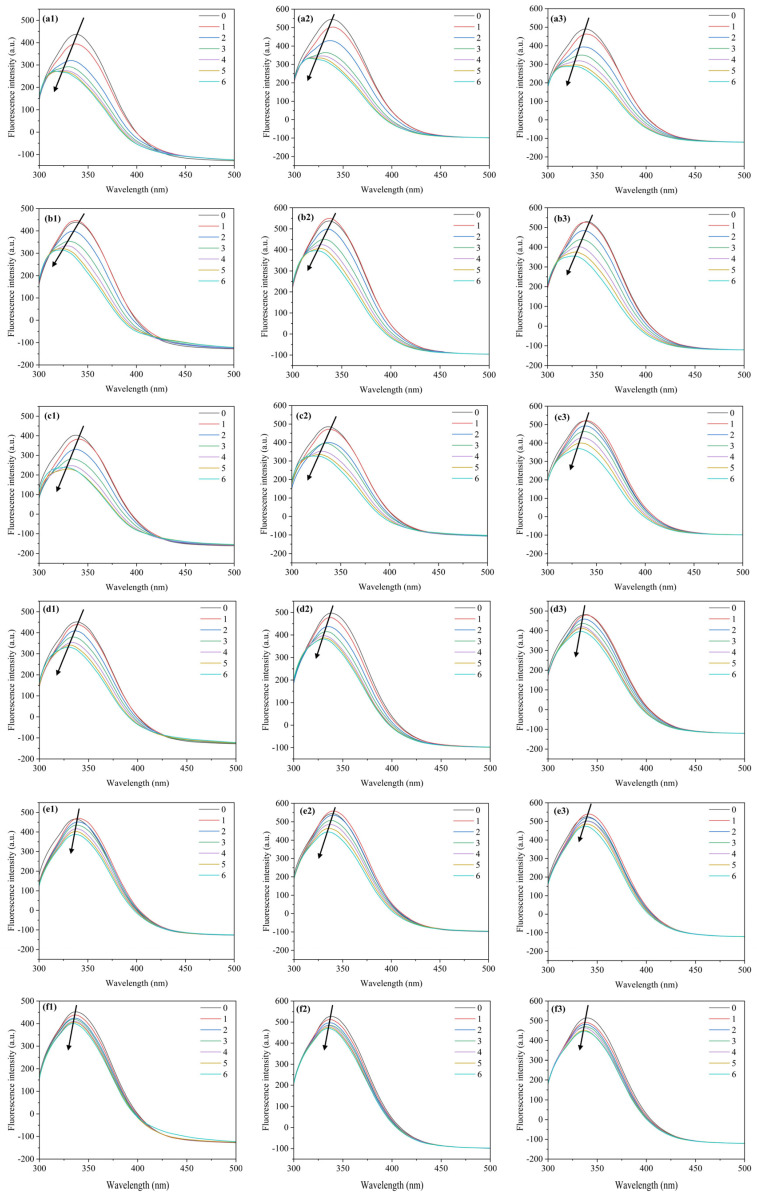
Fluorescence spectra of the HSA–PFAS system at different temperatures. C_[HSA]_ = 1 × 10^−6^ mol·L^−1^, C_[PFAS]_ = 0, 3, 6, 9, 12, 15 and 18 × 10^−6^ mol·L^−1^, T = 298 K, 304 K, 310 K. (**a1**) HSA–PFNA–298 K (**a2**) HSA–PFNA–304 K (**a3**) HSA–PFNA–310 K (**b1**) HSA–HFPO-TA–298 K (**b2**) HSA–HFPO-TA–304 K (**b3**) HSA–HFPO-TA–310 K (**c1**) HSA–PFOA–298 K (**c2**) HSA–PFOA–304 K (**c3**) HSA–PFOA–310 K (**d1**) HSA–PFO3DA–298 K (**d2**) HSA–PFO3DA–304 K (**d3**) HSA–PFO3DA–310 K (**e1**) HSA–PFHpA–298 K (**e2**) HSA–PFHpA–304 K (**e3**) HSA–PFHpA–310 K (**f1**) HSA–DFSA–298 K (**f2**) HSA–DFSA–304 K (**f3**) HSA–DFSA–310 K.

Figure 1 exhibits changes in the HSA spectrum with the continuous addition of PFAS. The fluorescence peak of HSA is located at 337 nm. The fluorescence intensity gradually decreases with PFAS concentration at 298 K, 304 K, 310 K, indicating the formation of complexes between PFAS and HSA [47]. Among the six PFAS, PFNA has the greatest effect on the fluorescence intensity. PFNA causes a 30.6% quenching of fluorescence intensity at a concentration of 1.8 × 10^−5^ mol·L^−1^, while HFPO-TA, PFOA, PFO3DA, PFHpA, and DFSA cause fluorescence quenching rates of 25.1%, 20.1%, 15.3%, 12.1%, and 9.7% at 298 K, respectively. This phenomenon suggests that PFNA has the greatest influence on HSA.

Furthermore, Figure 1 shows that all six PFAS cause a blue shift in the fluorescence peak of HSA, indicating that PFAS have altered the polarity of the microenvironment near amino acid residues. The blue shift caused by the binding of three PFAS (PFNA, HFPO-TA, and PFOA) to HSA is the most significant compared to the others. The fluorescence peak shifts from 337 nm to 317 nm (PFNA), 315 nm (HFPO-TA), and 320 nm (PFOA) as the concentration of PFAS increases, indicating that they may have a greater influence on microenvironment hydrophobicity in HSA.

Fluorescence quenching is usually caused by a series of complex processes. Dynamic quenching, static quenching, and mixed-type quenching are the three types of quenching processes [48]. Static quenching is primarily manifested by organic small molecules forming ground state complexes with proteins via intermolecular forces, whereas dynamic quenching is typically associated with the collision between fluorescent groups and quenchers. Dynamic quenching depends on molecular diffusion, and its quenching constant increases with the rising temperature; however, static quenching is due to the fact that high temperatures promote the dissociation of complexes, resulting in a decrease in quenching constants [3]. The quenching constant can be calculated using the Stern–Volmer Equation (3). The results are shown in Table 2 and Figure 1:

$$F_{0}/F=1+K_{q}\tau_{0}=1+K_{sv}\left[Q\right]$$

where *F*_0_ and *F* refer to HSA fluorescence intensities without and with the quencher (PFAS solution); *K_q_* is the biomacromolecule’s quenching rate constant; *τ*_0_ is the average fluorescence lifetime of the fluorescent molecule when the quencher PFAS is absent, usually taken as 10^−8^ s; [*Q*] is the PFAS concentration; *K_sv_* is the Stern–Volmer quenching constant; *F*_0_*/F* is the vertical axis; the Stern–Volmer curves of this system are 298, 304, and 310 K.

The *K_sv_* values decrease with temperature for HSA–PFNA, HSA–PFO3DA, HSA–PFHpA, and HSA–DFSA binding (Table 1), revealing that the quenching mechanism is primarily static. Furthermore, *K*_q_ values at 298 K range from 5.83 × 10^11^ to 2.50 × 10^12^ L·mol^−1^·s^−1^, which are much larger than the maximum dynamic diffusion quenching constant of the fluorescent agent for the fluorescent molecule (2.0 × 10^10^ L·mol^−1^·s^−1^). As a result, PFNA, PFO3DA, PFHpA, and DFSA can easily quench fluorescence groups by generating a complex, resulting in a static quenching process.

Furthermore, for the HSA–PFOA and HSA–HFPO-TA binding systems, *K*_sv_ values increase with temperature, implying a dynamic quenching process. However, at 298 K, the *K*_q_ values are 1.78 × 10^12^ L·mol^−1^·s^−1^ (HFPO-TA) and 1.39× 10^12^ L·mol^−1^·s^−1^ (PFOA). Both of the *K*_q_ values are greater than maximum dynamic diffusion quenching constant, implying the presence of a static quenching mechanism. Therefore, the fluorescence quenching mechanism of PFOA and HFPO-TA on HSA is a mixed quenching process that combines dynamic and static mechanisms.

### 3.2. Binding Constant and the Numbers of Binding Sites

**Figure A2:**
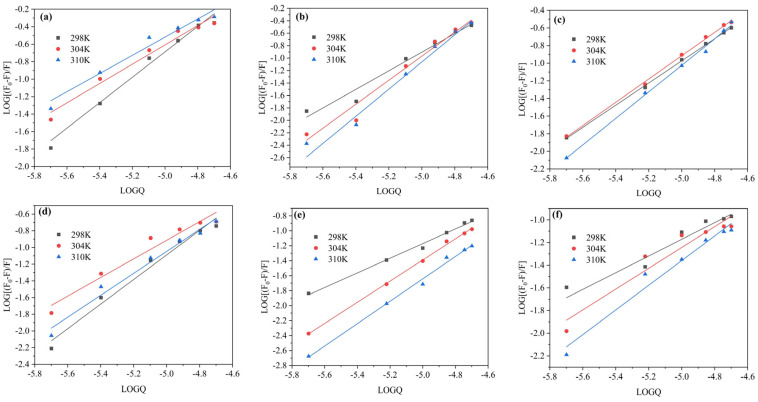
Double-logarithm plots of HSA–PFAS system at different temperatures. C_[HSA]_ = 1 × 10^−6^ mol·L^−1^, C_[PFAS]_ = 0, 3, 6, 9, 12, 15, and 18 × 10^−6^ mol·L^−1^, T = 298 K, 304 K, 310 K. (**a**) HSA–PFNA (**b**) HSA–HFPO-TA (**c**) HSA–PFOA (**d**) HSA–PFO3DA (**e**) HSA–PFHpA (**f**) HSA–DFSA.

The double logarithmic formula can be used to calculate the binding constants and binding site numbers of HSA–PFAS complexes [49]:

$$\log\left[\left(F_{0}-F\right)/F\right]=\log K_{b}+n\log\left[Q\right]$$

where *F*_0_ and *F* are parameters representing the initial fluorescence intensity and the fluorescence intensity after adding PFAS, respectively. The binding characteristics are represented by the binding constant, *K*_b_, and the number of binding sites, *n*. The slope of the straight line is the number of binding sites, *n*, and the binding constant *K*_b_ is obtained from the exponent of the straight line’s intercept using the double logarithmic graph. Figure A2 and Table 2 show the calculation results.

In theory, the number of binding sites should be an integer because each represents a unique binding site on the protein. In practice, however, the value of “*n*” is typically derived by fitting binding models to the experimental data, which yields non-integer values. Table 2 shows that the *n* values range from 0.8757 to 2.0857, indicating that PFAS bind on over one site of HSA. Except for the HSA–HFPO-TA binding at 310 K (*n* = 2.09, all the derived binding constants are close to one, indicating the presence of a single binding site on the HSA–PFAS complex. The binding constants *K*_b_ values of the six PFAS at 298 K range from 1.52 × 10^3^ to 7.81 × 10^6^ L·mol^−1^. The binding constants are listed in the following order: PFNA (7.81 × 10^6^ L·mol^−1^) > HFPO-TA (3.70 × 10^6^ L·mol^−1^) > PFOA (2.27 × 10^5^ L·mol^−1^) > PFO3DA (1.59 × 10^5^ L·mol^−1^) > PFHpA (4.53 × 10^3^ L·mol^−1^) > DFSA (1.52 × 10^3^ L·mol^−1^), with PFNA having the largest binding constant. PFNA, PFOA, and PFHpA are structurally similar perfluoroalkyl carboxylic compounds with carbon chain lengths in the order PFNA (C9) > PFOA (C8) > PFHpA (C7). The binding constants of these three PFAS are positively correlated with their carbon chain lengths, i.e., the longer the carbon chain, the larger the binding constant. The results of the above analysis show that increasing the carbon chain length significantly increases the binding affinity of HSA–PFAS, which is consistent with previous research findings [7].

Furthermore, the *K*_b_ values for the HSA–PFHpA and HSA–DFSA binding systems (4.53 × 10^3^ L·mol^−1^, 1.52 × 10^3^ L·mol^−1^) are much lower than 10^5^ L·mol^−1^. The *K*_b_ of PFHpA, DFSA and HSA is weaker than that of other PFAS. The lower binding constant increases the concentration of free PFHpA and DFSA in the blood system, slowing their metabolic process in the body and potentially increasing their toxicity to the biological blood system [5].

### 3.3. Thermodynamic Analysis of the Binding Process

The enthalpy change (∆*H*), entropy change (∆*S*), and free energy change (∆*G*) calculated from the Van’t Hoff equation [50] can be used to determine the type of interaction.

$$\ln K_{b}=-\Delta H/RT+\Delta S/R$$

$$\Delta G=\Delta H-T\Delta S=-RT\ln K_{b}$$

where *R* represents the ideal gas constant (8.314 J·mol^−1^·K^−1^).

When both ∆*H* and ∆*S* are positive, they indicate the interaction force of the hydrophobic interaction. When they are both negative, they indicate the interaction forces of hydrogen bonds or van der Waals forces. When ∆*H* is close to 0, and especially when it is less than 0, and ∆*S* is greater than 0, electrostatic interaction may be the dominant interaction force [12].

According to the results in Table 2, ∆*H* and ∆*S* for HSA–PFNA and HSA–PFO3DA binding are both negative, indicating the presence of hydrogen bonds or van der Waals forces. For the other four bindings (HSA–HFPO-TA, HSA–PFOA, HSA–PFHpA, and HSA–DFSA), both ∆*H* (146.68–412.15 kJ·mol^−1^) and ∆*S* (593.3–1508.3 J·mol^−1^·K^−1^) are positive, indicating the presence of hydrophobic interactions. Hydrogen bonding and hydrophobic interactions are two major types of molecular interactions that frequently coexist and influence molecular binding behavior. With its longer nine-carbon chain, PFNA may increase the van der Waals contact area with proteins, facilitating hydrogen bond formation at specific sites. However, PFOA and PFHpA have shorter carbon chains, with eight and seven carbon atoms, respectively. This shorter length may confer greater flexibility, allowing molecules to fit and embed more easily into the hydrophobic pockets of proteins, enhancing hydrophobic interactions. The ∆*G* results calculated from Equation (6) are all negative, ranging between −39.32 and −18.15 kJ·mol^−1^, indicating that six HSA–PFAS binding is a spontaneous process that is mainly driven by entropy.

### 3.4. Changes in HSA Conformation after Interaction with PFAS

#### 3.4.1. UV-vis Absorption Spectroscopy

**Figure 2:**
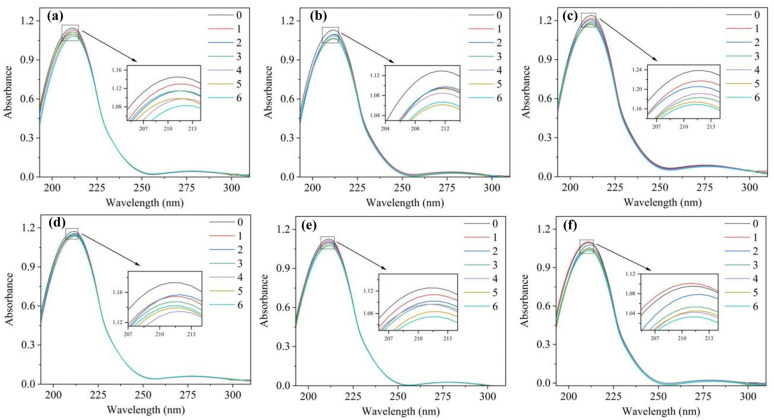
UV-vis absorption spectra of HSA–PFAS. C_[HSA]_ = 1 × 10^−6^ mol·L^−1^, C_[PFAS]_ = 0, 3, 6, 9, 12, 15 and 18 × 10^−6^ mol·L^−1^. (**a**) HSA–PFNA (**b**) HSA–HFPO-TA (**c**) HSA–PFOA (**d**) HSA–PFO3DA (**e**) HSA–PFHpA (**f**) HSA–DFSA.

The UV–vis absorption spectrum is a rapid technique for exploring complex formation and changes in protein conformation [50]. Figure 2 depicts HSA UV-vis spectra with PFAS (0, 3, 6, 9, 12, 15, 18 × 10^−6^ mol·L^−1^) at 298 K. HSA displays a significant absorption peak at 210 nm. With the increase in PFAS concentration, the peak value of absorption gradually decreases, and the maximum absorption wavelength shifts from 210 nm to 213 nm. This phenomenon, known as a red shift, is due to the binding of PFAS and the base pairs of HSA to the π electrons, which reduces the energy and leads to a decrease in the energy of the π→π* transition [18]. An increase in the hydrophobicity and a decrease in the hydrophilicity of residues lead to polarity reduction in the microenvironment of HSA. UV-vis red shift reveals that the presence of PFAS altered the secondary structure of HSA [51].

#### 3.4.2. Synchronous Fluorescence Spectroscopy

**Figure 3:**
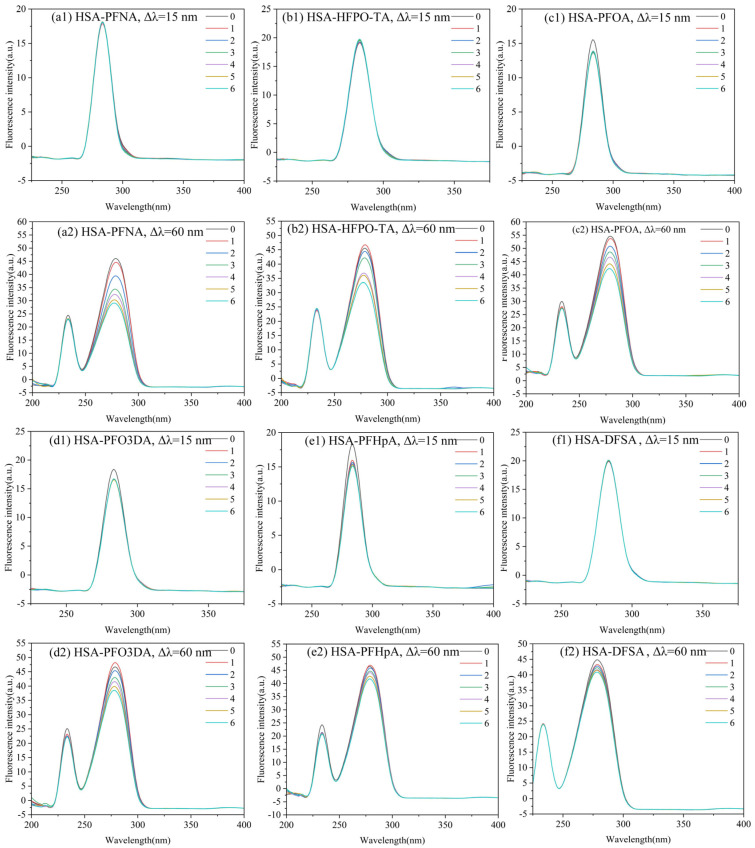
Synchronized fluorescence spectra of HSA–PFAS. C_[HSA]_ = 1 × 10^−6^ mol·L^−1^, C_[PFAS]_ = 0, 3, 6, 9, 12, 15 and 18 × 10^−6^ mol·L^−1^. Δλ_(1)_ = 15 nm, Δλ_(2)_ = 60 nm.

Figure 3 shows that the synchronous fluorescence peak in Δλ = 15 nm, which is associated with tyrosine (Tyr) residues, remains largely unchanged as PFAS concentrations increase. Notably, there is little change in peak shape and only a minor amount of fluorescence quenching for the six HSA–PFAS complexes, in the range of 3.2–17.0%.

However, significant decreases were observed for the synchronous fluorescence peak of Δλ = 60 nm, regarding tryptophan (Trp) residues [52]. In this case, a 3.0 nm red shift in the fluorescence peak of Δλ = 60 nm is observed. This shift is accompanied by significant fluorescence quenching, as indicated by a decrease in the range of 16.1–37.0%. These changes indicate that PFAS increases its polarity around tryptophan (Trp) residues in HSA. As a result, their hydrophobicity is reduced, and HSA undergoes some conformational changes [52].

#### 3.4.3. Three-Dimensional Fluorescence Spectra

**Figure 4:**
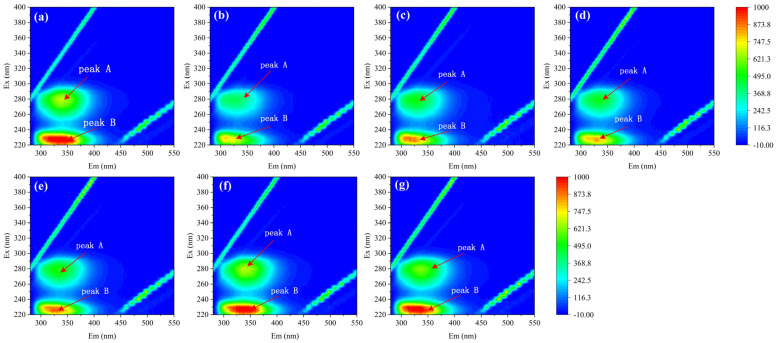
The 3D-EEM spectra of HSA–PFAS. C_[HSA]_ = 1 × 10^−6^ mol·L^−1^, C_[PFAS]_ = 1.8 × 10^−5^ mol·L^−1^. (**a**) HSA (**b**) HSA–PFNA (**c**) HSA–HFPO-TA (**d**) HSA–PFOA (**e**) HSA–PFO3DA (**f**) HSA–PFHpA (**g**) HSA–DFSA.

Figure 4 depicts the 3D-EEM contour plots of the HSA–PFAS complex. Peak A (λ_ex_/λ_em_ = 280/337 nm) and peak B (λ_ex_/λ_em_ = 230/340 nm) refer to the characteristics of amino acid residues. Take PFNA, for example: peak A’s intensity was reduced by 37.8% after binding with HSA, while peak B’s intensity was reduced by 19.7%. The decrease in fluorescence intensity suggests that PFAS cause the partial unfolding of the HSA polypeptide chains, converting the initially hydrophobic regions to hydrophilic and initiating conformational changes within HSA [53].

With the addition of PFAS, the positions of peak A and peak B are also shifted. In the HSA–PFAS mixed system, peak A transitioned from λ_ex_/λ_em_ = 280/337 nm to 280/331 nm, and peak B from λ_ex_/λ_em_ = 230/340 nm to 230/314 nm, leading to a blue shift. The presence of PFAS disrupts the molecular surface of HSA, causing depolymerization and reduced protein size. This leads to weaker fluorescence, indicating changes in HSA’s secondary structure.

#### 3.4.4. Circular Dichroism (CD) Spectral Analysis

**Figure 5:**
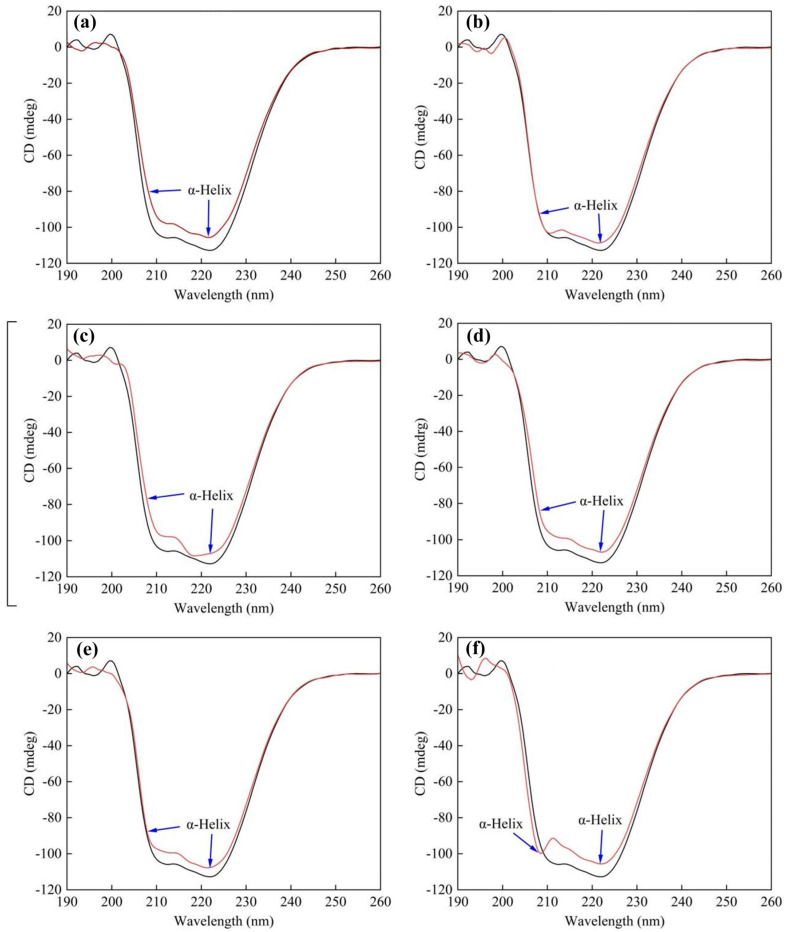
The circular dichroism spectra of HSA interacting with six PFAS. C_[HSA]_ = 1 × 10^−6^ mol·L^−1^, C_[PFAS]_ = 18 × 10^−6^ mol·L^−1^, T = 298 K, pH = 7.4. (**a**) HSA–PFNA (**b**) HSA–HFPO-TA (**c**) HSA–PFOA (**d**) HSA–PFO3DA (**e**) HSA–PFHpA (**f**) HSA–DFSA.

Figure 5 demonstrates that HSA exhibits two prominent negative absorption bands at 208 nm and 222 nm, which are closely related to its α-helix structure [54]. HSA’s secondary structure is made up of 41.6% α-helix, 4.9% β-sheet, 16.9% β-turn, and 36.7% random coil. Changes in HSA’s secondary structure were observed with the addition of 18 × 10^−6^ mol/L PFAS, which manifested as a decrease in α-helix content and an increase in β-fold, β-turn, and random coil contents, except for DFSA. This may be due to the smallest binding constant occurring in HSA–DFSA, and the binding of DFSA to some extent stabilizes the α-helix structure of HSA. PFNA had the greatest influence on the CD spectrum of HSA. The α-helix content decreased from 41.6% to 36.2% when PFNA was added to the HSA solution, while the β-sheet content increased from 4.9% to 7.6%. Following that, the two compounds PFHpA and PFOA also reduced the α-helix content to 36.4% and 37.2%, respectively. The α-helix is usually formed by twisting and folding the polypeptide chain. The introduced PFAS interact with HSA, disrupting its hydrogen bonding and loosening the peptide chains [55]. As a result, HSA–PFAS binding leads to alterations in the protein’s secondary structure.

### 3.5. Competition Binding of PFAS with HSA

In the presence of three probe substances (warfarin, ibuprofen, and lidocaine), the binding constants of the ternary system exhibit varying degrees of decrease, as calculated using Equation (7), and listed in Table 3.

$$\varphi =\frac{K_{b^{\prime }}-K_{b}}{K_{b^{\prime }}}\times 10$$

where *K_b_* and *K_b’_* are the binding constants of the HSA–PFAS complex with and without the probe, respectively.

Table 3 shows that the three competing probe substances have different effects on HSA–PFAS binding. The *K*_b_ values of six PFAS decreased in the presence of ibuprofen (subdomain IIIA) by 13.8–42.7%, whereas lidocaine (subdomain IB) decreased by 8.1–48.8%. This suggests that the effect of ibuprofen and lidocaine on HSA–PFAS binding is limited. There was no competitive binding between ibuprofen/lidocaine and PFAS. The decreases in the HSA–PFAS binding constants are primarily due to micro-structural changes in HSA after binding with ibuprofen or lidocaine, which further affect HSA–PFAS binding.

However, the presence of warfarin probe (subdomain IIA) leads to a significant reduction in the binding constants of HSA–PFAS complexes, with the value of φ ranging from 93.3% to 99.6%. The binding of HFPO-TA, in particular, showed a decrease in the *K*_b_ value of 99.6% from 2.70 × 10^6^ to 1.48 × 10^4^. This suggests that the binding region of the HSA–PFAS complex is primarily in subdomain IIA of HSA.

When warfarin is already bound to subdomain IIA of HSA, it creates a competitive environment for PFAS. Since warfarin occupies the subdomain IIA, PFAS are hindered or inhibited from binding to this same site, leading to a reduced binding affinity for PFAS on HSA. The conclusion was later validated by the molecular docking results. PFAS are frequently found in mixtures in environmental and biological systems. The primary focus of this research is on the binding properties of single PFAS with HSA, but understanding the competitive binding of mixed PFAS is also important. Different PFAS may compete for the same binding sites on HSA in mixtures. This type of competition can have an impact on the binding affinity and stability of each PFAS. Existing research [56] also revealed that multiple drugs in a mixture may exhibit synergistic binding behaviors in complex drug–protein systems, significantly enhancing the bioactivity and toxicological properties of individual drugs. Future research will explore various PFAS mixtures to gain a better understanding of their binding dynamics with HSA, providing an improved understanding of PFAS interactions.

### 3.6. Quantum Chemistry Structural Analysis of PFAS

#### 3.6.1. Frontier Molecular Orbital (FMO) Analysis

**Figure 6:**
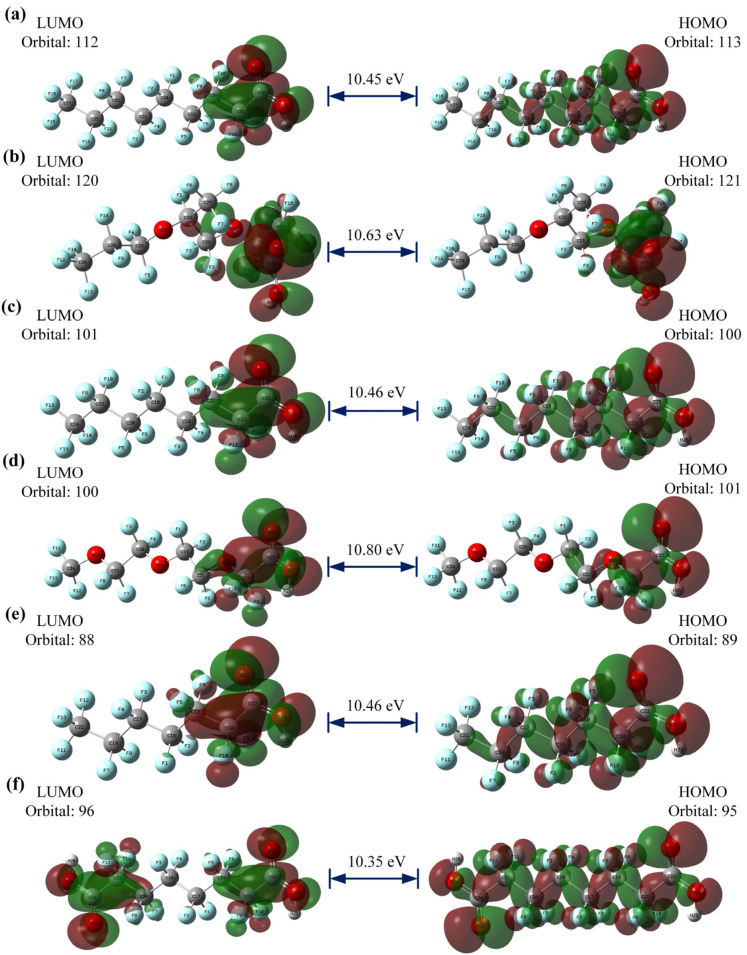
The HOMO and LUMO orbitals of six PFAS. (**a**) PFNA (**b**) HFPO-TA (**c**) PFOA (**d**) PFO3DA (**e**) PFHpA (**f**) DFSA.

HOMO and LUMO are the important descriptors that influence the electrical and optical properties of compounds. The HOMO often serves as an electron contributor, while the LUMO often acts as an electron acceptor in chemical reactions, as shown in Figure 6. Table A3 also shows the molecular structures of six PFAS, as well as their HOMO and LUMO electron densities and radical electron densities. Most of the electron densities of these six PFAS in orbitals are clearly observed on the carbonyl oxygen in the carboxyl (-COOH) group, revealing that the carboxyl group can undergo radical reactions. The charge of the HOMO orbital is primarily located on the carboxyl (-COOH) groups, the oxygen atoms connected to carboxyl groups, and the carbon–fluorine (C-F) bond. The orbital distribution of DFSA is obviously different from that of the other five PFAS due to the presence of two carboxyl groups in DFSA, as shown in Figure 6.

The energy gap (named ∆E_HOMO-LUMO_) can provide insights into a molecule’s stability, reactivity, and even some of its photophysical properties [57]. The ∆E_HOMO-LUMO_ values of the six PFAS range from 0.3802 eV to 0.3970 eV, implying that these molecules are conducive to chemical reactions.

#### 3.6.2. Molecular Surface Properties Approach (MSPA) Analysis

The MSPA technique is a powerful tool for analyzing molecular surface attributes such as the electrostatic potential (ESP) and average localized ionization energy (ALIE). These descriptors can depict the entire charge distribution [58], potentially aiding in a better understanding of the molecule’s chemical reactivity.

**Figure 7:**
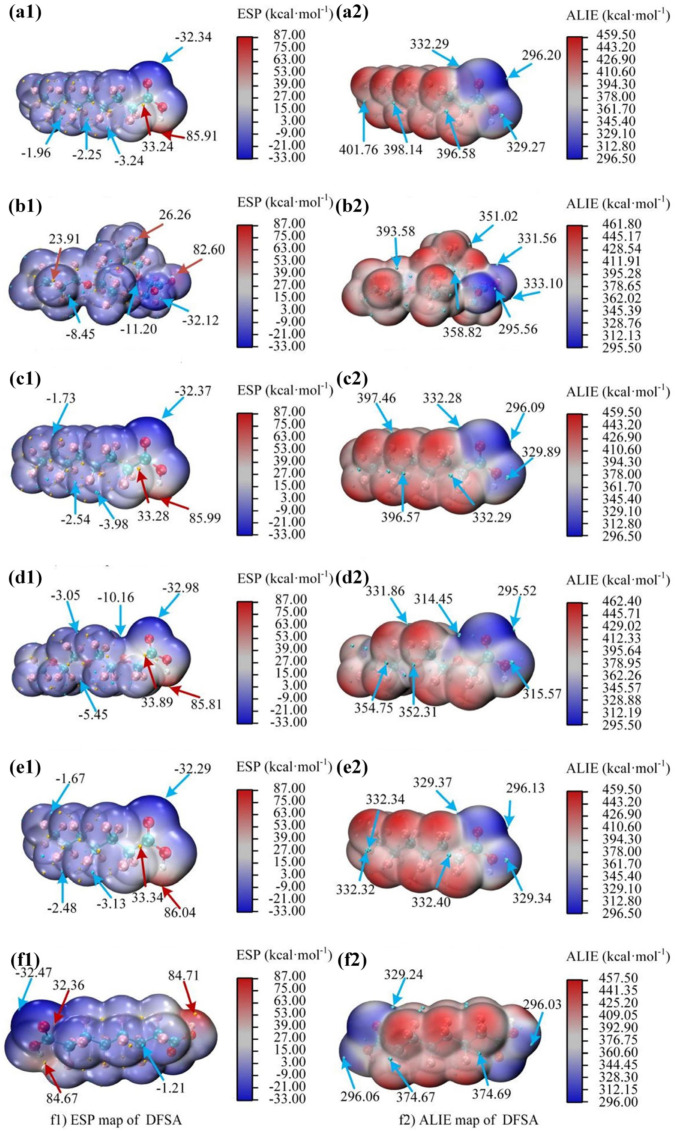
ESP and ALIE maps of six PFAS. (**a1**) ESP map of PFNA (**a2**) ALIE map of PFNA (**b1**) ESP map of HFPO-TA (**b2**) ALIE map of HFPO-TA (**c1**) ESP map of PFOA (**c2**) ALIE map of PFOA (**d1**) ESP map of PFO3DA (**d2**) ALIE map of PFO3DA (**e1**) ESP map of PFHpA (**e2**) ALIE map of PFHpA (**f1**) ESP map of DFSA (**f2**) ALIE map of DFSA.

As shown in Figure 7 and Table A5, Table A6, Table A7, Table A8, Table A9, Table A10, Table A11, Table A12, Table A13, Table A14, Table A15 and Table A16, the total electron density profile is represented by a color gradient, making it easier to identify the most active sites for nucleophiles and electrophiles [26]. Electrophilic reactions are more likely to occur in regions with a higher negative electrostatic potential. The local minima for six PFAS were notably located proximal to the oxygen atom in six PFAS, with values of −32.34 kcal/mol (PFNA), −32.12 kcal/mol (HFPO-TA), −32.37 kcal/mol (PFOA), −32.98 kcal/mol (PFO3DA), −32.29 kcal/mol (PFHpA), and −32.47 kcal/mol (DFSA). This implies that electrophilic reagents can easily target the oxygen atom, highlighting its strong electro-positive character, resulting in an increase in the reactive activity at these sites in PFAS.

ALIE is an index for electron localization in molecules, which is used to identify electrophilic sites as an effective complement to ESP [26,59]. The blue region in the ALIE maps of PFAS, as shown in Figure 7 and Table A17, Table A18, Table A19, Table A20, Table A21, Table A22, Table A23, Table A24, Table A25, Table A26, Table A27 and Table A28, primarily hovers around the carboxyl group and its neighboring oxygen atoms. Using PFHpA as an example, the deepest blue was clearly seen adjacent to the O atom in the -COO group, representing the local minimum value of 295.52 kcal/mol. This indicates that the electron activity near the oxygen atom is stronger, making it more prone to undergoing electrophilic reactions.

#### 3.6.3. Conceptual Density Functional Theory (CDFT) Analysis

**Figure 8:**
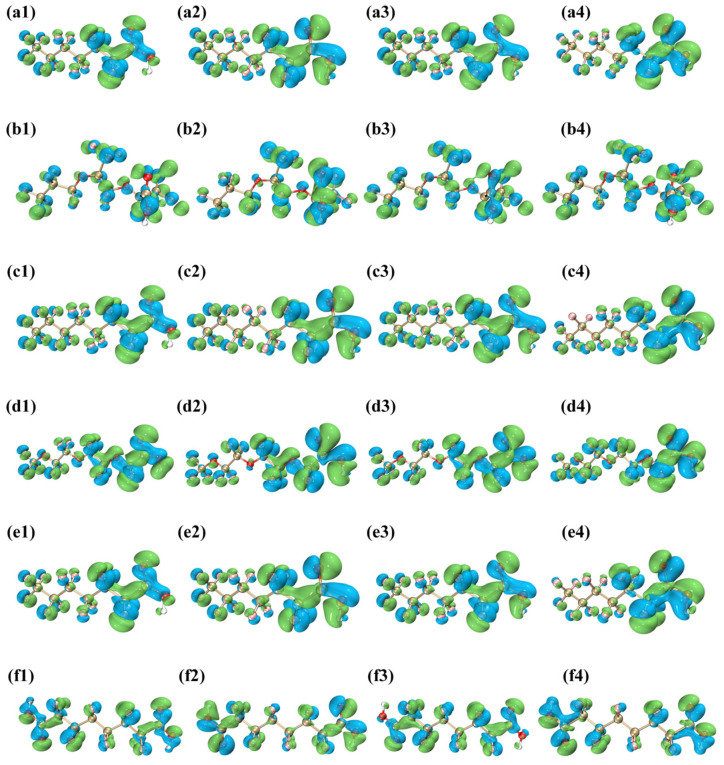
Visualization of CDFT descriptors: (1) nucleophilic Fukui function *f*^+^_(r)_, (2) electrophilic Fukui function *f*^−^_(r)_, (3) free radical Fukui function *f*^0^_(r)_, and (4) condensed dual descriptors Δ*f*_(r)_ of PFAS. (**a1**) *f*⁺₍ᵣ₎ of PFNA (**a2**) *f*^−^₍ᵣ₎ of PFNA (**a3**) *f*^0^₍ᵣ₎ of PFNA (**a4**) ∆*f*₍ᵣ₎ of PFNA (**b1**) *f*⁺₍ᵣ₎ of HFPO-TA (**b2**) *f*^−^₍ᵣ₎ of HFPO-TA (**b3**) *f*^0^₍ᵣ₎ of HFPO-TA (**b4**) ∆*f*₍ᵣ₎ of HFPO-TA (**c1**) *f*⁺₍ᵣ₎ of PFOA (**c2**) *f*^−^₍ᵣ₎ of PFOA (**c3**) *f*^0^₍ᵣ₎ of PFOA (**c4**) ∆*f*₍ᵣ₎ of PFOA (**d1**) *f*⁺₍ᵣ₎ of PFO_3_DA (**d2**) *f*^−^₍ᵣ₎ of PFO_3_DA (**d3**) *f*^0^₍ᵣ₎ of PFO_3_DA (**d4**) ∆*f*₍ᵣ₎ of PFO_3_DA (**e1**) *f*⁺₍ᵣ₎ of PFHpA (**e2**) *f*^−^₍ᵣ₎ of PFHpA (**e3**) *f*^0^₍ᵣ₎ of PFHpA (**e4**) ∆*f*₍ᵣ₎ of PFHpA (**f1**) *f*⁺₍ᵣ₎ of DFSA (**f2**) *f*^−^₍ᵣ₎ of DFSA (**f3**) *f*^0^₍ᵣ₎ of DFSA (**f4**) ∆*f*₍ᵣ₎ of DFSA.

CDFT, grounded in the study of electronic density, offers comprehensive qualitative and quantitative insights into the chemical reactivity of molecular systems. Typical CDFT descriptors, such as Fukui function and dual descriptor (DD, Δ*f*_(r)_), can reveal the regions of molecules that are most vulnerable to electrophilic or nucleophilic attacks [60]. The results are shown in Figure 8 and Table A29, Table A30, Table A31, Table A32, Table A33 and Table A34.

Higher Fukui function values for a given site often indicate an increased sensitivity to electrophilic attacks [61]. Notably, the DD index outperforms the Fukui function alone in predicting both electrophilic and nucleophilic reactive sites. That is, positive DD values indicate nucleophilic attack potential, whereas negative values represent electrophilic attack potential [62].

Using PFNA as an example, the Δ*f*_(r)_ value for the C28 atom in the carboxyl group is 0.0764. This not only highlights its extreme sensitivity to electrophilic attacks, but also its critical role in PFNA’s overall reactivity and the carboxyl group’s importance in electrophilic reactions. Furthermore, F13 and F14 in the PFNA structure both have the same significant Δ*f*_(r)_ values of 0.0371. The other five PFAS have the same distribution and nature of potential reactive sites as PFNA. This suggests that the structure–reactivity patterns of these compounds may be similar.

The calculated global reactivity descriptors of the PFAS are also listed in Table A29, Table A30, Table A31, Table A32, Table A33 and Table A34. As shown in Table A29, the chemical hardness (η) for PFNA is 6.8335 eV, while its chemical softness (s) is 0.1463 eV^−1^; these can be interpreted as indicators of intra-molecular charge transfer characteristics. The high hardness (η) and low softness (s) reveal that PFNA is a soft molecule. PFNA also has an electrophilicity index (ω) of 2.4383 eV, which classifies it as a “strong electrophile” (>1.50 eV) according to the organic classification criteria [57]. The electronegativity (χ) of PFNA is 5.7727 eV, a descriptor that quantifies an atom or molecular group’s ability to attract electrons.

#### 3.6.4. Electron Localization Characteristic Analysis

**Figure 9:**
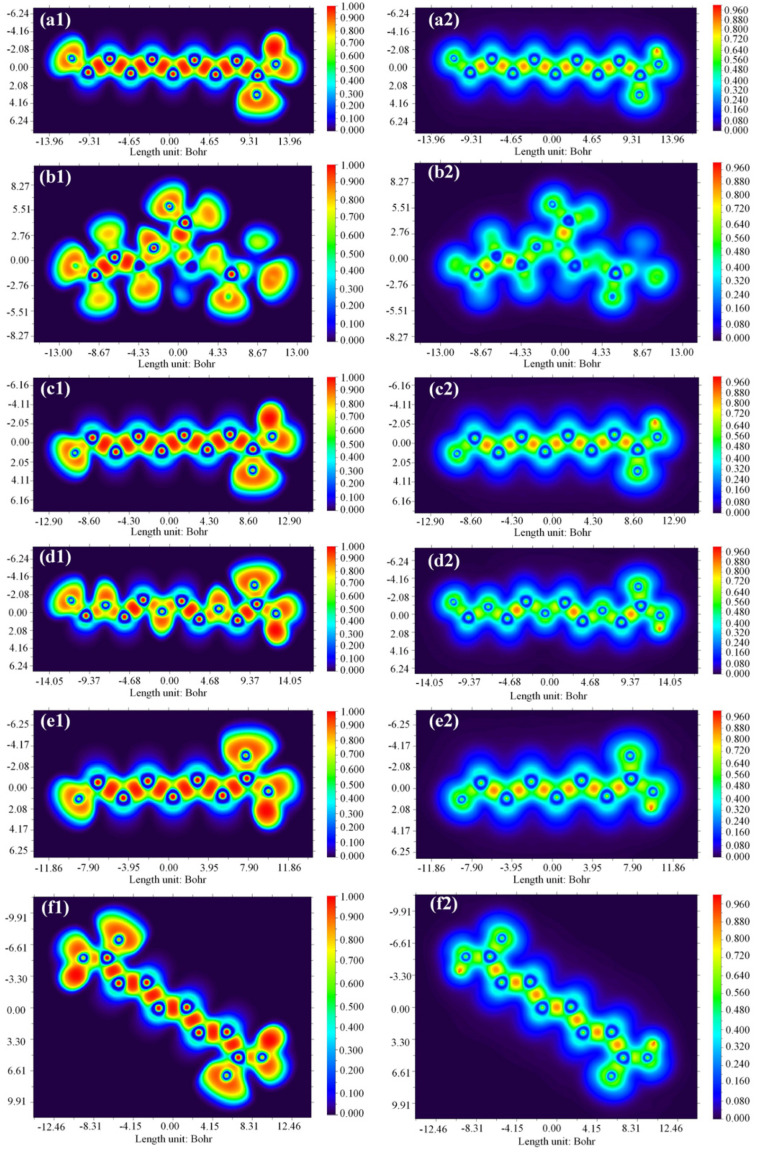
ELF and LOL diagram of six PFAS. (**a1**) ELF map of PFNA (**a2**) LOL map of PFNA (**b1**) ELF map of HFPO-TA (**b2**) LOL map of HFPO-TA (**c1**) ELF map of PFOA (**c2**) LOL map of PFOA (**d1**) ELF map of PFO3DA (**d2**) LOL map of PFO3DA (**e1**) ELF map of PFHpA (**e2**) LOL map of PFHpA (**f1**) ELF map of DFSA (**f2**) LOL map of DFSA.

The Electron Localization Function (ELF) and Localized Orbital Locator (LOL) serve as tools for delineating the electron localization characteristics of molecules. ELF is often used to examine the nature of chemical bonds and to identify electron distribution, while LOL is commonly used to identify electron orbitals like non-bonding and lone pairs [63]. The topological features of six PFAS were analyzed using MultiWFN software. Figure 9 depicts the ELF and LOL contour projections for these molecules, with a gradient from blue to red representing ELF and LOL values ranging from 0 to 1. Values between 0.5 and 1 represent localized bonding and non-bonding electrons, while values less than 0.5 represent delocalized electrons [64]. The LOL plot offers similar information to ELF, but might be more sensitive to electron delocalization features.

Areas around the C, F, and O atoms are highlighted in blue in the ELF plots of the six PFAS (Figure 9), indicating the presence of low ELF values (<0.5) and electron delocalization. On the other hand, areas surrounding the H atoms are depicted in rich reds with high ELF values, indicating a strong localization of both bonding and non-bonding electrons.

#### 3.6.5. Interaction Region Indicator (IRI) Analysis

**Figure 10:**
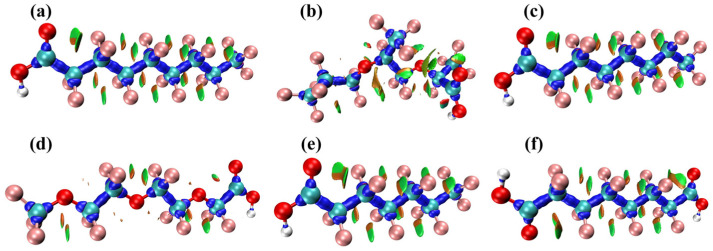
IRI diagram of six PFAS. (**a**) PFNA (**b**) HFPO-TA (**c**) PFOA (**d**) PFO3DA (**e**) PFHpA (**f**) DFSA.

IRI analysis is a novel tool that can identify and reflect various interactions in chemical systems, particularly weak interactions [65]. Figure 10 shows the IRI isosurfaces of six PFAS, with blue representing a notable attraction of H-bond or chemical bonds, green representing van der Waals interactions, and orange and red representing notable repulsion, such as the steric hindrance effect [66].

Taking PFNA as an example, the Van der Waals interaction and steric effect (green and orange) are visible near the F and O atoms in the PFNA molecule (Figure 10a). The orange color near one end of the C–C bonds indicates steric hindrance. Furthermore, the isosurfaces near the O atoms are significantly larger than those near the F atoms, implying that the O atoms have stronger van der Waals interactions and steric hindrance. The other five PFAS have similar structural features.

### 3.7. Analysis of Molecular Docking

**Figure 11:**
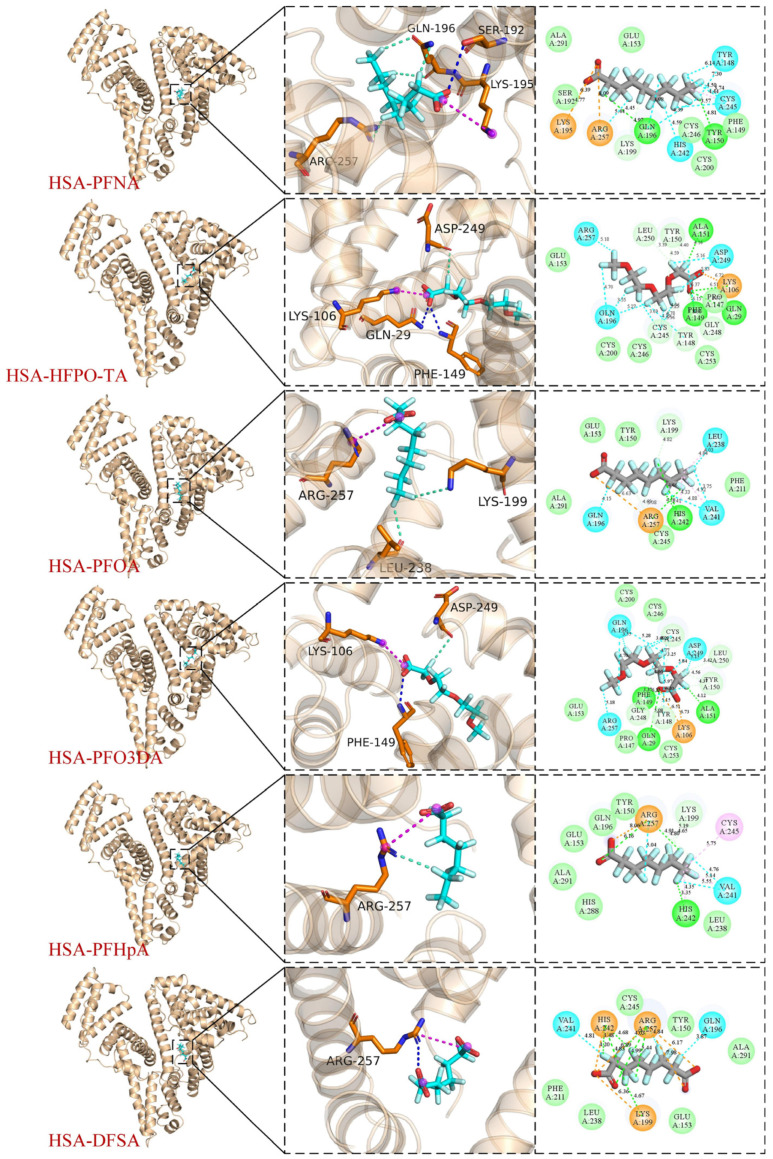
Binding modes of the HSA–PFAS interaction predicted by molecular docking.

Molecular docking techniques were used to explore the binding characteristics of HSA–PFAS binding with the best conformation of HSA–PFAS complexes (Figure 11). The amino acid residues that significantly impact the binding are additionally listed near the PFAS binding sites in Figure 11.

As shown in Figure 11, the binding sites of the six PFAS with HSA are all located in subdomain IIA of HSA, a region known to have a high affinity for various small molecule ligands. This agrees with the findings of the competitive probe experiments (Section 3.3), providing additional support and validation for the molecular docking results. Table A4 also contains detailed docking results.

According to the results of molecular docking (Figure 11), PFAS bind to various amino acid residues on HSA through hydrogen bonds, van der Waals forces, and halogen bonds. In the case of PFNA–HSA binding, the polar end of the PFNA carboxyl group forms a hydrogen bond with the protein’s SER-192 residue, which is critical for the ligand–protein complex’s stability. This is consistent with the thermodynamic results indicating that hydrogen bonding is the primary binding force of PFNA with HSA. Additionally, the Fatom in PFNA is observed to form halogen bonds with positively charged parts of the GLN-196 and ARG-257 residues. Halogen bond is a non-covalent interaction, similar to hydrogen bonds. As the halogen atom of PFOA approaches the nucleophilic site of HSA, it forms a halogen bond, increasing the PFOA’s affinity to and specificity for HSA. PFO3DA docking results are comparable to PFNA.

The other four PFAS (HFPO-TA/PFOA/PFHpA/DFSA) interact with HSA in various ways, including hydrogen bonds, halogen bonds, and hydrophobic interactions. Thermodynamic studies show that the binding forces of these four PFAS with HSA are primarily due to hydrophobic interactions. For example, PFOA, a common perfluorinated compound, is hydrophobic and binds to the non-polar amino acid residue PHE-149, where hydrophobic interactions help to stabilize PFOA in the HSA binding pocket [67].

Molecular docking studies further revealed the binding energies of the six PFAS and HSA. A lower binding energy (more negative) indicates tighter binding between PFAS and HSA. The binding energies of HSA–PFNA, HSA–HFPO-TA, HSA–PFOA, HSA–PFO3DA, HSA–PFHpA, and HSA–DFSA were calculated to be −8.2, −7.9, −7.8, −7.8, −7.1, and −7.3 kcal/mol, respectively. HSA–PFNA has the lowest binding energy of −8.2 kcal/mol, indicating that PFNA more easily binds to HSA. This observation is consistent with the thermodynamic analysis (Section 3.3), which revealed that PFNA exhibits the highest binding constant (7.81 × 10^6^ L·mol^−1^), indicating the strongest affinity between PFNA and HSA. The molecular docking results not only provide an important perspective for understanding the interaction mechanism between PFAS and HSA, but they also provide a scientific foundation for future pollutant removal strategies.

### 3.8. Analysis of MD Simulation Results

The MD simulation is helpful for investigating the complex interactions of small molecules and proteins, revealing the real-time structural dynamics of small molecule–protein complexes under different environmental conditions. The simulation process not only records spatial conformation changes within the complex but also evaluates the dynamic equilibrium and stability of small molecule–protein complexes by calculating dynamic parameters like root mean square deviation (RMSD), the radius of gyration (Rog), root mean square fluctuation (RMSF), and the number of hydrogen bonds.

#### 3.8.1. RMSD

**Figure 12:**
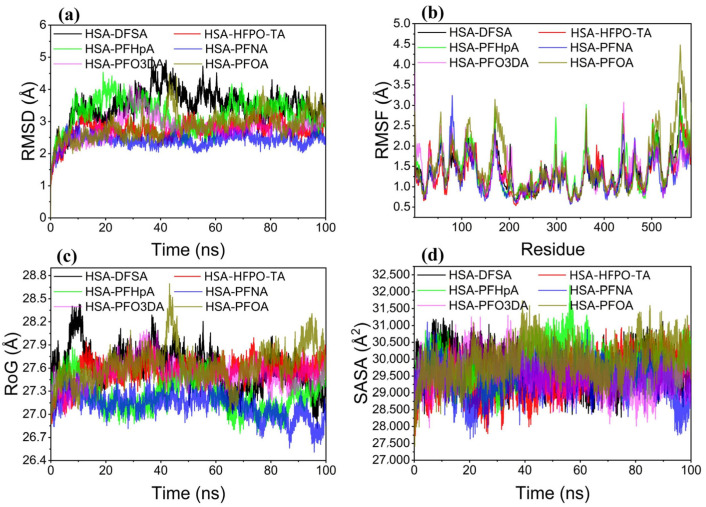
Molecular dynamics simulation of six PFAS and HSA bindings. (**a**) RMSD (**b**) RMSF (**c**) ROG (**d**) SASA.

RMSD is an important indicator for determining whether a system has reached equilibrium, particularly when monitoring displacements of molecular backbone atoms [68]. A larger and more volatile RMSD indicates intense motion. As shown in Figure 12a, the RMSDs of six HSA–PFAS complexes varied between 2 and 4 Å. Among them, HSA–PFHpA and HSA–DFSA complexes have particularly high values and significant fluctuations (over 3.5 Å), indicating a less stable complex binding. HSA–PFNA and HSA–HFPO-TA, on the other hand, have smaller RMSDs (below 3.0 Å) with regular fluctuations during the simulation, indicating a more stable complex formation. All systems show stabilized fluctuations and a gradual reduction after 50 ns, indicating a transition to a new equilibrium state.

#### 3.8.2. RMSF

RMSF reflects protein molecule flexibility during molecular dynamics simulations. Binding with small molecules typically reduces protein flexibility, resulting in protein structure stabilization [69]. Figure 12b shows that after binding with various PFAS, most regions of HSA, except the ends and some local areas, have an RMSF of less than 2.5 Å, indicating a relatively rigid core protein structure. The HSA protein exhibits even lower RMSFs (below 2.0 Å) when bound with PFNA and HFPO-TA, implying that these two small molecules can suppress the protein’s active states, potentially affecting protein function. In contrast, when PFHpA and PFOA bind, HSA exhibits higher RMSFs (above 2.5 Å) in several segments, indicating that these molecules have a less inhibitory effect on the protein.

#### 3.8.3. Rog

Rog reflects the system’s compactness, and monitoring its variations allows for observations of the protein’s folding and unfolding processes [70]. Figure 12c depicts the evolution of Rog over time for six complex systems during MD simulation. All systems have a Rog that ranges between 26.7 Å and 28.5 Å, indicating structural compactness. The PFNA–HSA complex varies between 26.7 Å and 27.3 Å, with the smallest observed Rog values and a downward trend throughout the MD simulation. The low Rog values and minimal fluctuations imply that the system has an increased compactness, which could be attributed to specific interactions between the PFNA molecule and HSA binding sites, further enhancing the stability of the PFNA–HSA complex. Other PFAS–HSA complexes, on the other hand, have larger Rog values and fluctuations, indicating a looser structure.

#### 3.8.4. Number of H-Bonds

**Figure 13:**
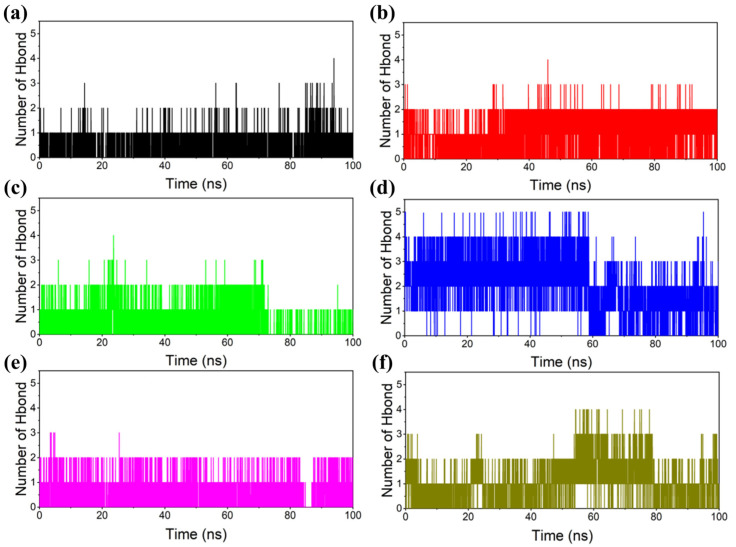
MD simulation of six PFAS binding with HSA. (**a**) HSA–DFSA (**b**) HSA–HFPO-TA (**c**) HSA–PFHpA (**d**) HSA–PFNA (**e**) HSA–PFO3DA (**f**) HSA–PFOA.

The variation in the Number of H-bonds in HSA–PFAS complexes during MD simulation is depicted in Figure 13. As a strong non-covalent binding force, the H-bond is key to complex stability. The H-bond number in the MD simulation varies between 0 and 4, indicating dynamic interactions between PFAS and HSA. Specifically, the HSA–PFNA and HSA–HFPO-TA complexes have 2 stable H-bonds, compared to the 0–2 found in other complex systems, implying more stable interactions that help maintain the structure and function of the complexes.

#### 3.8.5. Binding Free Energy Calculation Results

As shown in Table 4, the binding free energy was calculated using the MM-GBSA method, which provides a more accurate assessment of the binding between PFAS and HSA [71]. Notably, all complexes have negative binding free energies, indicating that all six PFAS can form stable ligand–receptor complexes with HSA. The lowest binding energy (−38.83 kcal/mol) is found in the PFNA–HSA complex, indicating its high affinity for HSA, followed by the HSA–HFPO-TA complex (−35.20 kcal/mol). HSA–DFSA, on the other hand, has a lower affinity (−17.98 kcal/mol). Furthermore, energy decomposition analysis also indicates that van der Waals and electrostatic interactions are the primary driving forces for HSA–PFAS binding.

### 3.9. The Relationship between PFAS Structural Characteristics and Binding Behavior

**Figure 14:**
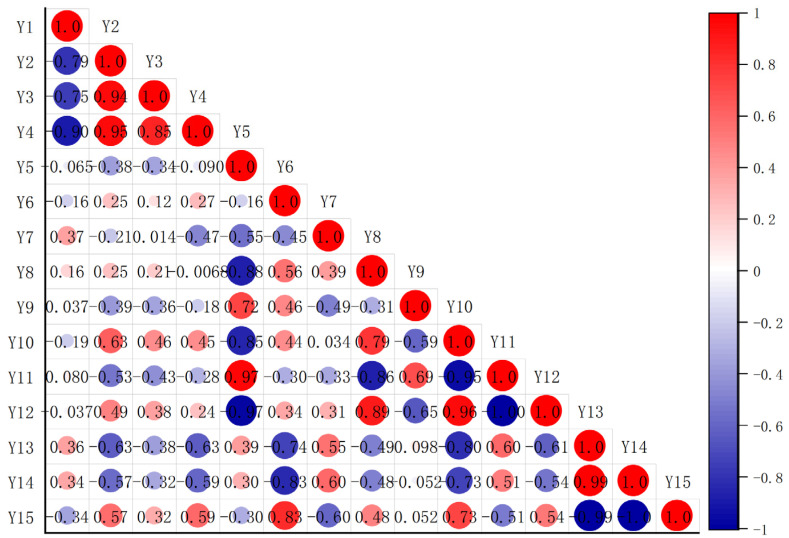
The correlation analysis for the results from multispectral analysis, quantitative calculations, and molecular docking. Y1—*K*_b_, Y2—ΔG, Y3—molecular docking binding energy, Y4—binding free energy,Y5—energy gap (ΔE_HOMO-LUMO)_, Y6—highest ESP maximum, Y7—lowest ESP minimum, Y8—lowest ALIE minimum, Y9—*f*^−^_(r)_ maximum, Y10—Δ*f*_(r)_ minimum, Y11—highest ALIE maximum, Y12—nucleophilicity index, Y13—electrophilicity index, Y14—Mulliken electronegativity, Y15—chemical potential.

Multiple factors influence protein–small molecule interactions, including small molecule structural characteristics, environmental variables, and affinity. The correlation analysis in Figure 14 reveals a significant interrelationship between binding constants, docking binding energies, and molecule structural properties. The binding constant (Y1), in particular, has a significant inverse relationship with Gibbs free energy (Y2, R = 0.79), docking binding energy (Y3, R = 0.75), and binding free energy (Y4, R = 0.90). This inverse relationship emphasizes the importance of binding energy in characterizing energy changes during the molecular binding process. A higher binding energy indicates a more powerful interaction between molecules, which promotes the formation of a stable binding state. Because of this improved interaction, molecules are more likely to aggregate and form stable binding complexes.

Several quantum chemical descriptors, including the lowest ESP minimum (Y7, R = 0.37), highest ALIE maximum (Y11, R = 0.37), electrophilicity index (Y13, R = 0.36), and Mulliken electronegativity (Y14, R = 0.34), show a weak but noticeable positive correlation with the binding constant in this study. These descriptors mainly concern the electrostatic potential and distribution properties of small molecules. The reaction process is generally divided into two stages: the molecular approach (first step) and electronic structural rearrangement (second step). Long-range electrostatic interactions are frequently essential during the molecular approach phase. Only when the molecules are close to each other can the molecular electronic structure be rearranged. Binding reactions between small molecules and proteins are typically driven by weak forces; hence, descriptors related to electrostatic distribution and characteristics like ESP and ALIE are more representative when characterizing the binding. In contrast, descriptors usually used to describe electronic reaction characteristics, such as HOMO and LUMO, show no significant correlation with binding characteristics.

Furthermore, there are numerous quantum chemical descriptors that influence molecular structural features, but this study only considers a subset of them. The samples used in the study are limited to only six PFAS, resulting in a small sample size. To obtain more meaningful analytical results, a broader range of quantum chemical descriptors must be included, as well as an increased experimental data sample size.

### 3.10. Perspective and Application

The increase in binding affinity tends to increase the biological half-lives. As shown in Table 2, the binding constant of PFHpA (4.53 × 10^3^ L·mol^−1^) is significantly lower than that of PFOA (2.27 × 10^5^ L·mol^−1^). Similarly, the half-life of PFHpA (62–70 days) [72] is much shorter than that of PFOA (2.47–4.52 years). This correlation suggests that PFASs’ binding constants with HSA may influence their biochemical behaviors within the human body, affecting their bioaccumulative potential and internal half-lives, as previously observed [73].

Therefore, the binding behavior of PFAS with plasma proteins is key to understanding their bioavailability, toxicological properties, and bioaccumulative potential. Several studies have found significant variations in binding affinity among PFAS of various structures. Long-chain PFAS, such as certain perfluoroalkanoyl chlorides, for example, have a higher binding affinity, whereas binding decreases as the carbon chain length exceeds 11 [74].

The current study is a preliminary investigation into the interrelationship between binding constants and molecular structural properties. Quantum Structure–Activity Relationship (QSAR) models can also be used to predict and interpret their interactions in the future.

Researchers can predict the binding characteristics of new PFAS compounds by developing QSAR models that correlate the PFAS molecular structure with plasma protein binding affinity. For example, PFAS with specific functional groups may form more stable hydrogen or ionic bonds with protein amino acid residues. These models typically rely on experimental data from known compounds combined with statistical or machine learning methods.

Understanding the patterns of interaction between various PFAS and proteins allows for researchers to better predict their behavior in organisms, including their distribution, metabolism, and excretion pathways. This is critical not only for assessing the risk of individual PFAS, but also for understanding complex PFAS mixtures, and providing scientific evidence for risk assessments and environmental regulations.

## 4. Conclusions

This study investigates the interactions between six PFAS and HSA using multi-spectral techniques, Density Functional Theory (DFT), and molecular dynamics approaches.

Fluorescence quenching experiments revealed that four PFAS (PFNA, HFPO-TA, PFOA, and PFO3DA) have a high affinity for HSA, while the other two (PFHpA and DFSA) have a low affinity. PFNA, PFO3DA, PFHpA, and DFSA can easily quench fluorescence groups by generating a complex, resulting in a static quenching process, while the fluorescence quenching of PFOA and HFPO-TA on HSA is a mixed quenching process. The HSA–PFNA complex has the highest binding constant (7.81 × 10^6^ L·mol^−1^) at 298 K, with the binding constants in the following order: PFNA (7.81 × 10^6^ L·mol^−1^) > HFPO-TA (3.70 × 10^6^ L·mol^−1^) > PFOA (2.27 × 10^5^ L·mol^−1^) > PFO3DA (1.59 × 10^5^ L·mol^−1^) > PFHpA (4.53 × 10^3^ L·mol^−1^) > DFSA (1.52 × 10^3^ L·mol^−1^).

Furthermore, synchronous fluorescence, 3D-EEM, and UV-vis spectroscopy show that HSA–PFAS binding changes the microenvironment around the amino acid residues, and causes structural changes in HSA. Molecular docking results show that the binding energy of HSA–PFNA is the lowest (−8.2 kcal·mol^−1^), indicating that PFNA is more likely to bind with HSA. The competitive probe results reveal that six HSA–PFAS binding sites are mainly located in HSA subdomain IIA, which further validates the findings of molecular docking. Molecular dynamics simulation (MD) analysis further shows the lowest binding free energy (−38.83 kcal/mol) in the HSA–PFNA complex, indicating that PFNA binds more readily with HSA.

This study also looked into the quantum chemical descriptors of the six PFAS, such as HOMO, LUMO, ESP, ALIE, and CDFT. Correlation analysis reveals that DFT quantum chemical descriptors related to electrostatic distribution and characteristics, like ESP and ALIE, are more representative when characterizing HSA–PFAS binding. However, the descriptors usually used to describe electronic reaction characteristics, such as HOMO and LUMO, show no significant correlation with binding characteristics. The binding constant (Y1) has a particularly significant inverse relationship with Gibbs free energy (Y2, R = 0.79), docking binding energy (Y3, R = 0.75), and binding free energy (Y4, R = 0.90). A higher binding energy indicates a more powerful interaction between molecules when forming a stable binding state. These findings shed light on the experimental and theoretical mechanisms of HSA–PFAS binding. Researchers can predict the binding characteristics of new PFAS compounds by developing QSAR models that correlate the PFAS molecular structure with the protein binding affinity in the future. Understanding the interactions between various PFAS and proteins allows for researchers to better predict their behavior in organisms, including their distribution, metabolism, and excretion pathways. This is critical not only for assessing the risk of individual PFAS, but also for understanding complex PFAS mixtures, providing scientific evidence for risk assessments and environmental regulations.

## Figures and Tables

**Table 1:** Physico-chemical characteristics of six PFAS.

Compound	Abbreviation	Molecular Formula	Molecular Structure	Relative Molecular Mass	CAS
Perfluorononanoic Acid	PFNA	C_9_HF_17_O_2_	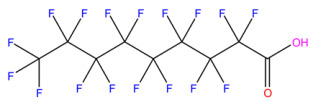	464.08	375-95-1
Perfluoro-2,5-dimethyl-3,6-dioxanonanoic Acid	HFPO-TA	C_9_HF_17_O_4_	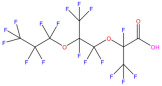	496.07	13252-14-7
Perfluorooctanoic Acid	PFOA	C_8_HF_15_O_2_	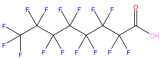	414.07	335-67-1
Perfluoro-3,6,9-trioxadecanoic Acid	PFO3DA	C_7_HF_13_O_5_	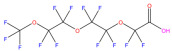	412.06	151772-59-7
Perfluoroheptanoic Acid	PFHpA	C_7_HF_13_O_2_	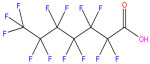	364.06	375-85-9
Dodecafluorosuberic Acid	DFSA	C_8_H_2_F_12_O_4_	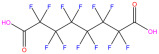	390.08	678-45-5

**Table 2:** Binding constant and thermodynamic parameters of HSA–PFAS binding.

Binding System	*T* (K)	*K*_SV_ × 10^4^ (L·mol^−1^)	*K*_q_ × 10^12^ (L·mol^−1^·s^−1^)	*K*_b_ (L·mol^−1^)	*n*	Δ*H* (kJ·mol^−1^)	Δ*S* (J·mol^−1^·K^−1^)	Δ*G* (kJ·mol^−1^)
HSA–PFNA	298	2.52 ± 0.10	2.52 ± 0.10	(7.81 ± 0.37) × 10^6^	1.51 ± 0.06	−278.25 ± 5.46	−805.8 ± 13.8	−39.32
	304	2.35 ± 0.11	2.35 ± 0.11	(2.02 ± 0.10) × 1 0^5^	1.18 ± 0.03			−30.86
	310	1.27 ± 0.04	1.27 ± 0.04	(9.9 ± 0.36) × 10^4^	1.10 ± 0.04			−29.65
HSA–HFPO-TA	298	1.78 ± 0.09	1.78 ± 0.09	(3.7 ± 0.14) × 10^6^	1.49 ± 0.03	412.15 ± 8.44	1508.3 ± 30.8	−37.47
	304	1.95 ± 0.13	1.95 ± 0.13	(8.54 ± 0.40) × 10^7^	1.78 ± 0.07			−46.16
	310	1.98 ± 0.08	1.98 ± 0.08	(2.31 ± 0.09) × 10^9^	2.09 ± 0.08			−55.57
HSA–PFOA	298	1.39 ± 0.07	1.39 ± 0.07	(2.27 ± 011) × 10^5^	1.26 ± 0.04	146.68 ± 6.39	593.3 ± 22.7	−30.55
	304	1.46 ± 0.09	1.46 ± 0.09	(2.23 ± 0.05) × 10^6^	1.31 ± 0.02			−32.85
	310	1.47 ± 0.11	1.47 ± 0.11	(1.98 ± 0.14) × 10^6^	1.47 ± 0.03			−37.67
HSA–PFO3DA	298	1.05 ± 0.06	1.05 ± 0.06	(1.59 ± 0.06) × 10^5^	1.25 ± 0.03	−106.57 ± 3.92	−259.2 ± 9.4	−29.67
	304	1.03 ± 0.06	1.03 ± 0.06	(4.55 ± 0.27) × 10^4^	1.33 ± 0.02			−27.11
	310	0.87 ± 0.04	0.87 ± 0.04	(2.99 ± 0.08) × 10^4^	1.10 ± 0.03			−26.56
HSA–PFHpA	298	0.69 ± 0.05	0.69 ± 0.05	(4.53 ± 0.34) × 10^3^	0.96 ± 0.04	324.98 ± 14.22	1166.7 ± 42.7	−20.86
	304	0.53 ± 0.07	0.53 ± 0.07	(5.35 ± 0.48) × 10^5^	1.42 ± 0.06			−33.34
	310	0.32 ± 0.04	0.32 ± 0.04	(7.47 ± 0.63) × 10^5^	1.50 ± 0.05			−34.86
HSA–DFSA	298	0.58 ± 0.06	0.58 ± 0.06	(1.52 ± 0.11) × 10^3^	0.88 ± 0.02	167.49 ± 4.28	622.5 ± 15.3	−18.15
	304	0.51 ± 0.04	0.51 ± 0.04	(4.93 ± 0.39) × 10^3^	0.98 ± 0.05			−21.49
	310	0.45 ± 0.05	0.45 ± 0.05	(2.07 ± 0.17) × 10^4^	1.13 ± 0.07			−25.62

**Table 3:** Competition experiment data in the absence and presence of three different site probes. (φ is the rate of decrease in *K*_b_).

System	*K*_b_ (L·mol^−1^)	φ	R^2^
HSA–PFNA	7.81 × 10^6^	-	0.9992
HSA–PFNA–warfarin	1.33 × 10^5^	98.3%	0.9942
HSA–PFNA–ibuprofen	6.73 × 10^6^	13.8%	0.9919
HSA–PFNA–lidocaine	4.38 × 10^6^	43.9%	0.9945
HSA–HFPO-TA	2.70 × 10^6^	-	0.9913
HSA–HFPO-TA–warfarin	1.48 × 10^4^	99.6%	0.9905
HSA–HFPO-TA–ibuprofen	2.82 × 10^6^	23.8%	0.9912
HSA–HFPO-TA–lidocain	2.39 × 10^6^	35.3%	0.9965
HSA–PFOA	2.27 × 10^5^	-	0.9993
HSA–PFOA–warfarin	8.85 × 10^4^	96.1%	0.9994
HSA–PFOA–ibuprofen	2.26 × 10^5^	40.8%	0.9994
HSA–PFOA–lidocaine	2.09 × 10^5^	8.1%	0.9991
HSA–PF03DA	1.59 × 10^5^	-	0.9918
HSA–PF03DA–warfarin	7.79 × 10^3^	95.1%	0.9905
HSA–PF03DA–ibuprofen	1.18 × 10^5^	25.8%	0.9924
HSA–PF03DA–lidocaine	1.40 × 10^5^	11.8%	0.9941
HSA–PFHpA	4.53 × 10^3^	-	0.9933
HSA–PFHpA–warfarin	4.98 × 10^1^	98.9%	0.9924
HSA–PFHpA–ibuprofen	2.60 × 10^3^	42.7%	0.9917
HSA–PFHpA–lidocaine	2.32 × 10^3^	48.8%	0.9961
HSA–DFSA	1.52 × 10^3^	-	0.9927
HSA–DFSA–warfarin	1.02 × 10^2^	93.3%	0.9983
HSA–DFSA–ibuprofen	1.13 × 10^3^	25.4%	0.9905
HSA–DFSA–lidocaine	1.04 × 10^3^	31.7%	0.9945

**Table 4:** Binding free energies and energy components predicted by MM/GBSA (kcal/mol).

System Name	Δ*E*_vdw_	Δ*E*_elec_	ΔG_GB_	ΔG_SA_	ΔG_bind_
HSA–HFPO-TA	−36.91	25.66	−18.10	−5.85	−35.20
HSA–PFO3DA	−21.38	−55.09	58.37	−4.36	−22.46
HSA–PFOA	−20.96	−7.20	4.38	−4.27	−28.04
HSA–PFHpA	−35.30	49.13	−29.24	−5.91	−21.31
HSA–PFNA	−28.64	−29.20	24.50	−5.49	−38.83
HSA–DFSA	−35.18	−20.45	43.33	−5.68	−17.98

**Figure A1:**
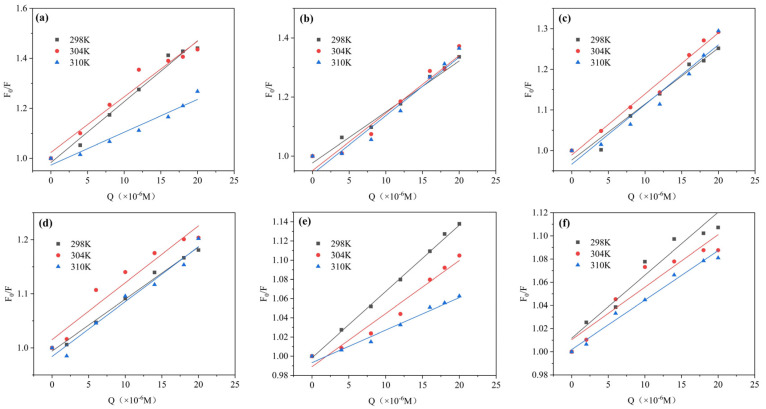
Stern–Volmer plots for quenching of HSA with PFAS at different temperatures. C_[HSA]_ = 1 × 10^−6^ mol·L^−1^, C_[PFAS]_ = 0, 3, 6, 9, 12, 15, and 18 × 10^−6^ mol·L^−1^, T = 298 K, 304 K, 310 K. (**a**) HSA–PFNA (**b**) HSA–HFPO-TA (**c**) HSA–PFOA (**d**) HSA–PFO3DA (**e**) HSA–PFHpA (**f**) HSA–DFSA.

## Data Availability

Data is contained within the article.

## Appendix A

**Table A1:** Characteristic parameters of 3D-EEM of HSA–PFAS binding.

System	Peak A		Peak B	
	Position	Intensity F	Position	Intensity F
	λ_ex_/λ_em_ (nm/nm)	a.u.	λ_ex_/λ_em_ (nm/nm)	a.u.
HSA	280/338	673.66	230/340	996.906
HSA–PFNA	280/329	418.74	230/310	800.96
HSA–HFPO-TA	280/321	499.16	230/314	861.38
HSA–PFOA	280/336	476.59	230/327	812.58
HSA–PFO3DA	280/329	525.07	230/325	886.91
HSA–PFHpA	280/336	656.89	230/335	997.01
HSA–DFSA	280/336	629.75	230/330	839.38

**Table A2:** Conformational changes in the secondary structure of HSA with and without six PFAS.

Sample	Secondary Structure (%)			
	α-Helix	β-Sheet	β-Turn	Random Coil
HSA	41.6	4.9	16.9	36.7
HSA–PFNA	36.2	7.6	15.1	41.2
HSA–HFPO-TA	40.6	5.1	15.8	38.5
HSA–PFOA	37.2	6.4	15.8	40.5
HSA–PFO3DA	36.4	5.8	17.3	40.6
HSA–PFHpA	39.6	5.5	15.5	39.4
HSA–DFSA	43.2	4.0	15.8	37.0

**Table A3:** Calculation of electron density at the free radical front of six PFAS.

Matter	Label	Atom	HOMO Electron Density (×100%)	LUMO Electron Density (×100%)	Radical Frontier Electron Densities (fr Values × 100%)
PFNA	1	F	0.620	0.110	0.730
	2	F	0.620	0.110	0.730
	3	F	0.348	0.046	0.394
	4	F	0.348	0.046	0.394
	5	F	1.077	0.426	1.503
	6	F	1.077	0.426	1.503
	7	F	0.180	0.015	0.195
	8	F	0.180	0.015	0.195
	9	F	2.164	1.172	3.336
	10	F	2.164	1.172	3.336
	11	F	0.087	0.006	0.093
	12	F	0.087	0.006	0.093
	13	F	4.285	5.750	10.035
	14	F	4.285	5.750	10.035
	15	F	0.047	0.001	0.047
	16	F	0.026	0.001	0.027
	17	F	0.026	0.001	0.027
	18	O	8.180	10.095	18.275
	19	O	45.244	25.077	70.321
	20	C	1.447	0.114	1.561
	21	C	0.853	0.044	0.897
	22	C	2.453	0.369	2.822
	23	C	0.471	0.015	0.486
	24	C	3.398	1.886	5.284
	25	C	0.241	0.005	0.246
	26	C	9.747	11.538	21.285
	27	C	0.093	0.002	0.095
	28	C	9.720	34.703	44.423
	29	H	0.533	1.097	1.630
HFPO-TA	1	F	0.039	0.389	0.428
	2	F	0.071	0.166	0.237
	3	F	0.280	1.528	1.808
	4	F	0.001	0.041	0.042
	5	F	0.001	0.035	0.036
	6	F	0.006	0.039	0.045
	7	F	0.125	0.573	0.698
	8	F	0.090	0.192	0.282
	9	F	0.001	0.020	0.020
	10	F	0.001	0.011	0.011
	11	F	4.392	1.647	6.039
	12	F	0.001	0.009	0.009
	13	F	0.001	0.004	0.004
	14	F	0.001	0.004	0.004
	15	F	0.920	2.075	2.995
	16	F	0.965	0.738	1.703
	17	F	0.133	1.095	1.228
	18	O	0.010	0.141	0.151
	19	O	3.743	3.386	7.129
	20	O	8.666	11.385	20.051
	21	O	56.118	24.429	80.547
	22	C	0.044	0.522	0.566
	23	C	0.491	0.901	1.392
	24	C	0.002	0.065	0.067
	25	C	0.070	0.252	0.322
	26	C	0.002	0.036	0.038
	27	C	10.506	9.091	19.597
	28	C	0.001	0.017	0.018
	29	C	1.743	4.328	6.071
	30	C	10.980	35.622	46.602
	31	H	0.601	1.258	1.859
PFOA	1	F	0.569	0.110	0.679
	2	F	0.569	0.110	0.679
	3	F	1.030	0.427	1.457
	4	F	1.030	0.427	1.457
	5	F	0.289	0.046	0.335
	6	F	0.289	0.046	0.335
	7	F	2.148	1.175	3.323
	8	F	2.148	1.175	3.323
	9	F	0.139	0.014	0.153
	10	F	0.139	0.014	0.153
	11	F	4.321	5.757	10.078
	12	F	4.321	5.757	10.078
	13	F	0.074	0.001	0.075
	14	F	0.041	0.004	0.045
	15	F	0.041	0.004	0.045
	16	O	8.269	10.095	18.364
	17	O	46.032	25.067	71.099
	18	C	1.341	0.114	1.455
	19	C	2.369	0.369	2.738
	20	C	0.746	0.044	0.790
	21	C	3.355	1.892	5.247
	22	C	0.381	0.014	0.395
	23	C	9.830	11.552	21.382
	24	C	0.147	0.005	0.152
	25	C	9.840	34.685	44.525
	26	H	0.540	1.096	1.636
PFO3DA	1	F	0.069	0.041	0.110
	2	F	0.069	0.041	0.110
	3	F	0.012	0.001	0.013
	4	F	0.012	0.001	0.013
	5	F	0.172	0.200	0.372
	6	F	0.172	0.200	0.372
	7	F	0.003	0.001	0.004
	8	F	0.003	0.001	0.004
	9	F	3.915	4.575	8.490
	10	F	3.915	4.575	8.490
	11	F	0.001	0.001	0.001
	12	F	0.001	0.001	0.001
	13	F	0.001	0.001	0.001
	14	O	0.066	0.004	0.070
	15	O	1.401	1.626	3.027
	16	O	0.003	0.001	0.003
	17	O	8.353	11.173	19.526
	18	O	60.506	27.230	87.736
	19	C	0.265	0.074	0.339
	20	C	0.026	0.002	0.028
	21	C	0.337	0.453	0.790
	22	C	0.012	0.001	0.013
	23	C	9.200	10.372	19.572
	24	C	0.001	0.001	0.001
	25	C	10.861	38.321	49.182
	26	H	0.624	1.105	1.729
PFHpA	1	F	0.966	0.427	1.393
	2	F	0.966	0.427	1.393
	3	F	0.484	0.108	0.592
	4	F	0.484	0.108	0.592
	5	F	2.103	1.174	3.277
	6	F	2.103	1.174	3.277
	7	F	0.229	0.043	0.272
	8	F	0.229	0.043	0.272
	9	F	4.362	5.756	10.118
	10	F	4.362	5.756	10.118
	11	F	0.118	0.001	0.119
	12	F	0.067	0.012	0.079
	13	F	0.067	0.012	0.079
	14	O	8.384	10.096	18.480
	15	O	47.038	25.084	72.122
	16	C	2.240	0.370	2.610
	17	C	1.195	0.113	1.308
	18	C	3.280	1.892	5.172
	19	C	0.617	0.042	0.659
	20	C	9.923	11.550	21.473
	21	C	0.238	0.013	0.251
	22	C	9.995	34.703	44.698
	23	H	0.550	1.095	1.645
DFSA	1	F	1.125	0.403	1.528
	2	F	1.125	0.403	1.528
	3	F	1.125	0.403	1.528
	4	F	1.125	0.403	1.528
	5	F	1.496	0.706	2.202
	6	F	1.496	0.706	2.202
	7	F	1.496	0.706	2.202
	8	F	1.496	0.706	2.202
	9	F	2.256	2.941	5.197
	10	F	2.256	2.941	5.197
	11	F	2.256	2.941	5.197
	12	F	2.256	2.941	5.197
	13	O	4.144	4.927	9.071
	14	O	4.144	4.927	9.071
	15	O	20.731	12.270	33.001
	16	O	20.731	12.270	33.001
	17	C	2.522	0.374	2.896
	18	C	2.522	0.374	2.896
	19	C	2.552	1.080	3.632
	20	C	2.552	1.080	3.632
	21	C	5.207	5.780	10.987
	22	C	5.207	5.780	10.987
	23	C	4.828	16.929	21.757
	24	C	4.828	16.929	21.757
	25	H	0.261	0.540	0.801
	26	H	0.261	0.540	0.801

**Table A4:** Details of molecular docking results.

PFAS	Binding Site	Amino Acid Residue	Binding Affinity (kcal/mol)
PFNA	Sub-domain IIA	GLN-196, SER-192, LYS-195, ARC-157	−8.2
HFPO-TA	Sub-domain IIA	GLN-29, ASP-249, LYS-106, PHE-149	−7.9
PFOA	Sub-domain IIA	ARG-257, LYS-199, LEU-238	−7.8
PFO3DA	Sub-domain IIA	ASP-249, LYS-106, PHE-149	−7.8
PFHpA	Sub-domain IIA	ARG-257	−7.1
DFSA	Sub-domain IIA	ARG-257	−7.3

Table A5 shows the minimum points for the ESP of PFNA. The points with a smaller ESP value are prone to the electrophilic reaction or electron loss reaction.

**Table A5:** Detailed information of minima points on the ESP map of PFNA.

Number	a.u.	eV	kcal/mol	X/Y/Z Coordinate (Angstrom)		
1	−0.00256476	−0.069791	−1.609415	−2.733042	−4.399004	−1.354155
2	−0.0025635	−0.069756	−1.608622	−2.71706	−4.390909	1.432051
3	−0.00310688	−0.084543	−1.949599	−2.110511	−2.418047	−1.899939
4	−0.00310165	−0.0844	−1.946316	−2.056609	−2.418278	1.952226
5	−0.00182446	−0.049646	−1.144867	−1.943003	0.229967	−2.015871
6	−0.00181719	−0.049448	−1.140304	−2.012692	0.260418	1.957219
7	0.00172291	0.046883	1.081146	−1.689175	2.734329	−2.185543
8	0.00172172	0.046851	1.080399	−1.673282	2.71025	2.206598
9	−0.00072568	−0.019747	−0.455369	−1.030583	−1.098369	−2.708088
10	−0.00072528	−0.019736	−0.455118	−1.033686	−1.101005	2.708316
11	−0.00018964	−0.00516	−0.119002	−1.004442	1.183892	−2.668494
12	−0.0035917	−0.097735	−2.25383	−0.718917	−7.256671	−1.034456
13	−0.00359286	−0.097767	−2.254555	−0.720813	−7.258846	1.031159
14	−0.00312285	−0.084977	−1.959619	1.252509	−6.230336	2.216935
15	−0.00311471	−0.084756	−1.95451	1.262496	−6.208841	−2.235629
16	−0.00329991	−0.089795	−2.070729	1.927462	−3.853978	−1.890087
17	−0.00331016	−0.090074	−2.077158	1.881056	−3.874691	1.946601
18	−0.005165	−0.140547	−3.241087	2.027359	−1.079597	−1.857081
19	−0.00517123	−0.140716	−3.244997	2.089185	−0.957638	1.844006
20	−0.00284419	−0.077394	−1.78476	2.359588	−5.984822	1.488193
21	−0.05153992	−1.402473	−32.341817	3.204304	6.311526	−0.041848

Table A6 shows the maximum points for the ESP of PFNA. The points with a higher ESP value are prone to the nucleophilic reaction.

**Table A6:** Detailed information of maxima points on the ESP map of PFNA.

Number	a.u.	eV	kcal/mol	X/Y/Z Coordinate (Angstrom)		
1	0.00078408	0.021336	0.492018	−2.198569	−3.715034	2.395152
2	0.00174453	0.047471	1.094711	−2.251314	−0.98438	−2.328944
3	0.00377408	0.102698	2.368272	−2.226006	1.815523	−2.299084
4	0.00375375	0.102145	2.355519	−2.314512	1.830511	2.217806
5	0.1369104	3.725522	85.912647	−2.261447	6.450843	−0.057965
6	0.02009527	0.54682	12.609981	−2.100253	−4.932545	−0.049461
7	0.00078732	0.021424	0.494052	−2.185753	−3.728205	−2.40383
8	0.03619023	0.984786	22.709731	−2.165058	−2.386444	−0.005823
9	0.00175421	0.047734	1.100782	−2.172007	−0.952454	2.386437
10	0.03670319	0.998745	23.031619	−2.136952	0.294628	0.00346
11	0.04159386	1.131827	26.100565	−2.099417	2.869966	−0.048825
12	−0.00037348	−0.010163	−0.234359	−1.641064	−7.163151	−0.009394
13	0.02388422	0.649923	14.987588	−0.611037	−5.323351	1.745663
14	0.02387469	0.649663	14.981606	−0.60944	−5.322585	−1.746802
15	0.01446999	0.393748	9.080064	−0.346449	−2.394971	2.126069
16	0.01394373	0.379428	8.74983	−0.331086	0.246292	−2.122432
17	0.01387798	0.377639	8.708568	−0.317189	0.256435	2.126274
18	0.01542621	0.419768	9.680099	−0.328018	2.894377	−2.118471
19	0.01542283	0.419676	9.677978	−0.323027	2.898299	2.11953
20	0.01422157	0.386989	8.924178	−0.317852	−2.396234	−2.136735
21	0.01482924	0.403524	9.305495	0.199664	−3.720799	2.113965
22	0.01378201	0.375028	8.648351	0.20886	−1.063509	2.134037
23	0.05297924	1.441638	33.245	0.166545	5.38545	−1.772435
24	0.05293733	1.440498	33.218701	0.151661	5.380913	1.774217
25	0.01475658	0.401547	9.259899	0.198373	−3.731492	−2.115702
26	0.01361038	0.370357	8.540647	0.183669	−1.086309	−2.142551
27	0.01188333	0.323362	7.456907	0.226042	1.555954	−2.143905
28	0.01183968	0.322174	7.429516	0.235588	1.583181	2.142373
29	0.03807345	1.036031	23.891471	1.241083	−6.547584	−0.044599
30	−0.00232106	−0.063159	−1.456485	1.902554	−0.007932	−2.489981
31	−0.00011733	−0.003193	−0.073624	1.941951	−5.170208	−2.408493
32	−0.00011468	−0.003121	−0.071961	1.951114	−5.166276	2.402661
33	0.03890513	1.058662	24.413359	1.981638	−3.748805	−0.059062
34	−0.00006139	−0.001671	−0.038525	2.038846	−2.535183	−2.391643
35	−0.00005025	−0.001367	−0.03153	1.983586	−2.438736	2.435133
36	−0.00232134	−0.063167	−1.456666	1.922253	0.112879	2.492599
37	0.03352563	0.912279	21.03767	2.049706	−1.078568	−0.01805
38	0.02698529	0.734307	16.933537	2.085531	1.474341	−0.001908

Table A7 shows the minimum points for the ESP of HFPO-TA. The points with a smaller ESP value are prone to the electrophilic reaction or electron loss reaction.

**Table A7:** Detailed information of minima points on the ESP map of HFPO-TA.

Number	a.u.	eV	kcal/mol	X/Y/Z Coordinate (Angstrom)		
1	−0.00461712	−0.125638	−2.897286	−8.472543	−0.593419	0.787227
2	−0.00462201	−0.125771	−2.900359	−8.011784	−2.086933	−0.893139
3	−0.00390569	−0.106279	−2.45086	−7.419505	1.725907	0.868959
4	−0.00346531	−0.094296	−2.174514	−6.965548	−0.920238	−2.959857
5	−0.0038568	−0.104949	−2.420182	−5.189791	2.287263	0.134567
6	−0.00284789	−0.077495	−1.787078	−4.343449	−0.282693	−2.840069
7	−0.00861965	−0.234553	−5.408917	−4.106831	−3.279844	0.723678
8	−0.00862762	−0.234769	−5.413916	−4.167206	−1.489338	2.647718
9	−0.00410926	−0.111819	−2.578604	−2.935282	2.44127	−0.876268
10	−0.00592527	−0.161235	−3.718166	−2.529161	2.37292	1.130759
11	−0.00403446	−0.109783	−2.531661	−2.32427	0.557812	−2.58368
12	−0.00368895	−0.100381	−2.314854	−2.25471	2.528925	−1.079009
13	−0.0074728	−0.203345	−4.689259	−1.789279	−1.255814	−2.382837
14	−0.00329602	−0.089689	−2.068284	−1.306799	0.673228	−2.537822
15	−0.01346982	−0.366532	−8.452447	−0.458952	−4.00293	1.990259
16	−0.00053431	−0.014539	−0.335285	−0.36189	4.993083	−0.823153
17	−0.00398428	−0.108418	−2.500175	−0.04823	1.802948	−2.593018
18	−0.00965328	−0.262679	−6.057532	0.442236	−2.15081	−1.230912
19	−0.01784534	−0.485596	−11.198131	1.001009	−0.841836	3.210719
20	0.00218419	0.059435	1.370604	2.148046	5.437683	−0.695317
21	0.00096094	0.026149	0.603	2.184104	2.552594	−2.980563
22	−0.00387087	−0.105332	−2.42901	2.785979	−2.275365	−3.503317
23	−0.05119253	−1.393019	−32.123822	3.17561	−2.267743	3.401694
24	0.00348316	0.094782	2.185715	3.708534	2.48456	−2.4331
25	0.00427689	0.11638	2.683792	3.711152	3.682457	−1.121281
26	−0.00293275	−0.079804	−1.840332	4.739925	−4.43612	−0.202382
27	0.0085342	0.232228	5.355299	5.388592	0.295737	−2.451385

Table A8 shows the maximum points for the ESP of HFPO-TA. The points with a higher ESP value are prone to the nucleophilic reaction.

**Table A8:** Detailed information of maxima points on the ESP map of HFPO-TA.

Number	a.u.	eV	kcal/mol	X/Y/Z Coordinate (Angstrom)		
1	−0.00171219	−0.046591	−1.074415	−8.396321	−1.809999	0.326795
2	0.03810275	1.036829	23.909856	−7.607055	0.495077	−1.285127
3	0.02269288	0.617505	14.240009	−6.697931	0.098194	1.38515
4	−0.00068683	−0.01869	−0.430993	−6.508742	2.580779	0.320673
5	0.01824909	0.496583	11.451486	−6.227526	−2.019959	0.991107
6	0.02322703	0.63204	14.575197	−6.189733	−2.003867	−1.361603
7	−0.00009279	−0.002525	−0.058226	−5.848933	−0.175032	−3.411765
8	−0.00280735	−0.076392	−1.76164	−5.267418	−0.375107	3.028558
9	0.01400506	0.381097	8.788318	−5.042748	1.151653	1.244851
10	−0.00272062	−0.074032	−1.707215	−4.768302	−3.540596	−0.798403
11	0.03667451	0.997964	23.01362	−4.775801	1.174763	−1.407986
12	0.01454919	0.395904	9.129764	−4.67502	−1.525325	−1.934116
13	0.00938778	0.255455	5.890927	−3.842242	−2.072009	1.38515
14	−0.00093436	−0.025425	−0.586318	−3.67684	2.787862	0.220557
15	0.01848978	0.503133	11.602522	−3.551421	0.549293	1.703955
16	−0.00055419	−0.01508	−0.347763	−3.189026	−0.433902	−3.12563
17	0.02098005	0.570896	13.165192	−2.899639	−2.001149	−0.850287
18	−0.00778084	−0.211728	−4.882557	−2.134889	−3.872607	1.641206
19	0.01227945	0.334141	7.705479	−2.188821	−1.256771	2.443504
20	0.01628279	0.443077	10.217613	−2.068557	−2.493833	−0.148523
21	0.00803789	0.218722	5.043855	−1.981162	0.245841	2.191975
22	0.00088588	0.024106	0.555897	−1.747176	1.950314	−2.372383
23	0.00393749	0.107145	2.470813	−1.593239	2.801703	−0.070088
24	−0.00044019	−0.011978	−0.276227	−1.451576	−0.026298	−2.173926
25	−0.0012484	−0.033971	−0.783385	−1.491151	2.557142	2.256567
26	0.00145774	0.039667	0.914748	−1.196356	4.256947	−0.731559
27	−0.00400782	−0.109058	−2.514949	−0.667179	−1.947917	−2.380787
28	−0.00644659	−0.175421	−4.045298	−0.538395	0.326935	3.613938
29	0.01698084	0.462072	10.655645	−0.413152	−2.864575	−0.089793
30	0.00273705	0.074479	1.717528	−0.140324	2.881141	−1.791041
31	0.0212631	0.578598	13.342806	0.266122	−2.40343	2.704226
32	0.02232602	0.607522	14.009798	0.244535	0.203737	−1.720492
33	0.01093347	0.297515	6.86086	0.457411	0.923757	1.980562
34	0.0418412	1.138557	26.25577	0.889847	4.682522	1.11228
35	0.01709159	0.465086	10.725142	1.289829	0.083739	−1.679882
36	0.02898318	0.788672	18.187234	1.293071	3.802434	−1.574311
37	0.00183059	0.049813	1.148716	1.42062	2.201627	−3.291523
38	0.0008131	0.022125	0.510226	1.420245	2.996346	3.188492
39	0.01404789	0.382263	8.815191	1.58441	1.03329	1.778987
40	0.01626472	0.442586	10.206275	2.352581	−1.364616	−2.067066
41	0.01807087	0.491733	11.339649	2.375591	3.405552	−1.738431
42	0.0180674	0.491639	11.337475	2.650086	3.280063	−1.659148
43	0.02384751	0.648924	14.964554	3.00572	1.011513	−2.235777
44	0.02835888	0.771684	17.795479	3.145662	3.131112	1.335447
45	0.02850928	0.775777	17.889858	3.607252	3.049681	0.874115
46	0.00714982	0.194557	4.486586	3.596452	4.747999	−0.314997
47	0.03733294	1.015881	23.426796	3.799693	−3.670415	−2.118871
48	0.02545209	0.692587	15.971443	4.128417	0.098194	−2.254152
49	0.00233111	0.063433	1.462793	4.340349	−1.36692	−3.935832
50	0.03548586	0.965619	22.267733	5.824356	−1.752347	−1.915001
51	0.13163069	3.581853	82.599572	7.092408	0.077673	0.579258

Table A9 shows the minimum points for the ESP of PFOA. The points with a smaller ESP value are prone to the electrophilic reaction or electron loss reaction.

**Table A9:** Detailed information of minima points on the ESP map of PFOA.

Number	a.u.	eV	kcal/mol	X/Y/Z Coordinate (Angstrom)		
1	−0.00200987	−0.054691	−1.261211	−1.775007	−3.243	2.017137
2	−0.00150071	−0.040836	−0.941708	−1.798098	−0.448829	−2.05791
3	−0.00199814	−0.054372	−1.253854	−1.757805	−3.197558	−2.007395
4	−0.00147032	−0.040009	−0.92264	−1.761719	−0.429531	2.083094
5	−0.00275389	−0.074937	−1.728094	−0.982346	−5.463644	2.349377
6	−0.00275111	−0.074862	−1.726352	−0.942351	−5.446859	−2.361242
7	−0.0005793	−0.015764	−0.363517	−0.870278	−1.767582	−2.705951
8	−0.00057836	−0.015738	−0.362929	−0.875627	−1.770956	2.706464
9	−0.00007298	−0.001986	−0.045797	−0.880745	0.621726	2.682915
10	−0.00006483	−0.001764	−0.040684	−0.835353	0.617191	−2.675381
11	−0.00404254	−0.110003	−2.536735	0.925566	−6.522167	1.085761
12	−0.00404479	−0.110064	−2.538148	1.003173	−6.444512	−1.140274
13	−0.00634261	−0.172591	−3.980051	2.193822	−1.6543	−1.80503
14	−0.00631394	−0.171811	−3.962058	2.242767	−1.710627	1.741491
15	−0.05158322	−1.403651	−32.368987	3.025666	5.819282	−0.00821

Table A10 shows the maximum points for the ESP of PFOA. The points with a higher ESP value are prone to the nucleophilic reaction.

**Table A10:** Detailed information of maxima points on the ESP map of PFOA.

Number	a.u.	eV	kcal/mol	X/Y/Z Coordinate (Angstrom)		
1	0.13704021.	3.729054	85.994099	−2.409296	5.66906	−0.01039
2	0.00399517	0.108714	2.507011	−2.266245	1.03407	−2.253643
3	0.00397656	0.108208	2.495333	−2.302952	1.017791	2.221555
4	0.00223006	0.060683	1.399384	−2.105626	−1.792344	−2.342313
5	0.00223016	0.060686	1.399448	−2.090838	−1.784832	2.354018
6	0.03745099	1.019093	23.50087	−2.056136	−0.440313	−0.038059
7	0.04206036	1.144521	26.393296	−2.116933	2.119702	0.007295
8	0.00088143	0.023985	0.553106	−1.986249	−4.511536	−2.35523
9	0.00088411	0.024058	0.554787	−1.987409	−4.512713	2.354018
10	0.04020066	1.093916	25.226319	−1.962546	−3.095515	−0.063861
11	0.03834027	1.043292	24.058906	−1.175123	−5.922283	−0.058935
12	0.01396082	0.379893	8.760554	−0.298612	−0.41894	−2.115454
13	0.01395474	0.379728	8.756742	−0.300182	−0.420362	2.115262
14	0.01505192	0.409584	9.445231	−0.278421	2.24216	2.139411
15	0.01464266	0.398447	9.188417	−0.169049	−3.066181	−2.117336
16	0.01458243	0.396808	9.15062	−0.162607	−3.063081	2.119685
17	0.01515187	0.412303	9.507951	−0.286165	2.227555	−2.135413
18	0.05308007	1.444382	33.308274	0.059673	4.715223	−1.774456
19	0.05303716	1.443214	33.281346	0.047179	4.711752	1.77596
20	0.01362351	0.370715	8.548892	0.365374	−1.73894	2.123357
21	0.01187587	0.323159	7.452226	0.264205	0.808818	−2.143989
22	0.01194745	0.325107	7.497144	0.261206	0.912525	2.141228
23	0.01353749	0.368374	8.49491	0.349799	−1.757086	−2.128792
24	0.0230319	0.62673	14.452746	0.620425	−4.677041	−1.759885
25	0.02333215	0.6349	14.64116	0.655067	−4.670046	1.741323
26	−0.00113078	−0.03077	−0.709576	1.656236	−6.534042	−0.028127
27	−0.00273802	−0.074505	−1.718133	1.948824	−0.550385	2.515762
28	−0.00120364	−0.032753	−0.755296	2.061528	−3.162191	−2.488254
29	−0.00121152	−0.032967	−0.760241	2.059468	−3.198558	2.485496
30	−0.0027402	−0.074565	−1.719503	1.945421	−0.566356	−2.515328
31	0.01851887	0.503924	11.620777	2.127305	−4.273164	0.038497
32	0.03223493	0.877157	20.227741	2.174863	−1.632658	−0.038597
33	0.02692153	0.732572	16.893526	2.110263	0.921315	−0.001946

Table A11 shows the minimum points for the ESP of PFO3DA. The points with a smaller ESP value are prone to the electrophilic reaction or electron loss reaction.

**Table A11:** Detailed information of minima points on the ESP map of PFO3DA.

Number	a.u.	eV	kcal/mol	X/Y/Z Coordinate (Angstrom)		
1	−0.05255014	−1.429962	−32.975739	−3.28716	6.026046	0.021618
2	−0.00867225	−0.235984	−5.441922	−2.481916	−0.142559	1.842684
3	−0.01618626	−0.440451	−10.157042	−2.479661	2.203244	1.12404
4	−0.0036853	−0.100282	−2.312562	−2.356712	−5.876054	−0.042242
5	−0.00868042	−0.236206	−5.447049	−2.362591	−0.193278	−1.933953
6	−0.016185	−0.440416	−10.15625	−2.404831	2.255065	−1.191848
7	−0.00486167	−0.132293	−3.050747	−2.207291	−4.101209	−0.040243
8	−0.00236537	−0.064365	−1.484296	0.897472	−6.315133	2.365862
9	−0.00236089	−0.064243	−1.481481	0.910678	−6.30216	−2.375327
10	−0.00128105	−0.034859	−0.803869	0.86914	1.592463	−2.68179
11	−0.00129021	−0.035108	−0.809617	0.927075	1.633614	2.690844
12	−0.00093596	−0.025469	−0.587327	1.424469	−0.510601	1.054795
13	−0.0009282	−0.025258	−0.582456	1.415665	−0.518863	−1.066977
14	−0.00434036	−0.118107	−2.723616	1.70143	−3.963915	−2.008507
15	−0.00434557	−0.118249	−2.726887	1.65271	−3.9711	2.040966
16	−0.00103522	−0.02817	−0.649609	1.678634	2.789593	−2.21551
17	−0.00102747	−0.027959	−0.644747	1.836296	2.813763	2.121085
18	−0.00296772	−0.080756	−1.862274	2.197432	0.429843	−1.451889
19	−0.00296614	−0.080713	−1.861282	2.200703	0.438037	1.465588
20	−0.00226113	−0.061529	−1.418883	2.460531	−5.930015	−1.334035
21	−0.00226313	−0.061583	−1.420139	2.466757	−5.928676	1.308587
22	−0.00362853	−0.098737	−2.276941	2.453526	−1.846924	1.301792
23	−0.00362255	−0.098575	−2.273187	2.404248	−1.776566	−1.33238

Table A12 shows the maximum points for the ESP of PFO3DA. The points with a higher ESP value are prone to the nucleophilic reaction.

**Table A12:** Detailed information of maxima points on the ESP map of PFO3DA.

Number	a.u.	eV	kcal/mol	X/Y/Z Coordinate (Angstrom)		
1	−0.00209324	−0.05696	−1.313526	−2.290195	−2.105296	2.43966
2	0.03835598	1.043719	24.068761	−2.395495	−0.365425	0.007326
3	0.01400367	0.381059	8.787441	−2.26331	−3.018514	0.007698
4	−0.00208909	−0.056847	−1.310925	−2.239982	−2.081959	−2.472816
5	−0.00527126	−0.143438	−3.307765	−2.025606	1.003208	2.56989
6	0.0000298	0.000811	0.018699	−2.071707	2.260416	−0.033438
7	−0.0052645	−0.143254	−3.303524	−1.965645	0.968004	−2.595362
8	0.00046446	0.012639	0.291451	−1.795879	−7.271531	−0.032173
9	0.03573752	0.972467	22.425652	−0.800653	−5.130576	−1.562664
10	0.03573142	0.972301	22.421823	−0.777324	−5.118228	1.578095
11	0.02287684	0.622511	14.355449	−0.527908	−3.275863	−1.819096
12	0.02296866	0.625009	14.413067	−0.496951	−3.222527	1.842684
13	0.02069547	0.563152	12.986615	−0.310099	2.273284	−1.864588
14	0.02074901	0.564609	13.020208	−0.296783	2.395436	1.837612
15	0.05415975	1.473762	33.985783	−0.199311	5.291297	−1.772519
16	0.05407664	1.4715	33.933635	−0.1424	5.362713	1.769917
17	0.02236054	0.608461	14.03146	0.091103	−1.147538	−1.803363
18	0.02263293	0.615873	14.202388	0.075628	−1.185359	1.815253
19	0.0220277	0.599404	13.8226	0.116645	0.293412	−1.829727
20	0.0219972	0.598574	13.803461	0.086237	0.290265	1.83966
21	0.03928685	1.06905	24.652894	1.017163	−6.803091	0.022394
22	0.01609798	0.438048	10.101641	1.759392	−1.363067	−0.034657
23	0.01625055	0.4422	10.19738	1.801937	0.309962	0.008531
24	0.00029819	0.008114	0.187115	1.842666	−5.52699	−2.390734
25	0.00031242	0.008501	0.196045	1.892222	−5.549442	2.352285
26	0.04334405	1.179452	27.198825	1.92845	−4.096735	−0.019594
27	−0.00018058	−0.004914	−0.113317	1.943975	−2.500349	−2.392014
28	−0.00018475	−0.005027	−0.115932	1.961573	−2.491781	2.378834
29	0.00134848	0.036694	0.846183	2.074193	1.60188	−2.40639
30	0.00135946	0.036993	0.853073	2.108653	1.619562	2.384041
31	0.04533888	1.233734	28.450602	2.154487	2.909843	−0.041993
32	0.13674252	3.720953	85.807297	2.153761	6.613335	−0.04369

Table A13 shows the minimum points for the ESP of PFHpA. The points with a smaller ESP value are prone to the electrophilic reaction or electron loss reaction.

**Table A13:** Detailed information of minima points on the ESP map of PFHpA.

Number	a.u.	eV	kcal/mol	X/Y/Z Coordinate (Angstrom)		
1	−0.05146101	−1.400325	−32.292301	−3.215565	4.97726	−0.025976
2	−0.00392817	−0.106891	−2.464965	−2.635794	−4.098168	−1.292152
3	−0.00392906	−0.106915	−2.465525	−2.635197	−4.097572	1.295765
4	−0.00498716	−0.135708	−3.129495	−1.902204	−2.436824	−1.880561
5	−0.00498896	−0.135757	−3.130625	−1.899997	−2.511592	1.881956
6	−0.00383752	−0.104424	−2.40808	−1.268736	−4.957645	−2.194388
7	−0.00383647	−0.104396	−2.407422	−1.235018	−4.940963	2.218143
8	−0.00395737	−0.107685	−2.483286	0.719027	−6.008258	0.971913
9	−0.0039506	−0.107501	−2.479044	0.774214	−5.95461	−1.031884
10	−0.0014334	−0.039005	−0.899471	0.994792	−2.310858	−2.699431
11	−0.00143781	−0.039125	−0.90224	1.110826	−2.18313	2.701246
12	0.00171624	0.046701	1.076958	1.669358	1.399664	2.220892
13	−0.0026688	−0.072622	−1.674701	2.068832	−1.113368	−1.943627
14	−0.00267116	−0.072686	−1.676182	2.060152	−1.116604	1.953071
15	−0.00242298	−0.065933	−1.520444	2.616608	−3.254111	−1.491868
16	−0.00242605	−0.066016	−1.522371	2.63958	−3.230077	1.469629

Table A14 shows the maximum points for the ESP of PFHpA. The points with a higher ESP value are prone to the nucleophilic reaction.

**Table A14:** Detailed information of maxima points on the ESP map of PFHpA.

Number	a.u.	eV	kcal/mol	X/Y/Z Coordinate (Angstrom)		
1	0.02756815	0.750168	17.29929	−2.049851	0.205485	−0.038608
2	−0.001146	−0.031184	−0.719129	−1.911846	−3.894457	−2.414155
3	−0.00114733	−0.03122	−0.719959	−1.899997	−3.891549	2.421932
4	0.03723575	1.013236	23.365802	−1.968163	−2.504478	0.004648
5	−0.00226707	−0.06169	−1.422611	−1.927799	−1.258982	−2.46218
6	−0.00226296	−0.061578	−1.420028	−1.899817	−1.244902	2.479951
7	0.03752633	1.021143	23.548145	−1.172053	−5.280314	−0.027178
8	0.01199426	0.32638	7.526517	−0.294143	0.224963	−2.118995
9	0.01202744	0.327283	7.547338	−0.289415	0.228646	2.11961
10	0.0136875	0.372456	8.589041	−0.165429	−2.423117	−2.122351
11	0.01340244	0.364699	8.410164	−0.14398	−2.408379	2.132516
12	0.05314232	1.446076	33.347335	−0.197049	4.038304	−1.775009
13	0.05310459	1.445049	33.323663	−0.212549	4.034761	1.774666
14	0.01376027	0.374436	8.634706	0.370819	−1.094589	2.120416
15	0.01548536	0.421378	9.717216	0.372062	1.551529	−2.113872
16	0.01549804	0.421723	9.725178	0.367062	1.547726	2.114218
17	0.01366925	0.371959	8.577593	0.358264	−1.110429	−2.124776
18	0.02318658	0.630939	14.549808	0.614673	−4.028331	−1.755704
19	0.02341819	0.637241	14.695148	0.655774	−4.023843	1.734827
20	−0.0006096	−0.016588	−0.382533	1.667707	−5.864591	−0.021787
21	0.02008522	0.546547	12.603675	2.115326	−3.62696	−0.004001
22	0.00095343	0.025944	0.598289	2.221779	−2.416539	2.386456
23	0.03635384	0.989238	22.8124	2.168346	−0.990298	−0.046647
24	0.04211295	1.145952	26.4263	2.110935	1.562847	−0.001204
25	0.00095225	0.025912	0.597545	2.217118	−2.432935	−2.388912
26	0.00362541	0.098652	2.274981	2.312435	0.464503	−2.255935
27	0.00362823	0.098729	2.276753	2.264312	0.537349	2.28501
28	0.13711331	3.731043	86.03997	2.246197	5.138156	0.001004

Table A15 shows the minimum points for the ESP of DFSA. The points with a smaller ESP value are prone to the electrophilic reaction or electron loss reaction.

**Table A15:** Detailed information of minima points on the ESP map of DFSA.

Number	a.u.	eV	kcal/mol	X/Y/Z Coordinate (Angstrom)		
1	−0.05174925	−1.408169	−32.473173	−5.546269	−3.034893	−0.020192
2	−0.00193414	−0.052631	−1.213695	−0.642844	2.51297	−2.099547
3	−0.00193249	−0.052586	−1.212659	−0.65777	2.518375	2.091324
4	−0.00192612	−0.052412	−1.208657	0.646666	−2.538931	−2.077621
5	−0.00193105	−0.052546	−1.211751	0.667083	−2.498787	2.106603
6	−0.05174609	−1.408083	−32.47119	5.54625	3.039861	−0.032836

Table A16 shows the maximum points for the ESP of DFSA. The points with a higher ESP value are prone to the nucleophilic reaction.

**Table A16:** Detailed information of maxima points on the ESP map of DFSA.

Number	a.u.	eV	kcal/mol	X/Y/Z Coordinate (Angstrom)		
1	0.05131117	1.396248	32.198269	−2.529019	−3.710461	−1.770272
2	0.0512158	1.393653	32.138424	−2.409386	−3.7152	1.785536
3	0.02659949	0.723809	16.691447	−2.122023	0.548931	−0.016167
4	−0.00095645	−0.026026	−0.600179	−1.328425	1.94552	2.337799
5	−0.00094822	−0.025802	−0.595015	−1.263867	1.983793	−2.348268
6	0.13500128	3.673572	84.714654	−0.936178	−5.950881	−0.028729
7	0.01272843	0.346358	7.987217	−0.769774	−1.82962	2.125084
8	0.01274119	0.346705	7.995224	−0.791081	−1.844413	−2.128081
9	0.03726105	1.013925	23.38168	−0.743958	2.819946	−0.004554
10	0.00940433	0.255905	5.901312	−0.530335	−0.373605	2.139909
11	0.00937479	0.255101	5.882773	−0.526195	−0.393771	−2.143381
12	0.0094777	0.257901	5.947349	0.562386	0.420292	2.137277
13	0.009386	0.255406	5.889808	0.528381	0.396458	−2.143002
14	0.0371607	1.011194	23.318713	0.75833	−2.791169	−0.046819
15	0.01263291	0.343759	7.927275	0.830583	1.879797	2.136512
16	0.01259436	0.34271	7.903088	0.8238	1.856303	−2.136389
17	0.13494306	3.671987	84.67812	0.953251	5.97564	−0.021955
18	−0.00095308	−0.025935	−0.598065	1.299757	−1.995275	2.323033
19	−0.00096143	−0.026162	−0.603305	1.321785	−1.928345	−2.35181
20	0.02658154	0.72332	16.680182	2.127916	−0.537859	−0.019122
21	0.05154275	1.40255	32.343594	2.495309	3.696712	−1.773594

Table A17 shows the minimum points for the ALIE of PFNA. The points with a smaller ALIE value are prone to radical and electrophilic reactions.

**Table A17:** Detailed information of minima points on the ALIE map of PFNA.

Number	a.u.	eV	kcal/mol	X/Y/Z Coordinate (Angstrom)		
1	0.63811477	17.363986	400.423402	−2.55843	−2.677464	−0.635749
2	0.63808464	17.363166	400.404492	−2.577773	−2.739161	0.572579
3	0.6396982	17.407073	401.417018	−2.532831	−2.04712	−0.64876
4	0.63964377	17.405592	401.382862	−2.562024	−1.982565	0.613311
5	0.63970201	17.407177	401.419409	−2.55091	−0.137414	−0.551044
6	0.63821974	17.366842	400.489271	−2.55103	0.701961	−0.554743
7	0.63826734	17.368138	400.51914	−2.581924	0.749257	0.568427
8	0.63199197	17.197376	396.58128	−2.554764	3.345582	0.608968
9	0.63976704	17.408947	401.460218	−2.50661	−0.024462	0.635682
10	0.63202524	17.198281	396.602159	−2.507	3.255412	−0.6251
11	0.63364731	17.24242	397.620026	−2.110344	−5.147755	−0.962784
12	0.63359919	17.241111	397.589826	−2.060921	−5.185703	0.967948
13	0.63916777	17.392639	401.084165	−0.100328	−4.697804	−2.270744
14	0.63917806	17.39292	401.090627	−0.08186	−4.708989	2.283808
15	0.63891857	17.385859	400.927794	−0.13124	−3.735819	−2.329117
16	0.63892489	17.38603	400.93176	−0.127794	−3.652858	2.327643
17	0.63987728	17.411946	401.529392	−0.087898	−1.091609	2.324723
18	0.63989072	17.412312	401.537826	−0.084971	−0.980827	−2.321999
19	0.6402458	17.421974	401.760642	−0.023857	−2.489012	−2.329117
20	0.64024553	17.421967	401.760474	−0.025784	−2.489951	2.327643
21	0.63924233	17.394668	401.130952	0.017447	0.284139	−2.332338
22	0.63340835	17.235918	397.470072	−0.081896	3.881805	−2.334194
23	0.63338188	17.235198	397.453465	−0.07375	3.890861	2.329989
24	0.52951056	14.408715	332.273172	0.044294	7.313668	1.629395
25	0.52951825	14.408924	332.277999	0.023057	7.383718	−1.596969
26	0.63928124	17.395727	401.155372	0.029438	0.394551	2.33419
27	0.63283746	17.220383	397.111832	0.566526	−7.232226	0.006243
28	0.63447313	17.264892	398.138233	1.765984	−6.544735	−0.741585
29	0.63449262	17.265422	398.150465	1.750912	−6.553793	0.751793
30	0.52474424	14.279017	329.282261	1.898895	4.933473	−1.738557
31	0.52471748	14.278289	329.265465	1.875457	4.942688	1.74491
32	0.47201831	12.844271	296.19621	2.053809	7.192063	0.015796
33	0.64281673	17.491933	403.373929	2.341666	−4.056278	−0.643588
34	0.6428381	17.492514	403.387335	2.336142	−4.057804	0.621558
35	0.64080132	17.437091	402.109239	2.421843	−3.312477	−0.545307
36	0.64081347	17.437421	402.116861	2.458191	−3.268825	0.572093
37	0.6401218	17.4186	401.68283	2.448593	−1.516157	−0.536483
38	0.64004808	17.416594	401.63657	2.458191	−1.513274	0.569333
39	0.63909448	17.390645	401.038177	2.458191	−0.723357	0.57751
40	0.63917065	17.392718	401.085974	2.498923	−0.666591	−0.595017
41	0.63498488	17.278817	398.459361	2.525802	1.979295	−0.546146
42	0.63502037	17.279783	398.481633	2.519509	1.979295	0.52844
43	0.52953213	14.409302	332.286705	2.767683	3.907992	0.011061

Table A18 shows the maximum points for the ALIE of PFNA. The points with a higher ALIE value are prone to the nucleophilic reaction.

**Table A18:** Detailed information of maxima points on the ALIE map of PFNA.

Number	a.u.	eV	kcal/mol	X/Y/Z Coordinate (Angstrom)		
1	0.64931641	17.668798	407.452541	−2.6765	−3.790408	−0.000737
2	0.64914569	17.664153	407.345412	−2.64822	−1.096307	−0.017431
3	0.64775662	17.626354	406.473759	−2.612828	1.562766	0.004908
4	0.64298368	17.496476	403.478689	−2.580699	4.413511	0.019389
5	0.73017977	19.869202	458.195109	−2.183294	−3.719198	−2.405607
6	0.65606121	17.852333	411.68497	−2.112417	−4.906367	−0.014373
7	0.73012172	19.867622	458.158683	−2.141914	−3.654847	2.433479
8	0.66906278	18.206124	419.843585	−2.163046	−2.333538	0.021076
9	0.73158381	19.907408	459.076154	−2.161313	−1.071378	−2.397696
10	0.66757232	18.165567	418.908308	−2.136961	0.274651	−0.029032
11	0.66509259	18.09809	417.352249	−2.103068	2.822754	−0.007142
12	0.72295878	19.672709	453.663866	−2.112764	4.297447	−2.382754
13	0.72301374	19.674204	453.698353	−2.092734	4.290379	2.397625
14	0.64349459	17.510378	403.799288	−2.190657	5.266733	−0.000778
15	0.73137403	19.901699	458.944517	−2.078817	−1.086347	2.451609
16	0.72993457	19.86253	458.041241	−2.078636	1.564446	−2.429991
17	0.72995444	19.86307	458.05371	−2.080714	1.563577	2.428408
18	0.72868242	19.828457	457.255504	−1.621744	−7.180741	0.023202
19	0.72563762	19.745604	455.34486	−1.654193	7.584075	0.00717
20	0.66234078	18.023209	415.625464	−0.611037	−5.413587	1.720721
21	0.66231819	18.022594	415.611287	−0.631927	−5.3317	−1.732716
22	0.65083292	17.710064	408.404164	−0.503545	−2.36004	−2.117788
23	0.65081857	17.709674	408.395163	−0.497966	−2.359336	2.116954
24	0.65611006	17.853663	411.715622	−0.293531	−6.010774	1.746055
25	0.65966301	17.950343	413.945133	0.023975	5.260194	−1.838522
26	0.65948548	17.945512	413.833731	0.129811	5.09681	−1.857489
27	0.65986383	17.955808	414.071154	0.069973	5.200356	1.844463
28	0.65073148	17.707304	408.340509	0.352335	−3.726423	−2.102522
29	0.65785599	17.901172	412.811213	0.341482	4.679287	−1.85167
30	0.65067958	17.705892	408.307942	0.404656	−3.672645	2.115972
31	0.65778089	17.899128	412.764089	0.396816	4.519346	−1.850441
32	0.65801088	17.905386	412.908404	0.387909	4.565774	1.844894
33	0.67187868	18.282749	421.610593	1.228644	−6.552897	0.018911
34	0.73185368	19.914751	459.2455	1.972466	−5.090666	−2.3915
35	0.73181299	19.913644	459.219972	1.983478	−5.137788	2.382108
36	0.67185545	18.282117	421.596016	1.977301	−3.735819	0.024162
37	0.73183192	19.914159	459.231851	2.034849	−2.448918	−2.400566
38	0.7318272	19.914031	459.228885	1.987029	−2.383759	2.433479
39	0.73105145	19.892922	458.742094	2.029975	0.178716	2.431281
40	0.66882953	18.199777	419.697221	2.049305	−1.078826	0.008414
41	0.73111002	19.894515	458.77885	2.046257	0.182642	−2.420491
42	0.6664559	18.135187	418.207742	2.082993	1.498262	0.024057
43	0.72704863	19.783999	456.230283	2.059803	2.838483	−2.434952
44	0.72703296	19.783573	456.220451	2.052489	2.836585	2.439711
45	0.64977231	17.681204	407.738624	2.526325	−2.475734	−0.02377
46	0.6486946	17.651878	407.062351	2.565942	0.2916	0.000361
47	0.59709501	16.247781	374.683088	3.423349	5.269825	0.007179

Table A19 shows the minimum points for the ALIE of HFPO-TA. The points with a smaller ALIE value are prone to radical and electrophilic reactions.

**Table A19:** Detailed information of minima points on the ALIE map of HFPO-TA.

Number	a.u.	eV	kcal/mol	X/Y/Z Coordinate (Angstrom)		
1	0.6333077	17.233179	397.406913	−8.333313	−0.060559	−0.960434
2	0.63466419	17.270091	398.258124	−7.701657	1.315301	−0.992569
3	0.6346918	17.270842	398.275452	−7.600871	1.421137	−1.103453
4	0.63527194	17.286629	398.639494	−7.440647	0.519488	−2.213255
5	0.63316307	17.229243	397.316157	−6.447362	−1.501489	1.725164
6	0.63384709	17.247856	397.745384	−6.251494	−2.690639	0.343999
7	0.63824827	17.367619	400.507173	−5.535609	0.947175	1.652036
8	0.63980342	17.409936	401.483046	−5.536107	−1.860095	−2.005135
9	0.64442064	17.535577	404.380397	−5.2186	1.845017	−1.156596
10	0.6381751	17.365628	400.461256	−5.002271	1.000949	1.707067
11	0.64417485	17.528889	404.226161	−5.076194	1.139705	−2.087889
12	0.63812101	17.364156	400.427314	−4.873095	−2.049037	−1.858623
13	0.64257173	17.485266	403.220187	−4.3135	1.738643	−1.414488
14	0.64660402	17.59499	405.750492	−4.214853	0.997795	−2.162525
15	0.63775469	17.354188	400.197446	−3.948078	1.103631	1.656233
16	0.5716844	15.556324	358.737675	−2.756053	−2.457001	0.03069
17	0.57245093	15.577182	359.21868	−2.764373	0.058836	2.151515
18	0.62720704	17.067171	393.578689	−1.883788	−2.653029	−0.731559
19	0.6527038	17.760974	409.578164	−1.891303	2.737063	−0.579823
20	0.64915598	17.664432	407.351867	−1.897566	2.796998	0.366433
21	0.63394443	17.250505	397.806469	−1.433296	−3.137988	−0.03304
22	0.63502281	17.279849	398.483165	−0.735781	−0.129162	−2.213255
23	0.63184846	17.193471	396.491226	−0.244205	−3.235323	2.760887
24	0.63784205	17.356565	400.252263	0.073669	4.968668	0.805099
25	0.6374577	17.346106	400.011084	0.294019	3.219392	−1.998434
26	0.63667636	17.324845	399.52078	0.391176	−2.438952	−0.64347
27	0.63386549	17.248357	397.756936	0.384533	−1.852851	3.390872
28	0.63776082	17.354354	400.201291	0.814518	4.53183	1.955792
29	0.57181289	15.55982	358.818308	1.050783	−0.436873	−1.319544
30	0.58051864	15.796716	364.281252	1.262137	0.578007	1.814092
31	0.63810377	17.363687	400.416499	1.669839	5.247383	0.864014
32	0.64341402	17.508186	403.748734	2.083176	3.984283	−1.686326
33	0.53213377	14.480096	333.919262	2.237788	−0.378066	2.020162
34	0.63812007	17.36413	400.426722	2.456964	3.169419	−2.052505
35	0.64905827	17.661774	407.290558	2.770924	3.377543	2.071531
36	0.63389466	17.249151	397.775237	2.909109	−3.986744	−1.913834
37	0.55939092	15.221801	351.023398	2.924089	0.14781	−2.436845
38	0.64829867	17.641104	406.813901	3.409863	3.537846	1.122938
39	0.63177937	17.191591	396.447873	3.86791	−3.473011	−2.959336
40	0.65227968	17.749433	409.31202	3.803126	2.910267	1.19797
41	0.47101112	12.816864	295.564189	4.323911	−1.735453	3.426719
42	0.63475947	17.272684	398.317914	4.603439	−4.19931	−1.913718
43	0.63333201	17.233841	397.422172	5.035427	−0.172304	−2.551443
44	0.63775572	17.354216	400.198091	5.252497	1.842364	0.004282
45	0.53083409	14.44473	333.103702	5.396771	0.586829	2.788918
46	0.63707292	17.335636	399.769631	6.418906	−1.523692	−0.827429
47	0.52837158	14.377722	331.55845	6.529632	−2.314714	1.896897

Table A20 shows the maximum points for the ALIE of HFPO-TA. The points with a higher ALIE value are prone to the nucleophilic reaction.

**Table A20:** Detailed information of maxima points on the ALIE map of HFPO-TA.

Number	a.u.	eV	kcal/mol	X/Y/Z Coordinate (Angstrom)		
1	0.72975386	19.857612	457.927845	−8.367108	−1.842346	0.339354
2	0.67262773	18.303131	422.080629	−7.631417	0.477485	−1.252149
3	0.66270485	18.033116	415.853921	−6.805635	0.127112	1.335006
4	0.73194701	19.917291	459.30407	−6.478527	2.5845	0.320548
5	0.66349887	18.054722	416.352175	−6.288071	−1.969792	−1.377563
6	0.6578525	17.901077	412.809021	−6.13171	−2.032521	1.067643
7	0.73196148	19.917685	459.31315	−5.763158	−0.27797	−3.401445
8	0.64821268	17.638764	406.759941	−5.238111	−2.435688	1.490985
9	0.72959443	19.853274	457.827801	−5.236772	−0.490229	3.050362
10	0.65045725	17.699842	408.168432	−4.990459	1.230674	1.181184
11	0.66774167	18.170175	419.014576	−4.789553	1.111908	−1.477294
12	0.72917234	19.841788	457.562933	−4.66375	−3.610048	−0.576807
13	0.64779458	17.627387	406.497576	−4.686761	−2.389105	1.597722
14	0.65193	17.739917	409.092595	−4.586467	−1.438158	−2.004347
15	0.6476009	17.622117	406.376038	−4.159748	−2.243485	1.490985
16	0.72806717	19.811715	456.869429	−3.757625	2.764529	0.319871
17	0.64574316	17.571565	405.21029	−3.686761	0.624099	1.702656
18	0.73183048	19.91412	459.230946	−3.01989	−0.49174	−3.096074
19	0.72879253	19.831453	457.324598	−2.059031	−3.903288	1.798666
20	0.65455432	17.811329	410.739384	−2.04304	−1.56279	2.717836
21	0.6545918	17.812349	410.7629	−2.123485	−1.423786	2.564884
22	0.63935328	17.397688	401.200578	−1.848529	−2.643087	−0.139023
23	0.73390661	19.970615	460.533739	−1.747124	1.950314	−2.37232
24	0.7303634	19.874199	458.310334	−1.600196	2.393373	2.341695
25	0.73485359	19.996383	461.127978	−1.254568	4.172859	−0.683936
26	0.72348531	19.687036	453.994267	−0.564174	−1.997717	−2.33129
27	0.72985913	19.860477	457.993902	−0.46485	0.132424	3.769161
28	0.66206673	18.015752	415.453495	−0.329417	−2.878498	−0.056928
29	0.6694424	18.216454	420.081803	0.28534	−2.448062	2.655175
30	0.6750326	18.368571	423.589709	0.924239	4.706368	1.070747
31	0.73488123	19.997135	461.145319	1.123343	2.687266	3.170694
32	0.6517687	17.735528	408.991377	1.231419	−0.485872	2.121306
33	0.72544786	19.74044	455.225787	1.225918	2.156336	−3.291585
34	0.6657158	18.115048	417.743323	1.268414	3.88437	−1.538247
35	0.72805369	19.811348	456.860972	1.505267	−3.468053	−0.425463
36	0.65384515	17.792031	410.294372	2.253827	−1.648091	−2.124549
37	0.64017466	17.420038	401.716003	2.931227	1.403258	−2.362091
38	0.66161351	18.003419	415.169096	3.007259	3.210544	1.482423
39	0.73583373	20.023054	461.743027	3.455855	4.912726	−0.404503
40	0.73495108	19.999036	461.189154	3.688994	1.324272	2.658312
41	0.65330247	17.777264	409.953834	3.859439	−3.976471	−0.031695
42	0.67018738	18.236726	420.54928	3.83047	−3.712241	−2.054861
43	0.65896484	17.931345	413.507027	3.862962	2.960255	0.701723
44	0.72841812	19.821265	457.089657	3.994297	−1.529609	−4.015827
45	0.63972236	17.407731	401.432178	4.35751	−0.060559	−2.374504
46	0.72776367	19.803457	456.678981	4.785849	2.991948	−1.684078
47	0.65676408	17.87146	412.126031	5.012207	1.201793	0.411743
48	0.55772347	15.176427	349.977056	5.155523	−1.326582	3.295731
49	0.66068248	17.978084	414.584861	5.765548	−1.760622	−2.018923
50	0.72998749	19.86397	458.074449	6.247707	−3.870636	−0.484307
51	0.64085816	17.438637	402.144905	6.247095	−1.727455	−0.394266
52	0.72092281	19.617307	452.386276	6.689096	0.416411	−1.366572
53	0.72506515	19.730026	454.985635	7.219034	−0.071063	1.56644

Table A21 shows the minimum points for the ALIE of PFOA. The points with a smaller ALIE value are prone to radical and electrophilic reactions.

**Table A21:** Detailed information of minima points on the ALIE map of PFOA.

Number	a.u.	eV	kcal/mol	X/Y/Z Coordinate (Angstrom)		
1	0.63840523	17.37189	400.605667	−2.498296	−0.015889	0.522351
2	0.63191734	17.195345	396.534448	−2.565444	2.546935	−0.621485
3	0.63196923	17.196757	396.567009	−2.57339	2.546368	0.649549
4	0.64060539	17.431759	401.986285	−2.444421	−2.614197	−0.529998
5	0.6406248	17.432287	401.998467	−2.452198	−2.621974	0.565555
6	0.63989404	17.412402	401.53991	−2.449946	−0.85997	−0.596384
7	0.63989524	17.412435	401.540664	−2.443072	−0.85997	0.574681
8	0.63841808	17.372239	400.613728	−2.473511	−0.110845	−0.625857
9	0.63442079	17.263468	398.105388	−1.725215	−5.90634	−0.725319
10	0.63441388	17.26328	398.101056	−1.728271	−5.91105	0.754973
11	0.63276202	17.21833	397.064497	−0.51987	−6.582752	−0.02779
12	0.52953856	14.409477	332.290744	−0.185783	6.654347	−1.635733
13	0.63339042	17.23543	397.458822	−0.136438	3.283199	2.336571
14	0.63339171	17.235465	397.459629	−0.134031	3.283676	−2.334735
15	0.52952468	14.409099	332.282033	−0.132763	6.669561	1.622856
16	0.64005585	17.416806	401.641449	0.04265	−1.814712	2.323701
17	0.64006734	17.417118	401.648656	0.046689	−1.808874	−2.320693
18	0.63915853	17.392388	401.078369	0.15649	−4.161611	−2.263432
19	0.63912533	17.391485	401.057538	0.112342	−4.056271	2.283411
20	0.63879646	17.382536	400.851165	0.152836	−2.977661	−2.331017
21	0.6393189	17.396752	401.179002	0.076808	−0.314574	−2.319353
22	0.63932308	17.396866	401.181628	0.079041	−0.314913	2.320817
23	0.63881424	17.383019	400.862322	0.16715	−3.079718	2.34017
24	0.52475504	14.279311	329.289033	1.797567	4.379513	−1.746478
25	0.52475588	14.279334	329.289562	1.797952	4.379898	1.746477
26	0.47184942	12.839676	296.090232	1.900766	6.59942	0.024485
27	0.63351971	17.238948	397.539956	2.061528	−4.537855	−1.046724
28	0.63354396	17.239608	397.555169	2.163986	−4.465516	0.95161
29	0.63792671	17.358869	400.305391	2.584485	−2.02585	−0.640319
30	0.63795253	17.359571	400.321591	2.585746	−2.025469	0.646271
31	0.63901426	17.388462	400.987837	2.542515	−1.331502	−0.578188
32	0.63902398	17.388727	400.993939	2.590705	−1.243499	0.56817
33	0.63513877	17.283005	398.55593	2.556135	1.457816	−0.54711
34	0.63514101	17.283066	398.557337	2.555206	1.46841	0.528356
35	0.52970865	14.414105	332.397475	2.721702	3.364539	0.011467

Table A22 shows the maximum points for the ALIE of PFOA. The points with a higher ALIE value are prone to the nucleophilic reaction.

**Table A22:** Detailed information of maxima points on the ALIE map of PFOA.

Number	a.u.	eV	kcal/mol	X/Y/Z Coordinate (Angstrom)		
1	0.64298177	17.496424	403.477489	−2.66271	3.614336	0.022803
2	0.64960833	17.676741	407.63572	−2.509567	−1.922931	−0.013639
3	0.64796246	17.631955	406.602923	−2.584721	0.841246	0.005706
4	0.64350538	17.510672	403.806062	−2.281822	4.5579	0.01084
5	0.7228963	19.671009	453.624656	−2.190399	3.575726	−2.379031
6	0.72299463	19.673684	453.686362	−2.17189	3.565329	2.392815
7	0.66558043	18.111364	417.658373	−2.115909	2.159404	−0.026584
8	0.73164572	19.909093	459.115008	−1.960219	−4.433655	2.376786
9	0.73168005	19.910027	459.13655	−2.029705	−1.794461	−2.398687
10	0.66804193	18.178345	419.202994	−2.059281	−0.53924	−0.001778
11	0.73002283	19.864932	458.096629	−2.05105	0.84235	−2.429732
12	0.73002932	19.865108	458.1007	−2.050598	0.84187	2.429911
13	0.73171104	19.91087	459.155996	−1.92604	−4.441356	−2.400857
14	0.67155779	18.274017	421.409232	−1.957816	−3.079489	0.001391
15	0.73143506	19.90336	458.982816	−1.944459	−1.808313	2.453932
16	0.72568945	19.747014	455.377388	−1.846182	6.863406	0.003347
17	0.67174428	18.279092	421.526256	−1.190503	−5.911203	0.013512
18	0.65053138	17.701859	408.214947	−0.383846	−3.087853	2.113169
19	0.65059214	17.703512	408.253072	−0.371364	−3.079094	−2.110074
20	0.65987646	17.956152	414.07908	−0.055181	4.537638	−1.856845
21	0.65980206	17.954127	414.03239	−0.027704	4.555208	1.835734
22	0.65792107	17.902943	412.852051	0.262325	4.00846	−1.851845
23	0.65810042	17.907823	412.964596	0.303259	3.943559	1.839316
24	0.65048546	17.70061	408.186133	0.572187	−1.710075	−2.130974
25	0.65044142	17.699411	408.158497	0.488838	−1.700872	2.115124
26	0.66222742	18.020124	415.554326	0.623304	−4.752495	1.736019
27	0.66228936	18.02181	415.593197	0.675944	−4.685845	−1.726329
28	0.72844702	19.822051	457.107789	1.739489	−6.455609	0.006019
29	0.72689611	19.779849	456.134579	2.049003	2.205796	2.427619
30	0.72694801	19.781261	456.167146	2.055465	2.214034	−2.423235
31	0.655932	17.848817	411.603887	2.167363	−4.203289	0.020154
32	0.72993114	19.862436	458.03909	2.164737	−2.977376	2.432196
33	0.73093965	19.889879	458.671937	2.091694	−0.419341	−2.44113
34	0.73106134	19.893191	458.748302	2.151306	−0.339279	2.401074
35	0.66673682	18.142832	418.384022	2.108379	0.939232	0.023362
36	0.7299505	19.862963	458.05124	2.175157	−2.974636	−2.425208
37	0.66902814	18.205181	419.821846	2.17008	−1.704662	0.000878
38	0.64919848	17.665589	407.37854	2.693988	−3.168615	0.005919
39	0.64867543	17.651356	407.05032	2.645917	−0.381416	−0.02532
40	0.59696002	16.244108	374.598385	3.308064	4.63723	−0.015481

Table A23 shows the minimum points for the ALIE of PFO3DA. The points with a smaller ALIE value are prone to radical and electrophilic reactions.

**Table A23:** Detailed information of minima points on the ALIE map of PFO3DA.

Number	a.u.	eV	kcal/mol	X/Y/Z Coordinate (Angstrom)		
1	0.63691676	17.331386	399.671634	−2.898749	−0.91548	−0.565905
2	0.63694208	17.332075	399.687525	−2.907297	−0.949033	0.53654
3	0.63640414	17.317437	399.349962	−2.893576	0.202298	−0.609969
4	0.6363292	17.315398	399.302936	−2.884345	0.213927	0.561437
5	0.50289722	13.684529	315.573032	−2.387982	3.547339	0.112889
6	0.47093756	12.814863	295.518031	−2.229194	7.01214	0.043477
7	0.50109997	13.635624	314.445241	−2.073625	3.791768	−1.050226
8	0.5011294	13.636424	314.463709	−2.105888	3.741207	0.969541
9	0.63974606	17.408375	401.447047	−1.417052	−5.678731	−1.464674
10	0.63978301	17.409381	401.470238	−1.481837	−5.68911	1.433934
11	0.5653333	15.383501	354.752297	−1.18715	−4.018122	−1.64388
12	0.56532478	15.383269	354.74695	−1.205382	−4.02098	1.635067
13	0.561448	15.277777	352.314236	−1.106218	3.158002	−1.573369
14	0.56143176	15.277335	352.304046	−1.049156	3.160017	1.604554
15	0.63524285	17.285837	398.621242	−0.358698	−1.58109	−2.271804
16	0.63783056	17.356252	400.245052	−0.252863	−5.115933	2.194718
17	0.6352119	17.284995	398.601821	−0.333162	−1.587138	2.251711
18	0.63487838	17.275919	398.392531	−0.252863	0.774628	−2.262532
19	0.52892098	14.392672	331.903204	−0.263476	7.384357	1.59136
20	0.63781219	17.355752	400.233529	−0.242389	−5.08793	−2.200817
21	0.6350606	17.280878	398.506876	−0.140732	−2.872876	2.249933
22	0.63490174	17.276555	398.407188	−0.238957	0.735189	2.244975
23	0.52885101	14.390768	331.859298	−0.202229	7.338233	−1.625712
24	0.63509192	17.28173	398.526529	−0.112547	−2.856156	−2.276878
25	0.63414012	17.25583	397.929265	0.007865	1.889992	−2.262301
26	0.63416594	17.256533	397.945472	0.011699	1.995827	2.239567
27	0.63123719	17.176837	396.10765	0.121894	4.089249	−2.254108
28	0.63122061	17.176386	396.097248	0.170479	4.059014	2.29188
29	0.63989533	17.412437	401.540717	0.32039	−7.379452	−0.044738
30	0.5701646	15.514968	357.783991	0.651586	−0.462609	−1.676345
31	0.57022753	15.51668	357.823478	0.675885	−0.450027	1.666115
32	0.63831025	17.369305	400.546067	1.54634	−6.840396	−0.723826
33	0.638329	17.369815	400.557831	1.54634	−6.842228	0.733434
34	0.641745	17.46277	402.701406	2.347911	−4.468637	−0.644264
35	0.64174452	17.462757	402.701106	2.352162	−4.47721	0.642649
36	0.63692801	17.331693	399.678698	2.456664	−3.401781	−0.530183
37	0.63691236	17.331267	399.668875	2.46207	−3.401781	0.546199
38	0.63528606	17.287013	398.648358	2.636757	2.336225	0.562661
39	0.63529762	17.287327	398.655608	2.61221	2.423963	−0.615152
40	0.62921902	17.12192	394.841229	2.678548	3.477524	−0.632012
41	0.62923063	17.122236	394.848511	2.678892	3.477524	0.633643

Table A24 shows the maximum points for the ALIE of PFO3DA. The points with a higher ALIE value are prone to the nucleophilic reaction.

**Table A24:** Detailed information of maxima points on the ALIE map of PFO3DA.

Number	a.u.	eV	kcal/mol	X/Y/Z Coordinate (Angstrom)		
1	0.59606914	16.219866	374.039348	−3.406011	4.97501	0.030687
2	0.65168328	17.733204	408.937776	−2.404956	−2.879598	−0.006436
3	0.72685866	19.77883	456.111076	−2.440884	−1.903436	−2.38267
4	0.72684388	19.778428	456.101806	−2.459067	−1.917844	2.367614
5	0.67104139	18.259965	421.08518	−2.398103	−0.33903	−0.000883
6	0.7263825	19.765873	455.812282	−2.36791	1.15698	2.360939
7	0.65037646	17.697643	408.117731	−2.367745	1.994922	0.025523
8	0.72638734	19.766005	455.815317	−2.334722	1.167668	−2.382344
9	0.73686518	20.051121	462.390269	−1.855831	−7.215043	0.006821
10	0.55631377	15.138067	349.092452	−1.449249	7.499271	0.211367
11	0.66391746	18.066113	416.614845	−0.793099	−5.204562	−1.575364
12	0.66401261	18.068702	416.674553	−0.79234	−5.216215	1.578035
13	0.64244389	17.481787	403.139964	−0.515351	−2.978439	−1.938834
14	0.64225882	17.476751	403.023831	−0.502296	−3.016202	1.92206
15	0.64140354	17.453478	402.487135	−0.374211	2.101663	−1.922064
16	0.64147062	17.455303	402.529228	−0.358698	2.037726	1.963959
17	0.6582239	17.911183	413.042079	−0.379424	4.312985	−1.719687
18	0.65829363	17.913081	413.085835	−0.366456	4.335255	1.732003
19	0.6573612	17.887708	412.500726	−0.252863	−6.471009	1.647817
20	0.65742373	17.88941	412.539966	−0.200967	−6.524949	−1.62445
21	0.66020908	17.965203	414.287801	−0.062983	5.170891	−1.837287
22	0.64261897	17.486551	403.24983	0.064644	−1.437482	−1.934591
23	0.64260727	17.486233	403.242486	0.042471	−1.395358	1.914595
24	0.64203046	17.470537	402.880533	0.064644	0.492472	−1.943435
25	0.64200344	17.469802	402.86358	0.064644	0.453922	1.92206
26	0.65989553	17.95667	414.091042	−0.036142	5.274066	1.813079
27	0.65524256	17.830057	411.171258	0.39762	−7.00858	0.975774
28	0.67419123	18.345676	423.061739	1.003125	−6.807623	0.012569
29	0.72661784	19.772277	455.959961	1.466889	7.721221	0.032951
30	0.73098579	19.891135	458.70089	1.863846	−5.501247	−2.380239
31	0.73100362	19.89162	458.712084	1.879136	−5.503299	2.369452
32	0.67224615	18.292748	421.841181	1.921157	−4.04799	−0.013272
33	0.72595192	19.754156	455.542092	1.969682	−2.534455	−2.378138
34	0.72586426	19.751771	455.487081	1.921616	−2.472254	2.403171
35	0.65113927	17.718401	408.596406	1.929641	−1.536783	−0.002951
36	0.64982749	17.682705	407.773248	2.028343	0.514131	−0.006277
37	0.7246019	19.71742	454.69494	2.065575	1.477981	−2.399331
38	0.72463689	19.718373	454.716897	2.05638	1.45655	2.402057
39	0.66645412	18.135139	418.206624	2.149364	2.940585	−0.003015
40	0.71795485	19.536545	450.523846	2.181353	4.55567	−2.381249
41	0.71797024	19.536964	450.533507	2.2482	4.523806	2.338611
42	0.64732857	17.614706	406.20515	2.275749	5.379919	0.009344
43	0.655114	17.826558	411.090584	2.374878	−5.417472	0.005656

Table A25 shows the minimum points for the ALIE of PFHpA. The points with a smaller ALIE value are prone to radical and electrophilic reactions.

**Table A25:** Detailed information of minima points on the ALIE map of PFHpA.

Number	a.u.	eV	kcal/mol	X/Y/Z Coordinate (Angstrom)		
1	0.52970838	14.414098	332.397306	−2.764695	2.572858	0.016782
2	0.63540866	17.290349	398.725286	−2.486323	0.63169	0.525609
3	0.63541346	17.29048	398.728302	−2.536889	0.71717	−0.529897
4	0.64006655	17.417097	401.64816	−2.41535	−2.028974	−0.538874
5	0.64005189	17.416698	401.638961	−2.44483	−1.986814	0.54715
6	0.47191765	12.841532	296.133047	−2.146681	5.840994	0.01329
7	0.5248336	14.281449	329.338334	−1.90068	3.648544	−1.747687
8	0.52488749	14.282915	329.372148	−1.872719	3.691128	1.75988
9	0.63430798	17.260398	398.034602	−1.788782	−5.235571	−0.741568
10	0.63427294	17.259444	398.012613	−1.724986	−5.269619	0.754121
11	0.63261253	17.214262	396.97069	−0.492151	−5.972199	−0.012232
12	0.52959508	14.411015	332.326208	−0.060915	6.030524	−1.616159
13	0.52962982	14.41196	332.34801	−0.027746	6.044434	1.613447
14	0.63959238	17.404194	401.350613	0.05022	−1.159251	−2.312514
15	0.63956083	17.403335	401.330819	0.041035	−1.064918	2.319297
16	0.63903248	17.388958	400.999274	0.116696	−3.388831	−2.270362
17	0.63902568	17.388773	400.995002	0.112212	−3.413709	2.276079
18	0.63863486	17.378138	400.749762	0.149403	−2.334359	−2.336194
19	0.6386565	17.378727	400.763338	0.15734	−2.324394	2.341751
20	0.63353656	17.239406	397.550526	0.076282	2.548124	−2.322396
21	0.6335366	17.239408	397.550554	0.07678	2.546825	2.32255
22	0.63329682	17.232883	397.400086	2.014745	−3.918008	−1.059074
23	0.63327483	17.232284	397.38629	2.060574	−3.893981	0.964358
24	0.63783332	17.356327	400.246789	2.578423	−1.383905	−0.647438
25	0.63782418	17.356079	400.241053	2.567673	−1.383919	0.612871
26	0.6383078	17.369239	400.544528	2.533538	−0.70158	−0.594368
27	0.63833989	17.370112	400.564662	2.598009	−0.584283	0.581284
28	0.63213879	17.201371	396.673411	2.534902	2.008777	−0.593004
29	0.63214852	17.201636	396.679516	2.547343	2.008777	0.634293

Table A26 shows the maximum points for the ALIE of PFHpA. The points with a higher ALIE value are prone to the nucleophilic reaction.

**Table A26:** Detailed information of maxima points on the ALIE map of PFHpA.

Number	a.u.	eV	kcal/mol	X/Y/Z Coordinate (Angstrom)		
1	0.59715342	16.249371	374.719742	−3.428364	3.918411	0.003175
2	0.6492129	17.665981	407.387584	−2.500813	−1.382014	−0.012644
3	0.73099165	19.891294	458.704573	−2.05875	−1.166286	2.382994
4	0.73165524	19.909352	459.12098	−1.975392	−3.823955	−2.374379
5	0.73172501	19.91125	459.164764	−1.952915	−3.834467	2.389865
6	0.6714057	18.269878	421.313789	−1.966177	−2.439367	−0.00911
7	0.73118091	19.896444	458.823331	−2.028661	−1.151378	−2.404846
8	0.66701893	18.150508	418.561046	−2.051435	0.224843	0.013517
9	0.72716429	19.787147	456.302866	−2.048263	1.48583	−2.431227
10	0.72716268	19.787103	456.301855	−2.061268	1.480324	2.422164
11	0.67171522	18.278301	421.50802	−1.194763	−5.267825	0.015083
12	0.65029982	17.695558	408.069641	−0.384088	−2.440533	2.110947
13	0.65821004	17.910806	413.033382	−0.382218	3.269777	−1.843628
14	0.65816586	17.909604	413.00566	−0.365383	3.323582	1.843263
15	0.65034832	17.696878	408.100074	−0.362171	−2.435279	−2.106316
16	0.66005934	17.961128	414.193836	−0.06651	3.902984	−1.84118
17	0.66001415	17.959898	414.165482	−0.047877	3.89384	1.854771
18	0.64998859	17.687089	407.874343	0.493927	−1.054444	−2.116793
19	0.64997276	17.686658	407.864407	0.487746	−1.060008	2.115687
20	0.66216958	18.018551	415.518035	0.622436	−4.110785	1.727948
21	0.6620053	18.01408	415.414945	0.667845	−4.029418	−1.72764
22	0.72851032	19.823774	457.147512	1.64549	−5.884708	0.021551
23	0.72567013	19.746488	455.365264	1.667083	6.251435	0.012727
24	0.72299946	19.673816	453.689393	2.055819	2.957937	2.427444
25	0.72306953	19.675722	453.733358	2.062794	2.967046	−2.422204
26	0.65569305	17.842315	411.453944	2.129523	−3.600502	−0.022781
27	0.72980387	19.858973	457.959229	2.167999	−2.431724	−2.4227
28	0.72978442	19.858444	457.947022	2.161868	−2.333872	2.427567
29	0.66869619	18.196149	419.613548	2.163415	−1.067258	−0.00883
30	0.72990976	19.861855	458.025676	2.174668	0.261503	−2.383007
31	0.72999485	19.86417	458.079067	2.155863	0.305392	2.395445
32	0.66558203	18.111408	417.659382	2.111743	1.618309	0.026886
33	0.6436586	17.514841	403.902211	2.157216	4.014776	−0.012264
34	0.64319389	17.502196	403.610599	2.590003	3.065286	−0.005716
35	0.64895598	17.65899	407.226368	2.681329	−2.548404	−0.014505
36	0.64801125	17.633283	406.633541	2.638546	0.345437	0.025021

Table A27 shows the minimum points for the ALIE of DFSA. The points with a smaller ALIE value are prone to radical and electrophilic reactions.

**Table A27:** Detailed information of minima points on the ALIE map of DFSA.

Number	a.u.	eV	kcal/mol	X/Y/Z Coordinate (Angstrom)		
1	0.47176366	12.837342	296.036412	−5.036839	−4.321914	0.021566
2	0.53009117	14.424514	332.637511	−4.021707	−1.185442	0.001255
3	0.52473329	14.278719	329.275389	−3.720042	−2.473581	−1.733779
4	0.52467758	14.277203	329.240427	−3.705399	−2.514063	1.74569
5	0.52944867	14.407031	332.234334	−3.395921	−5.532939	−1.591188
6	0.52944138	14.406833	332.229759	−3.354809	−5.497441	1.61987
7	0.63467498	17.270384	398.264896	−2.75288	0.363996	−0.543653
8	0.63469973	17.271058	398.280426	−2.75288	0.358646	0.528432
9	0.63699964	17.333642	399.723646	−2.278623	1.056586	−0.584843
10	0.63700462	17.333777	399.726767	−2.277601	1.08321	0.580368
11	0.63320976	17.230514	397.345456	−1.474252	−2.559816	−2.317118
12	0.63321737	17.230721	397.350231	−1.477265	−2.570835	2.32056
13	0.63181548	17.192573	396.470531	−0.923937	3.382336	−0.615125
14	0.63182928	17.192949	396.479191	−0.911791	3.380737	0.570318
15	0.63184326	17.193329	396.487964	0.932393	−3.312837	−0.646613
16	0.63183267	17.193041	396.481317	0.924468	−3.31443	0.615627
17	0.63327755	17.232358	397.387992	1.489392	2.626725	2.340562
18	0.63326542	17.232028	397.380385	1.485633	2.614857	−2.336818
19	0.6370273	17.334394	399.741003	2.295757	−0.987586	−0.65379
20	0.63471394	17.271445	398.289345	2.766061	−0.349628	−0.524442
21	0.63472602	17.271773	398.296927	2.754966	−0.355913	0.524926
22	0.52949863	14.408391	332.265687	3.279741	5.476363	−1.652922
23	0.52943753	14.406728	332.227343	3.324275	5.496099	1.631321
24	0.52473811	14.27885	329.278409	3.677734	2.51297	1.746642
25	0.5247035	14.277908	329.256693	3.725943	2.557571	−1.755348
26	0.52943072	14.406543	332.223073	3.93625	1.163988	0.016325
27	0.47180249	12.838399	296.060779	5.051765	4.306327	−0.018835

Table A28 shows the maximum points for the ALIE of DFSA. The points with a higher ALIE value are prone to the nucleophilic reaction.

**Table A28:** Detailed information of maxima points on the ALIE map of DFSA.

Number	a.u.	eV	kcal/mol	X/Y/Z Coordinate (Angstrom)		
1	0.59711237	16.248254	374.693983	−5.187095	−1.981966	−0.025987
2	0.7264121	19.766678	455.830855	−2.820207	−0.556258	−2.396453
3	0.72640508	19.766487	455.826449	−2.752067	−0.556244	2.434979
4	0.6598242	17.95473	414.046286	−2.274018	−3.649702	−1.850264
5	0.65978989	17.953796	414.024755	−2.275824	−3.625486	1.852171
6	0.65767439	17.89623	412.697259	−2.268402	−3.220391	1.859593
7	0.65784526	17.90088	412.804476	−2.241119	−2.999618	−1.846282
8	0.65781093	17.899946	412.78294	−2.223702	−2.939755	1.849175
9	0.6653816	18.105954	417.533608	−2.117867	0.558208	−0.023806
10	0.7255519	19.743271	455.291076	−2.012349	−6.583775	0.00033
11	0.64699701	17.605684	405.997093	−1.890447	1.889491	0.001947
12	0.72891381	19.834753	457.400704	−1.444764	1.687977	−2.39687
13	0.72895773	19.835949	457.428268	−1.422839	1.620466	2.43347
14	0.6640284	18.069132	416.684463	−0.802896	2.724641	0.020874
15	0.6433462	17.50634	403.706174	−0.422229	−4.892435	0.003014
16	0.64293787	17.495229	403.449946	−0.417325	4.309077	0.002483
17	0.72280347	19.668483	453.566405	−0.055239	3.994666	−2.383206
18	0.72277604	19.667736	453.549195	−0.004075	3.964412	2.423623
19	0.72279749	19.66832	453.56265	0.048779	−3.998891	−2.385024
20	0.72279027	19.668123	453.558122	0.083254	−3.955321	2.383002
21	0.64321202	17.502689	403.621974	0.422184	4.893617	−0.026803
22	0.64285102	17.492866	403.395444	0.454331	−4.260499	0.015737
23	0.66478446	18.089705	417.158899	0.760175	−2.778802	0.009666
24	0.72893059	19.83521	457.411235	1.423671	−1.614612	−2.434961
25	0.72897096	19.836309	457.436566	1.446935	−1.619244	2.419962
26	0.64693379	17.603964	405.957425	1.920346	−1.836894	0.004695
27	0.7255505	19.743233	455.290197	2.009716	6.582976	0.023207
28	0.66563329	18.112803	417.691545	2.117738	−0.555104	−0.000577
29	0.6577282	17.897695	412.731024	2.221386	2.914058	−1.84743
30	0.65789796	17.902314	412.837551	2.252868	2.95276	1.829939
31	0.65761273	17.894552	412.658564	2.274245	3.200842	−1.852924
32	0.65976438	17.953102	414.008745	2.271587	3.621525	−1.855583
33	0.65982781	17.954828	414.04855	2.27337	3.651266	1.850441
34	0.72661688	19.772251	455.95936	2.766534	0.592119	−2.439673
35	0.72662377	19.772438	455.96368	2.775212	0.614054	2.442536
36	0.59708799	16.24759	374.678683	5.193966	1.991242	0.001022

Table A29 shows the detailed CDFT descriptors, indicating the possible reactive sites of the PFNA molecule.

**Table A29:** Detailed information of CDFT descriptors of PFNA.

Atom	q(N)	q(N + 1)	q(N − 1)	*f*_(r)_^−^	*f*_(r)_^+^	*f*_(r)_^0^	Δ*f*_(r)_	Electrophilicity	Nucleophilicity	*s^−^*	*s^+^*	*s*_0_	*s^+^/s^−^*	*s^−^/s^+^*
1(F)	−0.0911	−0.097	−0.0837	0.0074	0.0059	0.0067	−0.0015	0.01445	−0.01245	0.0295	0.0236	0.0265	0.8007	1.2489
2(F)	−0.0911	−0.097	−0.0837	0.0074	0.0059	0.0067	−0.0015	0.01445	−0.01245	0.0295	0.0236	0.0265	0.8007	1.2489
3(F)	−0.0907	−0.0937	−0.087	0.0037	0.003	0.0034	−0.0007	0.00729	−0.00626	0.0148	0.0119	0.0134	0.804	1.2438
4(F)	−0.0907	−0.0937	−0.087	0.0037	0.003	0.0034	−0.0007	0.00729	−0.00626	0.0148	0.0119	0.0134	0.804	1.2438
5(F)	−0.0923	−0.1032	−0.0777	0.0145	0.0109	0.0127	−0.0036	0.02658	−0.02445	0.0579	0.0434	0.0506	0.7501	1.3332
6(F)	−0.0923	−0.1032	−0.0777	0.0145	0.0109	0.0127	−0.0036	0.02658	−0.02445	0.0579	0.0434	0.0506	0.7501	1.3332
7(F)	−0.0907	−0.0923	−0.0888	0.0019	0.0016	0.0017	−0.0003	0.00386	−0.00322	0.0076	0.0063	0.007	0.8286	1.2069
8(F)	−0.0907	−0.0923	−0.0888	0.0019	0.0016	0.0017	−0.0003	0.00386	−0.00322	0.0076	0.0063	0.007	0.8286	1.2069
9(F)	−0.0924	−0.119	−0.0639	0.0285	0.0266	0.0276	−0.0019	0.06494	−0.04792	0.1134	0.1061	0.1097	0.935	1.0695
10(F)	−0.0924	−0.119	−0.0639	0.0285	0.0266	0.0276	−0.0019	0.06494	−0.04792	0.1134	0.1061	0.1097	0.935	1.0695
11(F)	−0.0917	−0.0925	−0.0906	0.001	0.0009	0.001	−0.0002	0.00211	−0.00177	0.0042	0.0034	0.0038	0.8231	1.2148
12(F)	−0.0917	−0.0925	−0.0906	0.001	0.0009	0.001	−0.0002	0.00211	−0.00177	0.0042	0.0034	0.0038	0.8231	1.2148
13(F)	−0.095	−0.2159	−0.0112	0.0838	0.1209	0.1023	0.0371	0.29473	−0.14092	0.3336	0.4813	0.4074	1.443	0.693
14(F)	−0.095	−0.2159	−0.0112	0.0838	0.1209	0.1023	0.0371	0.29473	−0.14092	0.3336	0.4813	0.4074	1.443	0.693
15(F)	−0.0919	−0.0927	−0.091	0.0009	0.0008	0.0009	−0.0001	0.00192	−0.00157	0.0037	0.0031	0.0034	0.848	1.1792
16(F)	−0.0914	−0.0919	−0.0909	0.0006	0.0005	0.0005	−0.0001	0.00121	−0.00096	0.0023	0.002	0.0021	0.8698	1.1497
17(F)	−0.0914	−0.0919	−0.0909	0.0006	0.0005	0.0005	−0.0001	0.00121	−0.00096	0.0023	0.002	0.0021	0.8698	1.1497
18(O)	−0.1486	−0.2676	−0.0096	0.1391	0.119	0.129	−0.0201	0.29012	−0.23394	0.5538	0.4738	0.5138	0.8557	1.1687
19(O)	−0.2623	−0.4745	0.0507	0.313	0.2122	0.2626	−0.1008	0.5174	−0.52659	1.2464	0.845	1.0457	0.6779	1.4751
20(C)	0.1816	0.1791	0.1851	0.0035	0.0025	0.003	−0.001	0.00611	−0.00591	0.014	0.01	0.012	0.7131	1.4022
21(C)	0.1818	0.1805	0.1835	0.0017	0.0013	0.0015	−0.0004	0.00314	−0.0028	0.0066	0.0051	0.0059	0.7727	1.2942
22(C)	0.1808	0.1781	0.1876	0.0068	0.0027	0.0048	−0.0041	0.00664	−0.01148	0.0272	0.0108	0.019	0.3991	2.5056
23(C)	0.1814	0.1807	0.1822	0.0009	0.0007	0.0008	−0.0001	0.00179	−0.00144	0.0034	0.0029	0.0032	0.8598	1.163
24(C)	0.1817	0.1778	0.1917	0.01	0.0039	0.0069	−0.0062	0.00944	−0.01687	0.0399	0.0154	0.0277	0.3863	2.5887
25(C)	0.1772	0.1768	0.1776	0.0005	0.0004	0.0004	−0.0001	0.00097	−0.0008	0.0019	0.0016	0.0017	0.8366	1.1953
26(C)	0.1909	0.1221	0.2521	0.0612	0.0688	0.065	0.0076	0.16772	−0.10296	0.2437	0.2739	0.2588	1.124	0.8897
27(C)	0.279	0.2786	0.2794	0.0004	0.0004	0.0004	−0.0001	0.00088	−0.00074	0.0017	0.0014	0.0016	0.8272	1.2089
28(C)	0.2117	0.0226	0.3244	0.1127	0.1891	0.1509	0.0764	0.46115	−0.18962	0.4488	0.7531	0.601	1.678	0.596
29(H)	0.2095	0.1525	0.2757	0.0662	0.057	0.0616	−0.0092	0.13893	−0.11136	0.2636	0.2269	0.2452	0.8608	1.1617

**Note:** q(N): Hirshfeld charges in neutral state; q(N+1): Hirshfeld charges in a positive charge state; q(N−1): Hirshfeld charges in a negative charge state; *f*_(r)_^−^: electrophilic Fukui function; *f*_(r)_*^+^*: nucleophilic Fukui function; *f*_(r)_^0^: radical Fukui function; Δ*f*_(r)_: condensed dual descriptors; electrophilicity: reduced local electrophilic index; nucleophilicity: reduced local nucleophilic index; *s^−^*: reduced local softness; *s^+^*: relative electrophilic index*; s*_0_: relative nucleophilic index; E(N): −2190.661502 Hartree; E(N+1): −2190.748081 Hartree; E(N−1): −2190.323797 Hartree; E_HOMO(N): −0.397022 Hartree, −10.8035 eV; E_HOMO(N+1): −0.165714 Hartree, −4.5093 eV; E_HOMO(N−1): −0.43199 Hartree, −11.755 eV; vertical IP: 0.225392 Hartree, 6.1332 eV; vertical EA: 0.071224 Hartree, 1.9381 eV; Mulliken electronegativity: 0.212142 Hartree, 5.7727 eV; chemical potential:−0.212142 Hartree, −5.7727 eV; hardness (=fundamental gap):0.251126 Hartree, 6.8335 eV; Softness: 3.982066 Hartree^−1^, 0.1463 eV^−1^; electrophilicity index: 0.089605 Hartree, 2.4383 eV; nucleophilicity index: −0.061824 Hartree, −1.6823 eV.

Table A30 shows the detailed CDFT descriptors, indicating the possible reactive sites of the HFPO-TA molecule.

**Table A30:** Detailed information of CDFT descriptors of HFPO-TA.

Atom	q(N)	q(N + 1)	q(N − 1)	*f*_(r)_ *^−^*	*f*_(r)_ *^+^*	*f*_(r)_^0^	Δ*f*_(r)_	Electrophilicity	Nucleophilicity	*s^−^*	*s^+^*	*s*_0_	*s^+^/s^−^*	*s^−^/s^+^*
1(F)	−0.0961	−0.1258	−0.0865	0.0096	0.0298	0.0197	0.0202	0.10554	−0.01764	0.0454	0.1412	0.0933	3.1109	0.3214
2(F)	−0.0974	−0.2034	−0.0627	0.0346	0.106	0.0703	0.0714	0.37604	−0.06384	0.1643	0.5031	0.3337	3.0624	0.3265
3(F)	−0.0836	−0.1895	−0.067	0.0166	0.1058	0.0612	0.0892	0.37532	−0.03064	0.0789	0.5021	0.2905	6.3679	0.157
4(F)	−0.091	−0.1044	−0.0867	0.0042	0.0134	0.0088	0.0092	0.04755	−0.00779	0.02	0.0636	0.0418	3.1751	0.3149
5(F)	−0.0973	−0.1082	−0.0927	0.0046	0.0109	0.0077	0.0063	0.03867	−0.00845	0.0218	0.0517	0.0367	2.3785	0.4204
6(F)	−0.0913	−0.1123	−0.0786	0.0127	0.021	0.0168	0.0083	0.07443	−0.02338	0.0602	0.0996	0.0799	1.655	0.6042
7(F)	−0.0906	−0.1016	−0.0888	0.0019	0.0109	0.0064	0.009	0.03867	−0.00346	0.0089	0.0517	0.0303	5.8174	0.1719
8(F)	−0.0913	−0.1061	−0.0861	0.0053	0.0148	0.01	0.0095	0.05239	−0.0097	0.025	0.0701	0.0475	2.8072	0.3562
9(F)	−0.0906	−0.0967	−0.088	0.0026	0.0061	0.0043	0.0036	0.02175	−0.00472	0.0121	0.0291	0.0206	2.3963	0.4173
10(F)	−0.0913	−0.0978	−0.0886	0.0026	0.0065	0.0046	0.0039	0.0231	−0.00482	0.0124	0.0309	0.0216	2.493	0.4011
11(F)	−0.0873	−0.0671	−0.0028	0.0844	−0.0201	0.0321	−0.1046	−0.07145	−0.15561	0.4005	−0.0956	0.1524	−0.2387	−4.1892
12(F)	−0.0907	−0.0954	−0.0888	0.002	0.0046	0.0033	0.0027	0.01638	−0.0036	0.0093	0.0219	0.0156	2.3647	0.4229
13(F)	−0.0912	−0.0946	−0.0906	0.0007	0.0034	0.002	0.0027	0.01209	−0.00123	0.0032	0.0162	0.0097	5.1031	0.196
14(F)	−0.0914	−0.0939	−0.0899	0.0015	0.0024	0.002	0.001	0.00868	−0.00269	0.0069	0.0116	0.0093	1.6761	0.5966
15(F)	−0.0899	−0.1063	−0.056	0.0339	0.0164	0.0252	−0.0175	0.05823	−0.06259	0.1611	0.0779	0.1195	0.4837	2.0675
16(F)	−0.0881	−0.1081	−0.0604	0.0277	0.02	0.0239	−0.0077	0.07103	−0.05116	0.1317	0.095	0.1133	0.7218	1.3854
17(F)	−0.0903	−0.1191	−0.0636	0.0267	0.0288	0.0278	0.0021	0.10217	−0.04929	0.1269	0.1367	0.1318	1.0775	0.9281
18(O)	−0.1405	−0.1526	−0.1352	0.0053	0.0121	0.0087	0.0067	0.04286	−0.00984	0.0253	0.0573	0.0413	2.2651	0.4415
19(O)	−0.137	−0.4135	−0.0844	0.0526	0.2765	0.1646	0.2239	0.98059	−0.09701	0.2496	1.3118	0.7807	5.2551	0.1903
20(O)	−0.1471	−0.1935	−0.0139	0.1332	0.0463	0.0898	−0.0869	0.16434	−0.24555	0.6319	0.2199	0.4259	0.3479	2.8742
21(O)	−0.2681	−0.317	−0.0092	0.2589	0.0489	0.1539	−0.21	0.17338	−0.47727	1.2282	0.2319	0.7301	0.1888	5.2952
22(C)	0.1574	0.144	0.1606	0.0032	0.0134	0.0083	0.0103	0.04758	−0.00583	0.015	0.0636	0.0393	4.2432	0.2357
23(C)	0.2541	0.1833	0.27	0.0159	0.0708	0.0433	0.0549	0.25092	−0.02929	0.0754	0.3357	0.2055	4.4537	0.2245
24(C)	0.257	0.2524	0.2584	0.0015	0.0046	0.003	0.0031	0.0163	−0.00268	0.0069	0.0218	0.0144	3.1586	0.3166
25(C)	0.281	0.2751	0.2842	0.0032	0.0059	0.0045	0.0028	0.021	−0.00582	0.015	0.0281	0.0215	1.8755	0.5332
26(C)	0.1759	0.1724	0.1775	0.0016	0.0034	0.0025	0.0018	0.01222	−0.00302	0.0078	0.0163	0.0121	2.101	0.476
27(C)	0.1612	0.1118	0.2238	0.0626	0.0494	0.056	−0.0132	0.17514	−0.11545	0.2971	0.2343	0.2657	0.7886	1.268
28(C)	0.2795	0.2775	0.2801	0.0006	0.002	0.0013	0.0014	0.00725	−0.00117	0.003	0.0097	0.0064	3.2262	0.31
29(C)	0.2842	0.2819	0.3033	0.0191	0.0023	0.0107	−0.0168	0.00804	−0.03523	0.0907	0.0108	0.0507	0.1186	8.4287
30(C)	0.1929	0.1789	0.3019	0.109	0.014	0.0615	−0.0949	0.04977	−0.2009	0.517	0.0666	0.2918	0.1288	7.7651
31(H)	0.2006	0.1315	0.2625	0.0619	0.0691	0.0655	0.0072	0.24509	−0.11407	0.2935	0.3279	0.3107	1.117	0.8952

**Note:** q(N): Hirshfeld charges in neutral state; q(N+1): Hirshfeld charges in a positive charge state; q(N−1): Hirshfeld charges in a negative charge state; *f*_(r)_^−^: electrophilic Fukui function; *f*_(r)_*^+^*: nucleophilic Fukui function; *f*_(r)_^0^: radical Fukui function; Δ*f*: condensed dual descriptors; electrophilicity: reduced local electrophilic index; nucleophilicity: reduced local nucleophilic index; *s^−^*: reduced local softness; *s^+^*: relative electrophilic index*; s*_0_: relative nucleophilic index; E(N): −1383.391208 Hartree; E(N+1): −1383.487614 Hartree; E(N−1): −1383.166034 Hartree; E_HOMO(N): −0.40295 Hartree, −10.9648 eV; E_HOMO(N+1):−0.314238 Hartree, −8.5509 eV; E_HOMO(N−1): −0.453262 Hartree,−12.3339 eV; vertical IP: 0.33978 Hartree, 9.2459 eV; vertical EA:0.129003 Hartree, 3.5103 eV; Mulliken electronegativity: 0.234392 Hartree, 6.3781 eV; chemical potential: −0.234392 Hartree, −6.3781 eV; hardness (=fundamental gap): 0.210778 Hartree, 5.7356 eV; softness: 4.744334 Hartree^−1^, 0.1744 eV^−1^; electrophilicity index: 0.130325 Hartree, 3.5463 eV; nucleophilicity index: −0.067752 Hartree, −1.8436 eV.

Table A31 shows the detailed CDFT descriptors, indicating the possible reactive sites of the PFOA molecule.

**Table A31:** Detailed information of CDFT descriptors of PFOA.

Atom	q(N)	q(N + 1)	q(N − 1)	*f*_(r)_ *^−^*	*f*_(r)_ *^+^*	*f*_(r)_^0^	Δ*f*_(r)_	Electrophilicity	Nucleophilicity	*s^−^*	*s^+^*	*s*_0_	*s^+^/s^−^*	*s^−^/s^+^*
1(F)	−0.0909	−0.0969	−0.0836	0.0074	0.0059	0.0066	−0.0015	0.0144	−0.01243	0.0293	0.0235	0.0264	0.8016	1.2476
2(F)	−0.0909	−0.0969	−0.0836	0.0074	0.0059	0.0066	−0.0015	0.0144	−0.01243	0.0293	0.0235	0.0264	0.8016	1.2476
3(F)	−0.0922	−0.1031	−0.0777	0.0145	0.011	0.0127	−0.0035	0.0267	−0.02439	0.0576	0.0436	0.0506	0.7571	1.3209
4(F)	−0.0922	−0.1031	−0.0777	0.0145	0.011	0.0127	−0.0035	0.0267	−0.02439	0.0576	0.0436	0.0506	0.7571	1.3209
5(F)	−0.0909	−0.0939	−0.0872	0.0037	0.003	0.0034	−0.0007	0.00733	−0.00624	0.0147	0.012	0.0134	0.8131	1.2298
6(F)	−0.0909	−0.0939	−0.0872	0.0037	0.003	0.0034	−0.0007	0.00733	−0.00624	0.0147	0.012	0.0134	0.8131	1.2298
7(F)	−0.0924	−0.119	−0.064	0.0284	0.0266	0.0275	−0.0017	0.06497	−0.04787	0.113	0.1061	0.1096	0.9387	1.0653
8(F)	−0.0924	−0.119	−0.064	0.0284	0.0266	0.0275	−0.0017	0.06497	−0.04787	0.113	0.1061	0.1096	0.9387	1.0653
9(F)	−0.0917	−0.0934	−0.0897	0.002	0.0017	0.0018	−0.0003	0.00407	−0.00339	0.008	0.0066	0.0073	0.8293	1.2058
10(F)	−0.0917	−0.0934	−0.0897	0.002	0.0017	0.0018	−0.0003	0.00407	−0.00339	0.008	0.0066	0.0073	0.8293	1.2058
11(F)	−0.095	−0.216	−0.0117	0.0834	0.121	0.1022	0.0377	0.29504	−0.14055	0.3319	0.4819	0.4069	1.4519	0.6888
12(F)	−0.095	−0.216	−0.0117	0.0834	0.121	0.1022	0.0377	0.29504	−0.14055	0.3319	0.4819	0.4069	1.4519	0.6888
13(F)	−0.092	−0.0934	−0.0903	0.0017	0.0014	0.0016	−0.0003	0.00349	−0.00286	0.0068	0.0057	0.0062	0.845	1.1834
14(F)	−0.0915	−0.0924	−0.0904	0.0011	0.0009	0.001	−0.0002	0.0022	−0.00183	0.0043	0.0036	0.004	0.8336	1.1996
15(F)	−0.0915	−0.0924	−0.0904	0.0011	0.0009	0.001	−0.0002	0.0022	−0.00183	0.0043	0.0036	0.004	0.8336	1.1996
16(O)	−0.1485	−0.2674	−0.0093	0.1392	0.1189	0.129	−0.0203	0.28986	−0.2347	0.5542	0.4734	0.5138	0.8542	1.1707
17(O)	−0.2623	−0.4741	0.0529	0.3152	0.2118	0.2635	−0.1034	0.51631	−0.53152	1.2551	0.8433	1.0492	0.6719	1.4884
18(C)	0.1818	0.1792	0.1853	0.0035	0.0026	0.003	−0.001	0.00624	−0.00596	0.0141	0.0102	0.0121	0.724	1.3812
19(C)	0.1809	0.1781	0.1876	0.0067	0.0027	0.0047	−0.004	0.0067	−0.01138	0.0269	0.0109	0.0189	0.4074	2.4544
20(C)	0.1813	0.18	0.1829	0.0016	0.0013	0.0015	−0.0003	0.00318	−0.00274	0.0065	0.0052	0.0058	0.8031	1.2452
21(C)	0.1817	0.1778	0.1917	0.01	0.0039	0.0069	−0.0061	0.00946	−0.01685	0.0398	0.0155	0.0276	0.3884	2.5748
22(C)	0.1772	0.1764	0.1781	0.0009	0.0008	0.0009	−0.0002	0.0019	−0.00158	0.0037	0.0031	0.0034	0.8333	1.2001
23(C)	0.191	0.122	0.2517	0.0607	0.069	0.0648	0.0083	0.16819	−0.10236	0.2417	0.2747	0.2582	1.1365	0.8799
24(C)	0.2789	0.2783	0.2798	0.0008	0.0007	0.0007	−0.0001	0.00167	−0.00137	0.0032	0.0027	0.003	0.838	1.1933
25(C)	0.2117	0.0227	0.3243	0.1126	0.189	0.1508	0.0764	0.46068	−0.18988	0.4484	0.7524	0.6004	1.6781	0.5959
26(H)	0.2095	0.1525	0.2753	0.0658	0.0569	0.0614	−0.0089	0.1388	−0.11096	0.262	0.2267	0.2444	0.8652	1.1558

**Note:** q(N): Hirshfeld charges in neutral state; q(N+1): Hirshfeld charges in a positive charge state; q(N−1): Hirshfeld charges in a negative charge state; *f*_(r)_^−^: electrophilic Fukui function; *f*_(r)_*^+^*: nucleophilic Fukui function; *f*_(r)_^0^: radical Fukui function; Δ*f*_(r)_: condensed dual descriptors; electrophilicity: reduced local electrophilic index; nucleophilicity: reduced local nucleophilic index; *s^−^*: reduced local softness; *s^+^*: relative electrophilic index*; s*_0_: relative nucleophilic index; E(N): −1952.944666 Hartree; E(N+1): −1953.031224 Hartree; E(N−1): −1952.606956 Hartree; E_HOMO(N): −0.397162 Hartree, −10.8073 eV; E_HOMO(N+1): −0.165717 Hartree, −4.5094 eV; E_HOMO(N−1): −0.437355 Hartree, −11.901 eV; vertical IP: 0.33771 Hartree, 9.1896 eV; vertical EA: 0.086558 Hartree, 2.3554 eV; Mulliken electronegativity: 0.212134 Hartree, 5.7725 eV; chemical potential: −0.212134 Hartree, −5.7725 eV; hardness (=fundamental gap): 0.251152 Hartree, 6.8342 eV; softness: 3.981655 Hartree^−1^, 0.1463 eV^−1^; electrophilicity index: 0.089589 Hartree, 2.4378 eV; nucleophilicity index: −0.061964 Hartree, −1.6861 eV.

Table A32 shows the detailed CDFT descriptors, indicating the possible reactive sites of the PFO3DA molecule.

**Table A32:** Detailed information of CDFT descriptors of PFO3DA.

Atom	q(N)	q(N + 1)	q(N − 1)	*f*_(r)_ *^−^*	*f*_(r)_ *^+^*	*f*_(r)_^0^	Δ*f*_(r)_	Electrophilicity	Nucleophilicity	*s^−^*	*s^+^*	*s*_0_	*s^+^/s^−^*	*s^−^/s^+^*
1(F)	−0.0955	−0.1015	−0.0898	0.0056	0.006	0.0058	0.0004	0.01387	−0.01054	0.0219	0.0233	0.0226	1.0662	0.9379
2(F)	−0.0955	−0.1015	−0.0898	0.0056	0.006	0.0058	0.0004	0.01387	−0.01054	0.0219	0.0233	0.0226	1.0662	0.9379
3(F)	−0.0952	−0.098	−0.0924	0.0028	0.0028	0.0028	0	0.00656	−0.00529	0.011	0.011	0.011	1.0057	0.9943
4(F)	−0.0952	−0.098	−0.0924	0.0028	0.0028	0.0028	0	0.00656	−0.00529	0.011	0.011	0.011	1.0057	0.9943
5(F)	−0.0971	−0.1159	−0.079	0.018	0.0188	0.0184	0.0007	0.04334	−0.03376	0.0701	0.0729	0.0715	1.0398	0.9617
6(F)	−0.0971	−0.1159	−0.079	0.018	0.0188	0.0184	0.0007	0.04334	−0.03376	0.0701	0.0729	0.0715	1.0398	0.9617
7(F)	−0.0965	−0.0973	−0.0957	0.0008	0.0008	0.0008	0	0.00186	−0.00144	0.003	0.0031	0.0031	1.046	0.9561
8(F)	−0.0965	−0.0973	−0.0957	0.0008	0.0008	0.0008	0	0.00186	−0.00144	0.003	0.0031	0.0031	1.046	0.9561
9(F)	−0.1022	−0.2002	−0.049	0.0532	0.098	0.0756	0.0448	0.22628	−0.09947	0.2066	0.3808	0.2937	1.8431	0.5426
10(F)	−0.1022	−0.2002	−0.049	0.0532	0.098	0.0756	0.0448	0.22628	−0.09947	0.2066	0.3808	0.2937	1.8431	0.5426
11(F)	−0.0861	−0.0868	−0.0856	0.0006	0.0006	0.0006	0.0001	0.00144	−0.00104	0.0022	0.0024	0.0023	1.1242	0.8895
12(F)	−0.0961	−0.0966	−0.0956	0.0005	0.0005	0.0005	0	0.0011	−0.00097	0.002	0.0019	0.0019	0.9244	1.0818
13(F)	−0.0961	−0.0966	−0.0956	0.0005	0.0005	0.0005	0	0.0011	−0.00097	0.002	0.0019	0.0019	0.9244	1.0818
14(O)	−0.1423	−0.1448	−0.1398	0.0025	0.0025	0.0025	0.0001	0.00585	−0.00462	0.0096	0.0099	0.0097	1.0256	0.975
15(O)	−0.1362	−0.1491	−0.1267	0.0095	0.0129	0.0112	0.0034	0.02976	−0.01771	0.0368	0.0501	0.0434	1.3614	0.7345
16(O)	−0.1464	−0.1469	−0.146	0.0004	0.0005	0.0004	0.0001	0.0011	−0.00074	0.0015	0.0019	0.0017	1.2134	0.8241
17(O)	−0.1496	−0.2779	0	0.1496	0.1283	0.139	−0.0212	0.29633	−0.27981	0.5812	0.4987	0.5399	0.858	1.1655
18(O)	−0.265	−0.5089	0.1535	0.4185	0.2438	0.3312	−0.1747	0.56303	−0.78292	1.6262	0.9475	1.2868	0.5826	1.7163
19(C)	0.2595	0.2558	0.263	0.0035	0.0037	0.0036	0.0002	0.00854	−0.00651	0.0135	0.0144	0.0139	1.0628	0.9409
20(C)	0.2598	0.258	0.2617	0.0019	0.0018	0.0018	−0.0001	0.00418	−0.00352	0.0073	0.007	0.0072	0.9608	1.0408
21(C)	0.2585	0.2484	0.2698	0.0113	0.0101	0.0107	−0.0011	0.02338	−0.02105	0.0437	0.0393	0.0415	0.8998	1.1114
22(C)	0.258	0.2575	0.2584	0.0005	0.0005	0.0005	0	0.00116	−0.00088	0.0018	0.002	0.0019	1.0662	0.9379
23(C)	0.2721	0.2138	0.3125	0.0405	0.0582	0.0494	0.0178	0.13448	−0.07572	0.1573	0.2263	0.1918	1.4388	0.695
24(C)	0.3609	0.3605	0.3614	0.0004	0.0004	0.0004	0	0.00094	−0.00079	0.0016	0.0016	0.0016	0.957	1.0449
25(C)	0.2138	−0.0088	0.3366	0.1228	0.2226	0.1727	0.0998	0.514	−0.22967	0.477	0.865	0.671	1.8132	0.5515
26(H)	0.2094	0.15	0.2857	0.0763	0.0594	0.0678	−0.0169	0.13707	−0.14275	0.2965	0.2307	0.2636	0.778	1.2854

**Note:** q(N): Hirshfeld charges in neutral state; q(N+1): Hirshfeld charges in a positive charge state; q(N−1): Hirshfeld charges in a negative charge state; *f*_(r)_^−^: electrophilic Fukui function; *f*_(r)_*^+^*: nucleophilic Fukui function; *f*_(r)_^0^: radical Fukui function; Δ*f*_(r)_: condensed dual descriptors; electrophilicity: reduced local electrophilic index; nucleophilicity: reduced local nucleophilic index; *s^−^*: reduced local softness; *s^+^*: relative electrophilic index*; s*_0_: relative nucleophilic index; E(N): −1940.870842 Hartree; E(N+1): −1940.951158 Hartree; E(N−1): −1940.53318 Hartree; E_HOMO(N): −0.403948 Hartree, −10.992 eV; E_HOMO(N+1): −0.157532 Hartree, −4.2867 eV; E_HOMO(N−1): −0.480193 Hartree, −13.0667 eV; vertical IP: 0.337662 Hartree, 9.1883 eV; vertical EA: 0.080315 Hartree, 2.1855 eV; Mulliken electronegativity: 0.208989 Hartree, 5.6869 eV; chemical potential: −0.208989 Hartree, −5.6869 eV; hardness (=fundamental gap): 0.257347 Hartree, 7.0028 eV; softness: 3.885805 Hartree^−1^, 0.1428 eV^−1^; electrophilicity index: 0.084859 Hartree, 2.3091 eV; nucleophilicity index: −0.06875 Hartree, −1.8708 eV.

Table A33 shows the detailed CDFT descriptors, indicating the possible reactive sites of the PFHpA molecule.

**Table A33:** Detailed information of CDFT descriptors of PFHpA.

Atom	q(N)	q(N + 1)	q(N − 1)	*f*_(r)_ *^−^*	*f*_(r)_ *^+^*	*f*_(r)_^0^	Δ*f*_(r)_	Electrophilicity	Nucleophilicity	*s^−^*	*s^+^*	*s*_0_	*s^+^/s^−^*	*s^−^/s^+^*
1(F)	−0.092	−0.1029	−0.0775	0.0144	0.0109	0.0127	−0.0035	0.02664	−0.02451	0.0575	0.0435	0.0505	0.7554	1.3238
2(F)	−0.092	−0.1029	−0.0775	0.0144	0.0109	0.0127	−0.0035	0.02664	−0.02451	0.0575	0.0435	0.0505	0.7554	1.3238
3(F)	−0.0912	−0.0971	−0.0838	0.0073	0.006	0.0067	−0.0014	0.01455	−0.01245	0.0292	0.0237	0.0265	0.8123	1.231
4(F)	−0.0912	−0.0971	−0.0838	0.0073	0.006	0.0067	−0.0014	0.01455	−0.01245	0.0292	0.0237	0.0265	0.8123	1.231
5(F)	−0.0923	−0.1189	−0.064	0.0283	0.0267	0.0275	−0.0017	0.06507	−0.04802	0.1127	0.1061	0.1094	0.9416	1.062
6(F)	−0.0923	−0.1189	−0.064	0.0283	0.0267	0.0275	−0.0017	0.06507	−0.04802	0.1127	0.1061	0.1094	0.9416	1.062
7(F)	−0.0919	−0.0951	−0.0881	0.0039	0.0032	0.0035	−0.0007	0.0078	−0.00656	0.0154	0.0127	0.0141	0.8257	1.2111
8(F)	−0.0919	−0.0951	−0.0881	0.0039	0.0032	0.0035	−0.0007	0.0078	−0.00656	0.0154	0.0127	0.0141	0.8257	1.2111
9(F)	−0.0949	−0.2159	−0.0116	0.0833	0.121	0.1022	0.0376	0.2953	−0.14136	0.3319	0.4817	0.4068	1.4516	0.6889
10(F)	−0.0949	−0.2159	−0.0116	0.0833	0.121	0.1022	0.0376	0.2953	−0.14136	0.3319	0.4817	0.4068	1.4516	0.6889
11(F)	−0.0921	−0.0948	−0.0889	0.0032	0.0027	0.0029	−0.0005	0.00657	−0.00543	0.0128	0.0107	0.0117	0.8405	1.1898
12(F)	−0.0916	−0.0933	−0.0895	0.0021	0.0018	0.0019	−0.0003	0.0043	−0.00355	0.0083	0.007	0.0077	0.8417	1.188
13(F)	−0.0916	−0.0933	−0.0895	0.0021	0.0018	0.0019	−0.0003	0.0043	−0.00355	0.0083	0.007	0.0077	0.8417	1.188
14(O)	−0.1486	−0.2675	−0.0093	0.1393	0.119	0.1291	−0.0203	0.2904	−0.2362	0.5545	0.4737	0.5141	0.8543	1.1705
15(O)	−0.2623	−0.4743	0.0527	0.3149	0.212	0.2635	−0.1029	0.51754	−0.5342	1.2541	0.8443	1.0492	0.6732	1.4854
16(C)	0.1811	0.1783	0.1879	0.0068	0.0028	0.0048	−0.004	0.00683	−0.01156	0.0271	0.0111	0.0191	0.4107	2.4349
17(C)	0.1812	0.1787	0.1847	0.0035	0.0026	0.003	−0.0009	0.00624	−0.00588	0.0138	0.0102	0.012	0.737	1.3568
18(C)	0.1818	0.1779	0.1917	0.0099	0.0039	0.0069	−0.0061	0.00946	−0.01686	0.0396	0.0154	0.0275	0.3901	2.5633
19(C)	0.1771	0.1756	0.1788	0.0018	0.0014	0.0016	−0.0003	0.00348	−0.00297	0.007	0.0057	0.0063	0.8129	1.2301
20(C)	0.1909	0.1221	0.2518	0.0609	0.0689	0.0649	0.008	0.16812	−0.10327	0.2424	0.2743	0.2583	1.1313	0.884
21(C)	0.2789	0.2776	0.2804	0.0015	0.0013	0.0014	−0.0002	0.00314	−0.00259	0.0061	0.0051	0.0056	0.8442	1.1846
22(C)	0.2117	0.0228	0.3246	0.1129	0.1889	0.1509	0.076	0.46115	−0.19145	0.4495	0.7523	0.6009	1.6738	0.5975
23(H)	0.2095	0.1526	0.2759	0.0664	0.057	0.0617	−0.0094	0.13902	−0.11256	0.2642	0.2268	0.2455	0.8582	1.1652

**Note:** q(N): Hirshfeld charges in neutral state; q(N+1): Hirshfeld charges in a positive charge state; q(N−1): Hirshfeld charges in a negative charge state; *f*_(r)_^−^: electrophilic Fukui function; *f*_(r)_*^+^*: nucleophilic Fukui function; *f*_(r)_^0^: radical Fukui function; Δ*f*_(r)_: condensed dual descriptors; electrophilicity: reduced local electrophilic index; nucleophilicity: reduced local nucleophilic index; *s^−^*: reduced local softness; *s^+^*: relative electrophilic index*; s*_0_: relative nucleophilic index; E(N): −1715.227813 Hartree; E(N+1): −1715.314508 Hartree; E(N−1): −1714.889987 Hartree; E_HOMO(N): −0.397531 Hartree, −10.8174 eV; E_HOMO(N+1):−0.165856 Hartree, −4.5132 eV; E_HOMO(N−1): −0.444055 Hartree, −12.0834 eV; vertical IP: 0.337826 Hartree, 9.1927 eV; Vertical EA: 0.086695 Hartree, 2.3591 eV; Mulliken electronegativity: 0.21226 Hartree, 5.7759 eV; chemical potential: −0.21226 Hartree, −5.7759 eV; hardness (=fundamental gap): 0.251131 Hartree, 6.8336 eV; softness: 3.981982 Hartree^−1^, 0.1463 eV^−1^; electrophilicity index: 0.089703 Hartree, 2.4409 eV; nucleophilicity index: −0.062333 Hartree, −1.6962 eV.

Table A34 shows detailed CDFT descriptors, indicating the possible reactive sites of the DFSA molecule.

**Table A34:** Detailed information of CDFT descriptors of DFSA.

Atom	q(N)	q(N + 1)	q(N − 1)	*f*_(r)_ *^−^*	*f*_(r)_ *^+^*	*f*_(r)_^0^	Δ*f*_(r)_	Electrophilicity	Nucleophilicity	*s^−^*	*s^+^*	*s*_0_	*s^+^/s^−^*	*s^−^/s^+^*
1(F)	−0.0929	−0.1018	−0.0715	0.0214	0.0089	0.0152	−0.0125	0.01727	−0.03423	0.0715	0.0298	0.0506	0.4174	2.3959
2(F)	−0.0929	−0.1018	−0.0715	0.0214	0.0089	0.0152	−0.0125	0.01727	−0.03423	0.0715	0.0298	0.0506	0.4174	2.3959
3(F)	−0.0929	−0.1018	−0.0715	0.0214	0.0089	0.0152	−0.0125	0.01727	−0.03423	0.0715	0.0298	0.0506	0.4174	2.3959
4(F)	−0.0929	−0.1018	−0.0715	0.0214	0.0089	0.0152	−0.0125	0.01727	−0.03423	0.0715	0.0298	0.0506	0.4174	2.3959
5(F)	−0.0928	−0.1075	−0.0674	0.0254	0.0147	0.0201	−0.0107	0.02848	−0.04068	0.0849	0.0492	0.067	0.5792	1.7265
6(F)	−0.0928	−0.1075	−0.0674	0.0254	0.0147	0.0201	−0.0107	0.02848	−0.04068	0.0849	0.0492	0.067	0.5792	1.7265
7(F)	−0.0928	−0.1075	−0.0674	0.0254	0.0147	0.0201	−0.0107	0.02848	−0.04068	0.0849	0.0492	0.067	0.5792	1.7265
8(F)	−0.0928	−0.1075	−0.0674	0.0254	0.0147	0.0201	−0.0107	0.02848	−0.04068	0.0849	0.0492	0.067	0.5792	1.7265
9(F)	−0.0951	−0.1467	−0.055	0.0402	0.0515	0.0459	0.0113	0.09964	−0.06429	0.1342	0.1721	0.1531	1.2824	0.7798
10(F)	−0.0951	−0.1467	−0.055	0.0402	0.0515	0.0459	0.0113	0.09964	−0.06429	0.1342	0.1721	0.1531	1.2824	0.7798
11(F)	−0.0951	−0.1467	−0.055	0.0402	0.0515	0.0459	0.0113	0.09964	−0.06429	0.1342	0.1721	0.1531	1.2824	0.7798
12(F)	−0.0951	−0.1467	−0.055	0.0402	0.0515	0.0459	0.0113	0.09964	−0.06429	0.1342	0.1721	0.1531	1.2824	0.7798
13(O)	−0.1488	−0.2127	−0.084	0.0648	0.0639	0.0643	−0.0008	0.12358	−0.10359	0.2162	0.2134	0.2148	0.987	1.0131
14(O)	−0.1488	−0.2127	−0.084	0.0648	0.0639	0.0643	−0.0008	0.12358	−0.10359	0.2162	0.2134	0.2148	0.987	1.0131
15(O)	−0.2624	−0.3758	−0.1248	0.1376	0.1134	0.1255	−0.0242	0.21928	−0.2201	0.4595	0.3787	0.4191	0.8243	1.2132
16(O)	−0.2624	−0.3758	−0.1248	0.1376	0.1134	0.1255	−0.0242	0.21928	−0.2201	0.4595	0.3787	0.4191	0.8243	1.2132
17(C)	0.1805	0.1772	0.1904	0.0099	0.0033	0.0066	−0.0067	0.00633	−0.0159	0.0332	0.0109	0.0221	0.3293	3.0368
18(C)	0.1805	0.1772	0.1904	0.0099	0.0033	0.0066	−0.0067	0.00633	−0.0159	0.0332	0.0109	0.0221	0.3293	3.0368
19(C)	0.1814	0.1778	0.1904	0.009	0.0036	0.0063	−0.0054	0.00695	−0.0144	0.0301	0.012	0.021	0.3991	2.5055
20(C)	0.1814	0.1778	0.1904	0.009	0.0036	0.0063	−0.0054	0.00695	−0.0144	0.0301	0.012	0.021	0.3991	2.5055
21(C)	0.1908	0.1604	0.2148	0.024	0.0304	0.0272	0.0064	0.05874	−0.03836	0.0801	0.1015	0.0908	1.2671	0.7892
22(C)	0.1908	0.1604	0.2148	0.024	0.0304	0.0272	0.0064	0.05874	−0.03836	0.0801	0.1015	0.0908	1.2671	0.7892
23(C)	0.2116	0.1077	0.2627	0.051	0.1039	0.0775	0.0529	0.20088	−0.08163	0.1704	0.3469	0.2587	2.036	0.4912
24(C)	0.2116	0.1077	0.2627	0.051	0.1039	0.0775	0.0529	0.20088	−0.08163	0.1704	0.3469	0.2587	2.036	0.4912
25(H)	0.2093	0.1789	0.2389	0.0297	0.0304	0.03	0.0008	0.05882	−0.04743	0.099	0.1016	0.1003	1.026	0.9747
26(H)	0.2093	0.1789	0.2389	0.0297	0.0304	0.03	0.0008	0.05882	−0.04743	0.099	0.1016	0.1003	1.026	0.9747

**Note:** q(N): Hirshfeld charges in neutral state; q(N+1): Hirshfeld charges in a positive charge state; q(N−1): Hirshfeld charges in a negative charge state; *f*_(r)_^−^: electrophilic Fukui function; *f*_(r)_*^+^*: nucleophilic Fukui function; *f*_(r)_^0^: radical Fukui function; Δ*f*_(r)_: condensed dual descriptors; electrophilicity: reduced local electrophilic index; nucleophilicity: reduced local nucleophilic index; *s^−^*: reduced local softness; *s^+^*: relative electrophilic index*; s*_0_: relative nucleophilic index; E(N): −1804.507374 Hartree; E(N+1): −1804.563939 Hartree; E(N−1): −1804.151354 Hartree; E_HOMO(N): −0.393987 Hartree, −10.7209 eV; E_HOMO(N+1): −0.101246 Hartree, −2.755 eV; E_HOMO(N−1): −0.364223 Hartree, −9.911 eV; vertical IP: 0.35602 Hartree, 9.6878 eV; vertical EA: 0.056566 Hartree, 1.5392 eV; Mulliken electronegativity: 0.206293 Hartree, 5.6135 eV; chemical potential: −0.206293 Hartree, −5.6135 eV; hardness (=fundamental gap): 0.299454 Hartree, 8.1486 eV; softness: 3.339412 Hartree^−1^, 0.1227 eV^−1^; electrophilicity index: 0.071057 Hartree, 1.9336 eV; nucleophilicity index: −0.058789 Hartree, −1.5997 eV.

## References

[1] Gao K., Zhuang T., Liu X., Fu J., Zhang J., Fu J., Wang L., Zhang A., Liang Y., Song M. (2019). Prenatal Exposure to Per- and Polyfluoroalkyl Substances (PFASs) and Association between the Placental Transfer Efficiencies and Dissociation Constant of Serum Proteins–PFAS Complexes. Environ. Sci. Technol..

[2] Lu Y., Meng L., Ma D., Cao H., Liang Y., Liu H., Wang Y., Jiang G. (2021). The occurrence of PFAS in human placenta and their binding abilities to human serum albumin and organic anion transporter 4. Environ. Pollut..

[3] Zhao L., Teng M., Zhao X., Li Y., Sun J., Zhao W., Ruan Y., Leung K.M.Y., Wu F. (2023). Insight into the binding model of per- and polyfluoroalkyl substances to proteins and membranes. Environ. Int..

[4] Liu X., Fang M., Xu F., Chen D. (2019). Characterization of the binding of per- and poly-fluorinated substances to proteins: A methodological review. TrAC Trends Anal. Chem..

[5] Fedorenko M., Alesio J., Fedorenko A., Slitt A., Bothun G.D. (2021). Dominant entropic binding of perfluoroalkyl substances (PFASs) to albumin protein revealed by 19F NMR. Chemosphere.

[6] Tian Y., Zhou Q., Zhang L., Li W., Yin S., Li F., Xu C. (2023). In utero exposure to per-/polyfluoroalkyl substances (PFASs): Preeclampsia in pregnancy and low birth weight for neonates. Chemosphere.

[7] Chi Q., Li Z., Huang J., Ma J., Wang X. (2018). Interactions of perfluorooctanoic acid and perfluorooctanesulfonic acid with serum albumins by native mass spectrometry, fluorescence and molecular docking. Chemosphere.

[8] Calore F., Guolo P.P., Wu J., Xu Q., Lu J., Marcomini A. (2023). Legacy and novel PFASs in wastewater, natural water, and drinking water: Occurrence in Western Countries vs China. Emerg. Contam..

[9] An X., Lei H., Lu Y., Xie X., Wang P., Liao J., Liang Z., Sun B., Wu Z. (2023). Per- and polyfluoroalkyl substances (PFASs) in water and sediment from a temperate watershed in China: Occurrence, sources, and ecological risks. Sci. Total Environ..

[10] Xu L., Chen H., Han X., Yu K., Wang Y., Du B., Zeng L. (2022). First report on per- and polyfluoroalkyl substances (PFASs) in coral communities from the Northern South China sea: Occurrence, seasonal variation, and interspecies differences. Environ. Pollut..

[11] Jian J.-M., Chen D., Han F.-J., Guo Y., Zeng L., Lu X., Wang F. (2018). A short review on human exposure to and tissue distribution of per- and polyfluoroalkyl substances (PFASs). Sci. Total Environ..

[12] Li X.-Y., Huang Z.-Y., Niu Y., Wang Z.-H., Hu L.-Y., Bai A.-M., Hu Y.-J. (2022). Synthesis of a IAP antagonist analogue and its binding investigation with BSA/HSA. J. Mol. Struct..

[13] Chen H., He P., Rao H., Wang F., Liu H., Yao J. (2015). Systematic investigation of the toxic mechanism of PFOA and PFOS on bovine serum albumin by spectroscopic and molecular modeling. Chemosphere.

[14] Sen P., Karn R., Kanake D.W., Emerson I.A., Khan J.M., Ahmad A. (2023). Picloram binds to the h1 and h4 helices of HSA domain IIIA at drug binding site 2. Int. J. Biol. Macromol..

[15] Chugh H., Kumar P., Tomar V., Kaur N., Sood D., Chandra R. (2019). Interaction of noscapine with human serum albumin (HSA): A spectroscopic and molecular modelling approach. J. Photochem. Photobiol. A Chem..

[16] Meng X., Yu G., Luo T., Zhang R., Zhang J., Liu Y. (2023). Transcriptomics integrated with metabolomics reveals perfluorobutane sulfonate (PFBS) exposure effect during pregnancy and lactation on lipid metabolism in rat offspring. Chemosphere.

[17] Iwata H., Kobayashi S., Itoh M., Itoh S., Mesfin Ketema R., Tamura N., Miyashita C., Yamaguchi T., Yamazaki K., Masuda H. (2023). The association between prenatal per-and polyfluoroalkyl substance levels and Kawasaki disease among children of up to 4 years of age: A prospective birth cohort of the Japan Environment and Children’s Study. Environ. Int..

[18] Peng M., Wang Y., Wu C., Cai X., Wu Y., Du E., Zheng L., Fu J. (2023). Investigating sulfonamides—Human serum albumin interactions: A comprehensive approach using multi-spectroscopy, DFT calculations, and molecular docking. Biochem. Biophys. Res. Commun..

[19] Negrea E., Oancea P., Leonties A., Ana Maria U., Avram S., Raducan A. (2023). Spectroscopic studies on binding of ibuprofen and drotaverine with bovine serum albumin. J. Photochem. Photobiol. A Chem..

[20] Neamtu S., Mic M., Bogdan M., Turcu I. (2013). The artifactual nature of stavudine binding to human serum albumin. A fluorescence quenching and isothermal titration calorimetry study. J. Pharm. Biomed. Anal..

[21] Miles A.J., Ramalli S.G., Wallace B.A. (2022). DichroWeb, a website for calculating protein secondary structure from circular dichroism spectroscopic data. Protein Sci..

[22] Gan N., Sun Q., Tang P., Wu D., Xie T., Zhang Y., Li H. (2019). Determination of interactions between human serum albumin and niraparib through multi-spectroscopic and computational methods. Spectrochim. Acta Part A Mol. Biomol. Spectrosc..

[23] Ali M.S., Muthukumaran J., Jain M., Santos-Silva T., Al-Lohedan H.A., Al-Shuail N.S. (2021). Molecular interactions of cefoperazone with bovine serum albumin: Extensive experimental and computational investigations. J. Mol. Liq..

[24] Frisch M., Trucks G., Schlegel H., Scuseria G., Robb M., Cheeseman J., Scalmani G., Barone V., Petersson G., Nakatsuji H. (2016). Gaussian 16 Rev. C. 01.

[25] Fu W., Xia G.-J., Zhang Y., Hu J., Wang Y.-G., Li J., Li X., Li B. (2021). Using general computational chemistry strategy to unravel the reactivity of emerging pollutants: An example of sulfonamide chlorination. Water Res..

[26] Wen J., Li L., Yuan L., Li J., Ning P. (2022). Insight into the weak interaction between organic primary amine and propionic acid or phenol solvents in solvent extraction. J. Mol. Liq..

[27] Sahoo D.K., Dasgupta S., Kistwal T., Datta A. (2023). Fluorescence monitoring of binding of a Zn (II) complex of a Schiff base with human serum albumin. Int. J. Biol. Macromol..

[28] Lu T., Chen F. (2012). Multiwfn: A multifunctional wavefunction analyzer. J. Comput. Chem..

[29] Humphrey W., Dalke A., Schulten K. (1996). VMD: Visual molecular dynamics. J. Mol. Graph..

[30] Radha A., Singh A., Sharma L., Thakur K.K. (2021). Molecular interactions of acebutolol hydrochloride to human serum albumin: A combined calorimetric, spectroscopic and molecular modelling approach. Mater. Today Proc..

[31] Eberhardt J., Santos-Martins D., Tillack A.F., Forli S. (2021). AutoDock Vina 1.2.0: New Docking Methods, Expanded Force Field, and Python Bindings. J. Chem. Inf. Model..

[32] Trott O., Olson A.J. (2010). AutoDock Vina: Improving the speed and accuracy of docking with a new scoring function, efficient optimization, and multithreading. J. Comput. Chem..

[33] Schrödinger, Inc. (2015). The AxPyMOL Molecular Graphics Plugin for Microsoft PowerPoint, Version 1.8.

[34] Salomon-Ferrer R., Case D.A., Walker R.C. (2013). An overview of the Amber biomolecular simulation package. Wiley Interdiscip. Rev. Comput. Mol. Sci..

[35] Wang J., Wolf R.M., Caldwell J.W., Kollman P.A., Case D.A. (2004). Development and testing of a general amber force field. J. Comput. Chem..

[36] Maier J.A., Martinez C., Kasavajhala K., Wickstrom L., Hauser K.E., Simmerling C. (2015). ff14SB: Improving the Accuracy of Protein Side Chain and Backbone Parameters from ff99SB. J. Chem. Theory Comput..

[37] Mark P., Nilsson L. (2001). Structure and dynamics of the TIP3P, SPC, and SPC/E water models at 298 K. J. Phys. Chem. A.

[38] Sagui C., Darden T.A. (1999). Molecular dynamics simulations of biomolecules: Long-range electrostatic effects. Annu. Rev. Biophys. Biomol. Struct..

[39] Kräutler V., Van Gunsteren W.F., Hünenberger P.H. (2001). A fast SHAKE algorithm to solve distance constraint equations for small molecules in molecular dynamics simulations. J. Comput. Chem..

[40] Larini L., Mannella R., Leporini D. (2007). Langevin stabilization of molecular-dynamics simulations of polymers by means of quasisymplectic algorithms. J. Chem. Phys..

[41] Hou T., Wang J., Li Y., Wang W. (2011). Assessing the performance of the MM/PBSA and MM/GBSA methods. 1. The accuracy of binding free energy calculations based on molecular dynamics simulations. J. Chem. Inf. Model..

[42] Genheden S., Ryde U. (2015). The MM/PBSA and MM/GBSA methods to estimate ligand-binding affinities. Expert Opin. Drug Discov..

[43] Rastelli G., Rio A.D., Degliesposti G., Sgobba M. (2010). Fast and accurate predictions of binding free energies using MM-PBSA and MM-GBSA. J. Comput. Chem..

[44] Chen Y., Zheng Y., Fong P., Mao S., Wang Q. (2020). The application of the MM/GBSA method in the binding pose prediction of FGFR inhibitors. Phys. Chem. Chem. Phys..

[45] Nguyen H., Roe D.R., Simmerling C. (2013). Improved Generalized Born Solvent Model Parameters for Protein Simulations. J. Chem. Theory Comput..

[46] Weiser J., Shenkin P.S., Still W.C. (1999). Approximate atomic surfaces from linear combinations of pairwise overlaps (LCPO). J. Comput. Chem..

[47] Bagheri M., Fatemi M.H. (2018). Fluorescence spectroscopy, molecular docking and molecular dynamic simulation studies of HSA-Aflatoxin B1 and G1 interactions. J. Lumin..

[48] Rostamnezhad F., Hossein Fatemi M. (2022). Comprehensive investigation of binding of some polycyclic aromatic hydrocarbons with bovine serum albumin: Spectroscopic and molecular docking studies. Bioorg. Chem..

[49] Li W., Chen S., Hong X., Fang M., Zong W., Li X., Wang J. (2023). The molecular interaction of three haloacetic acids with bovine serum albumin and the underlying mechanisms. J. Mol. Liq..

[50] Gu J., Huang X., Liu H., Dong D., Sun X. (2022). A mutispectroscopic study on the structure–affinity relationship of the interactions of bisphenol analogues with bovine serum albumin. Chemosphere.

[51] Chang S., Qin D., Wang L., Zhang M., Yan R., Zhao C. (2021). Preparation of novel cinnamaldehyde derivative–BSA nanoparticles with high stability, good cell penetrating ability, and promising anticancer activity. Colloids Surf. A Physicochem. Eng. Asp..

[52] Ovung A., Bhattacharyya J. (2022). Binding effects of antibiotic drug sulfamethazine on the serum albumins: Multi-spectroscopic and computation approach. Chem. Phys. Impact.

[53] Zhu M., Pang X., Wan J., Xu X., Wei X., Hua R., Zhang X., Wang Y., Yang X. (2022). Potential toxic effects of sulfonamides antibiotics: Molecular modeling, multiple-spectroscopy techniques and density functional theory calculations. Ecotoxicol. Environ. Saf..

[54] Farajzadeh-Dehkordi N., Farhadian S., Zahraei Z., Gholamian-Dehkordi N., Shareghi B. (2021). Interaction of reactive Red195 with human serum albumin: Determination of the binding mechanism and binding site by spectroscopic and molecular modeling methods. J. Mol. Liq..

[55] Ansari A. (2023). Decoding the binding interaction of steroidal pyridines with bovine serum albumin using spectroscopic and molecular docking techniques. Steroids.

[56] Liu T., Liu M., Guo Q., Liu Y., Zhao Y., Wu Y., Sun B., Wang Q., Liu J., Han J. (2020). Investigation of binary and ternary systems of human serum albumin with oxyresveratrol/piceatannol and/or mitoxantrone by multipectroscopy, molecular docking and cytotoxicity evaluation. J. Mol. Liq..

[57] Ouaket A., Chraka A., Raissouni I., Amrani M.A.E., Berrada M., Knouzi N. (2022). Synthesis, spectroscopic (13C/1H-NMR, FT-IR) investigations, quantum chemical modelling (FMO, MEP, NBO analysis), and antioxidant activity of the bis-benzimidazole molecule. J. Mol. Struct..

[58] Zhang C., Liu Y., Song F., Wang J. (2022). Inter-/intra-molecular interactions, preferential solvation, and dissolution and transfer property for tirofiban in aqueous co-solvent mixtures. J. Mol. Liq..

[59] Mishma J.N.C., Jothy V.B., Irfan A., Narayana B., Muthu S. (2023). Role of solvents in molecular level interaction, reactivity and spectral characterisation of 2-Amino-3-(((E)-4-(dimethylamino)benzylidene)amino) maleonitrile: Anti depressant agent. J. Mol. Liq..

[60] Jeba Reeda V.S., Bena Jothy V. (2023). Vibrational spectroscopic, quantum computational (DFT), reactivity (ELF, LOL and Fukui), molecular docking studies and molecular dynamic simulation on (6-methoxy-2-oxo-2H-chromen-4-yl) methyl morpholine-4-carbodithioate. J. Mol. Liq..

[61] Radder S.B., Melavanki R., Radder U., Hiremath S.M., Kusanur R., Khemalapure S.S. (2022). Synthesis, spectroscopic (FT-IR, FT-Raman, NMR), reactivity (ELF, LOL and Fukui) and docking studies on 3-(2-hydroxy-3-methoxy-phenyl)-1-(3-nitro-phenyl)-propenone by experimental and DFT methods. J. Mol. Struct..

[62] Bharathy G., Christian Prasana J., Muthu S., Irfan A., Basha Asif F., Saral A., Aayisha S., Niranjana Devi R. (2021). Evaluation of electronic and biological interactions between N-[4-(Ethylsulfamoyl)phenyl]acetamide and some polar liquids (IEFPCM solvation model) with Fukui function and molecular docking analysis. J. Mol. Liq..

[63] Arulaabaranam K., Muthu S., Mani G., Ben Geoffrey A.S. (2021). Speculative assessment, molecular composition, PDOS, topology exploration (ELF, LOL, RDG), ligand-protein interactions, on 5-bromo-3-nitropyridine-2-carbonitrile. Heliyon.

[64] Steffy A.D., Dhas D.A., Joe I.H., Balachandran S. (2024). Theoretical investigations on structural, spectral, NBO, NLO and topology exploration (AIM, ELF, LOL, RDG) of piperazine-2,5-dione oxalic acid monohydrate. J. Mol. Struct..

[65] Sagaama A., Issaoui N., Al-Dossary O., Kazachenko A.S., Wojcik M.J. (2021). Non covalent interactions and molecular docking studies on morphine compound. J. King Saud Univ. -Sci..

[66] Lu T., Chen Q. (2021). Interaction region indicator: A simple real space function clearly revealing both chemical bonds and weak interactions. Chemistry-Methods.

[67] Islam M.M., Sonu V.K., Gashnga P.M., Moyon N.S., Mitra S. (2016). Caffeine and sulfadiazine interact differently with human serum albumin: A combined fluorescence and molecular docking study. Spectrochim. Acta Part A.

[68] Zhang S., Gan R., Zhao L., Sun Q., Xiang H., Xiang X., Zhao G., Li H. (2021). Unveiling the interaction mechanism of alogliptin benzoate with human serum albumin: Insights from spectroscopy, microcalorimetry, and molecular docking and molecular dynamics analyses. Spectrochim. Acta Part A Mol. Biomol. Spectrosc..

[69] Zhan F., Ding S., Xie W., Zhu X., Hu J., Gao J., Li B., Chen Y. (2020). Towards understanding the interaction of β-lactoglobulin with capsaicin: Multi-spectroscopic, thermodynamic, molecular docking and molecular dynamics simulation approaches. Food Hydrocoll..

[70] Kharazian B., Ahmad A.A., Mabudi A. (2021). A molecular dynamics study on the binding of gemcitabine to human serum albumin. J. Mol. Liq..

[71] Cao J., Li L., Xiong L., Wang C., Chen Y., Zhang X. (2022). Research on the mechanism of berberine in the treatment of COVID-19 pneumonia pulmonary fibrosis using network pharmacology and molecular docking. Phytomed. Plus.

[72] Weatherly L.M., Shane H.L., Lukomska E., Baur R., Anderson S.E. (2023). Systemic toxicity induced by topical application of perfluoroheptanoic acid (PFHpA), perfluorohexanoic acid (PFHxA), and perfluoropentanoic acid (PFPeA) in a murine model. Food Chem. Toxicol..

[73] Smeltz M., Wambaugh J.F., Wetmore B.A. (2023). Plasma Protein Binding Evaluations of Per- and Polyfluoroalkyl Substances for Category-Based Toxicokinetic Assessment. Chem. Res. Toxicol..

[74] Starnes H.M., Jackson T.W., Rock K.D., Belcher S.M. (2023). Quantitative Cross-Species Comparison of Serum Albumin Binding of Per- and Polyfluoroalkyl Substances from Five Structural Classes. bioRxiv.

